# Mobile Apps to Enhance Health Literacy in Children and Adolescents With Injury or Disease to the Brain or Spinal Cord: Scoping Review

**DOI:** 10.2196/101750

**Published:** 2026-11-13

**Authors:** Ingrid Eitzen, Lene Mosberg, Anne Catrine Martinsen, Julia Bidonde, Edel Jannecke Svendsen

**Affiliations:** 1 Centre for Research and Education, Sunnaas Rehabilitation Hospital, Bjørnemyr, Akershus, Norway; 2 Department of Technology and Digitalization, South-Eastern Norway Regional Health Authority, Oslo, Norway; 3 Department of Rehabilitation Science and Health Technology, Faculty of Health Sciences, OsloMet—Oslo Metropolitan University, Oslo, Norway; 4 School of Rehabilitation Science, College of Medicine, University of Saskatchewan, Saskatoon, SK, Canada; 5 Department of Nursing and Health Promotion, Faculty of Health Sciences, OsloMet—Oslo Metropolitan University, Pilestredet 32, Oslo, 0166, Norway, 47 924 20 939

**Keywords:** health literacy, digital health literacy, digital tools, mobile apps, brain injury, spinal cord injury, diseases in the central nervous system, self-management, children, adolescents, young adults

## Abstract

**Background:**

Young individuals who are affected by an injury or disease to the brain or spinal cord must manage diverse functional and cognitive challenges. Digital tools can increase the users’ understanding and management of their daily lives through increased health literacy. However, to realize this potential, we need to know which tools are available, whether they are tailored for end users, and whether they are developed and evaluated with scientific evidence.

**Objective:**

This study aimed to map evidence from published literature assessing digital tools in the form of mobile apps and interactive e-learning resources developed to increase health literacy for a target population of young individuals aged 5 to 25 years with injury or disease to the brain or spinal cord. Furthermore, it aimed to identify available apps and e-learning resources, and describe their features, intended use, and privacy issues.

**Methods:**

Following the Joanna Briggs Institute framework for scoping reviews, we conducted a search across 10 scientific databases. For gray literature, we searched Google, Google Scholar, and 15 selected websites of organizations affiliated with our target population. To identify apps, we used Fnd.io for the App Store and Google Play. Sources had to address our target population, involve digital tools with active engagement of the end user, and be published or updated within the last 6 years.

**Results:**

Of 821 identified scientific records from databases, 2 met our eligibility criteria. The gray literature search resulted in 6 more eligible publications, giving a final number of 8 included papers: 3 randomized controlled trials, 3 reviews, and 2 theses. Across intervention studies, sample sizes ranged from 7 to 150 participants; intervention durations ranged from 2 weeks to 9 months. App searches identified 114 apps, of which 84 were excluded due to the following reasons: duplicates (n=10), wrong outcome (n=42), wrong population (n=26), no update within 6 years (n=4), and no longer available (n=2). Of the 30 included apps, 14 targeted brain conditions, 10 spinal cord conditions, and 6 both. A total of 16 apps reported affiliation with an organization, 12 explicitly required health professional involvement, 23 were designed primarily for home use, 23 were free, 18 were available on both iOS and Android, and 20 had been updated within the last 2 years. All apps linked to a privacy policy, 8 stated third-party authorizations, and 1 app reported full electronic health record integration.

**Conclusions:**

Evidence on digital tools to improve health literacy in young people with brain or spinal cord conditions is limited. Only 8 publications and 30 apps met eligibility criteria. Available apps emphasize education, symptom tracking, and self-management, but evidence for efficacy is scarce, and privacy information is often difficult to interpret. More rigorous, user-centered evaluation of safe and effective digital tools is needed.

## Introduction

The context of health services delivery is increasingly shifting from hospitals to patients’ homes, supported by a growing expectation of digitalization. Today, more and more health information is being disseminated online, and health services are increasingly being provided through digital platforms [1]. As this transformation accelerates, patients’ digital competence becomes essential for their ability to understand, use, and interact with digital tools in the form of computers, mobile phones, and apps [2,3]. For individuals who are born with a lifelong health condition or sustain a serious injury early in life, understanding one’s own health condition is essential for effective management of care and decision-making around care options. By making tailored information about specific conditions accessible to those individuals, digital tools can play an important role in their health and well-being. Digital tools can assist in defining goals, tracking progress, and providing feedback, and interactive features can support individuals to make proactive choices which may enhance autonomy [4]. However, to fully realize this potential, it is essential that users not only understand the digital tools but also apply them in ways that align with their specific needs and circumstances. In this context, health literacy becomes a critical determinant of whether these tools can support safe, effective, and meaningful engagement.

According to the World Health Organization, *health literacy* means “being able to access, understand, appraise, and use information and services in ways that promote and maintain good health and well-being” [5]. The before mentioned digital transformation within the health care sector has introduced an extension of health literacy, namely, *digital health literacy*. Contemporary literature presents multiple definitions and understandings of digital health literacy [6], but a common interpretation is related to individuals’ use of devices such as mobile phones, tablets, and computers to navigate the internet to find, select, understand, and evaluate the health information presented in various digital formats online, and to apply this information to make informed decisions related to their health issues [3,7]. If the intended potential of a digital tool is to be realized, it is important that it is tailored for its target user [8,9]. Digital health literacy is influenced by multiple factors such as age, education, social support, and socioeconomic status [10,11]. Users who possess adequate technical and digital skills may be able to achieve a high level of digital health literacy. This can enhance patient education, strengthen self-management capabilities, and patient-provider communication [3,12], improve health outcomes, and reduce inequalities in general health [13]. Conversely, low levels of digital health literacy can have a detrimental effect: individuals with insufficient digital abilities may struggle to effectively access, comprehend, and act upon health information. This so-called digital divide [14,15] may lead to larger differences in access to health information between digitally skilled and less skilled patient populations and hence lead to increased health disparities [16].

In this scoping review, the population of interest is children, adolescents, and young adults with disease and/or injury to the central nervous system: the brain and the spinal cord. These diseases or injuries may be congenital, such as cerebral palsy (CP), hydrocephalus, and spina bifida (including myelomeningocele and myelocele), or they may be acquired following incidents such as falls, traffic incidents, tumors, or infections. In cases of multiple trauma, individuals may sustain combined injuries affecting both the brain and the spinal cord. Being affected by disease or injury from a young age will have life-altering consequences for the individual as well as their caretakers, family, and friends. Although these conditions vary widely in symptom presentation and in severity of functional and cognitive challenges, these individuals share the lifelong need to manage a complex health situation which can negatively impact quality of life (QoL) and mental health [17-20]. It seems to be agreement in current literature that ample digital health literacy can positively influence different health outcomes in individuals with chronic conditions [21-23]. But it also appears evident that there are research gaps on this topic [21,22,24], particularly on different disease groups and on specific health-related outcomes [11,25]. The need for more research is emphasized specifically also for the spinal cord injury (SCI) population to evaluate the impact and usability of available tools and make recommendations for end users [26,27]. For individuals with brain injury, mobile apps for cognitive rehabilitation are growing, but the evidence supporting their effectiveness remains limited [28]. Specifically for stroke, mobile apps often address rehabilitation, education, and self-care, but evidence on intervention effects is lacking [29].

Age is identified as a factor affecting the effectiveness of digital tools [23]. Mancone et al [30] argue that the use of digital tools is of particular importance for increased health literacy in the young population, as they in general have higher technical abilities than older populations. Digital health literacy can therefore be seen as a critical determinant of long-term health outcomes in this population, and there seems to be a clear need for customized digital tools. While young people are being identified to be receptive to and interested in receiving information and managing their health using mobile apps or websites [31], studies focusing on this population are relatively scarce [21]. There remain limited data about the effectiveness of digital interventions facilitating the self-management and health care transition of adolescents and young adults with chronic diseases [31]. Research is warranted to investigate the effectiveness of mobile-based gamification elements on health and health behavior outcomes, as well as the healthy development of children with disabilities [25].

The use of digital tools involves challenges related to privacy and security of health data. A concern in this regard is that users can lack awareness and have limited knowledge on data privacy, which may restrict their use of digital health services that have the potential to improve their health outcomes [32]. Thus, research giving insight into privacy and security issues in existing tools is warranted.

To map the existing evidence base for our topic of interest, an exploratory scoping search was conducted. No reviews were identified that specifically examined digital health literacy in the context of children, adolescents, or young adults with injuries to the brain or spinal cord. Therefore, the aim of this scoping review was to map the published knowledge on the usefulness and efficacy of digital tools for promoting health literacy in our target population, to reveal to what extent tools are developed or guided by scientific evidence, and in what regard user experiences are included and evaluated in published studies. We further aimed to identify which mobile apps and other available digital resources in the form of interactive e-learning websites are available that specifically target our user groups. Finally, we aimed to describe what characterizes the available tools with regard to platforms, availability, featured content, user contexts, and privacy and security issues. To address our aims, we developed the following research questions (RQs):

- RQ1: What evidence exists regarding the usability and efficacy of mobile apps and interactive e-learning websites aimed at improving health literacy among individuals aged 5‐25 years with congenital or acquired brain and/or spinal cord conditions?
- RQ2: What user experiences are reported in the identified studies?
- RQ3: Which mobile apps and/or interactive e-learning websites tailored to increase digital health literacy in individuals aged between 5 and 25 years with injury or disease to the brain and/or spinal cord are available?
- RQ4: What characterizes the identified tools (availability, platforms, and user interface), the user groups involved (children, adolescents, young adults, parents or caretakers, health personnel, teachers, and friends), and settings for use and implementation (hospital, rehabilitation center, school, and home)? And, in what regard is privacy and security information easily accessible?

## Methods

### Protocol and Registration

The study protocol was registered in Open Science Framework in November 2024, with amendments added in February and May 2026 [33]. Amendments were not driven by study findings but describe adjustments to the RQs, the target population age, and publication time span for inclusion of sources, as well as the paper title. We also added a table with the selected data items for reporting and the final list of inclusion and exclusion criteria. We refer to the protocol for descriptions of our methods not provided in this manuscript [33]. For the conduct of the scoping review, we followed the Joanna Briggs Institute framework for scoping reviews [34]. Results are reported according to the PRISMA-ScR (Preferred Reporting Items for Systematic Reviews and Meta-Analyses extension for Scoping Reviews; Checklist 1) [35].

### Eligibility Criteria

#### Scientific Records

The studies had the following inclusion and exclusion criteria (Textbox 1).

Textbox 1. **Inclusion and exclusion criteria.**

**Inclusion criteria:**

- Population: the studies had to include children, adolescents, and/or young adults (age range defined to 5‐25 years) with disease or injury to the brain and/or spinal cord. Records with a mixed population (children and adults) were included for review.
- Intervention: the studies had to assess digital health literacy tools, defined as a mobile app or an interactive e-learning website, where the individual with the disease or injury has an active role. Digital solutions or tools for transmission of data such as medical information and/or records only, for communication between health personnel only, for caretakers only, for trainers or teachers only, or for surveillance purposes only, were not eligible.
- Context: we had no limitations with regard to cultural factors (geographic location and social, cultural, or gender-based interests) or specific settings (eg, hospital, rehabilitation center, school, and home).
- Publication status: published journal papers, conference paper proceedings, or other published literature such as reports or theses.
- Records written in English, Spanish, or Scandinavian languages.
- Records published between 2019 and 2026. Due to the rapid development of this field, this time limit was chosen to narrow the results to relevant digital tools likely still in use. We considered that earlier developed technology, still relevant and in use, would appear in publications within the chosen time frame. The time frame finally ensures that publications evolving because of the digital transformation during the COVID-19 pandemic would be included.

**Exclusion criteria:**

- Assessing health literacy in general without the use of a digital tool in the form of a mobile app or an interactive e-learning website.
- Tools not intended for active use by the end user, that is, tools for general patient information purposes only, for transmission of medical information and/or records only, for communication between health personnel only, or for surveillance purposes only.
- Studies solely describing the development of a new digital tool from technical terms and/or user requirements, without being tested on or used by individuals (ie, prototypes).
- Reporting on adult or older adult participants only.

#### Mobile Apps and Interactive e-Learning Websites

Inclusion and exclusion criteria were identical for the identification of mobile apps and e-learning interactive webpages regarding target user groups and that apps should address enhancement of health literacy. However, the following were also applied:

- Publication year: apps first launched before 2019 were eligible if they were still available to access. If, however, their last reported updated version in the app stores was older than 6 years, they were excluded.
- Availability: apps were excluded if they were not available for download in the App Store or Google Play.

### Information Sources and Search Strategy

#### Overview

We predefined two relevant sources of information: (1) scientific papers, reports, and theses, and (2) mobile apps and interactive e-learning websites. To find type (1) information, a systematic literature search was developed and performed by an academic librarian on August 25, 2025, in MEDLINE, Embase, APA PsycINFO, AMED, ERIC, CINAHL, Cochrane Library, and Scopus. The search strategies combined controlled vocabulary (eg, MeSH in MEDLINE and Emtree in Embase) and free-text terms related to brain and SCIs, digital technologies (eg, mobile apps), health literacy, and children and adolescents. Searches were limited to publications from 2019 to 2025. In the original search, we did not include abbreviations of established specific outcome measures for digital health literacy in our search strings. In hindsight, we decided that these should be included. Furthermore, in the first search we used terms for “children and adolescents,” but to make sure we did not miss young adults, we wanted to include this as a specific term. Therefore, a new search was conducted June 16, 2026, adding these terms to the original search string. The time frame was also expanded to include publications in 2026. Detailed search strings for the final search can be found in Multimedia Appendix 1.

To find type (2) information, gray literature searches were conducted between January and April 2025 across the following sources: (1) Google Scholar, (2) Google (web search engine), (3) selected international organizational websites, (4) mobile app directories (fnd.io and Google Play), and (5) the Apple App Store. One reviewer (JB) led the searches of Google, Google Scholar, and mobile app directories activities. All searches were cross-checked by a second reviewer (LM). A third review author (IE) subsequently performed an in-depth review of the identified apps using an iPhone with iOS 26.5. Device location settings were standardized to Norway to maximize app availability and minimize geographic algorithmic variation. All apps were downloaded and reviewed from January 13 to 30, 2026. When using fnd.io, we conducted all searches through the US iOS App Store interface to ensure consistency across queries. The complete list of identified apps, including those excluded at screening, is provided in the PRISMA (Preferred Reporting Items for Systematic Reviews and Meta-Analyses) flow diagram and supporting appendices. We documented all search activities, including queries that returned no results, in a structured search log (available from the corresponding author upon request). A summary of gray literature searches can be found in Multimedia Appendix 2.

#### Google Scholar Search Approach

We conducted searches via Google Scholar [36] using a browser signed out of any Google account to minimize personalization of results. Language was set to English, and no date restrictions were applied. Given that Google Scholar does not support proximity operators or field-specific Boolean syntax equivalent to that used in the database searches, search strings were adapted for plain-language Boolean entry while retaining the full scope of population, technology, and condition terms. We used an iterative string development with an initial full term (see string 1 in Multimedia Appendix 3), which returned only 7 results reflecting Google Scholar’s algorithmic handling of long Boolean strings and its tendency to truncate complex queries; thus, we iteratively adapted the query. The sequence of strings used and the results retrieved are reported in Multimedia Appendix 3. Strings 2 through 5 progressively replaced MeSH-specific phrasing with common-language equivalents and added health literacy and patient education terms to capture the digital health literacy focus of the review. We screened the first 10 pages for each string at the title and abstract level, in accordance with standard gray literature search practice. For results that appeared highly relevant, we retrieved the full text for eligibility assessment. We logged all retrieved URLs and titles in a structured search record at the point of search.

#### Google Web Search

We conducted searches via Google [37] using a browser in private or incognito mode to minimize personalization and geographic filtering. We conducted searches in English and applied no other filters (eg, site-specific filtering, date). Google search strings were deliberately simplified relative to the database and Google Scholar strings, as Google’s web search algorithm uses semantic indexing and does not support Boolean logic in the same way. Strings followed the template: apps about [condition] and [children OR youth]. The conditions searched included brain injury, concussion, SCI, spina bifida, hydrocephalus, CP, and neurological rehabilitation (examples provided in Multimedia Appendix 3). In all cases, we reviewed only the first 2 pages of results, consistent with evidence that relevant gray literature is rarely found beyond page 2 in Google searches. For each search, we recorded the page title, URL, and a brief note on relevance. Where a webpage contained a list of app recommendations (eg, a condition-specific page recommending apps for patients), we screened the full list for potentially eligible apps and each was individually logged.

#### Organizational Websites

We also conducted searches in 15 websites of organizations involved in research, disability, health literacy, and technology development. The selection of organizational websites was based on the review authors’ broad clinical experience within the fields of SCI, brain injuries, and rehabilitation, and was agreed upon by the full review team before searching. For each website, the internal search function was used where available, supplemented by manual browsing of relevant sections such as resources, tools, publications, resources, and app libraries. Where no internal search function was available or functional, the site was browsed manually using its navigational structure (Ctrl + F and specific words such as apps, applications, digital, patient education, digital health literacy, or Norwegian words digital, pasientundervisning, helsekompetanse, læringsressurser, læring og mestring, opplæring). To enhance consistency and replicability, a predefined set of search terms was applied across all websites where a search function permitted free-text entry. These terms included app, mobile application, self-management, rehabilitation, brain injury, spinal cord injury, cognitive, digital literacy, applied individually, and in combination. All search activities and the results retrieved from each website were recorded in a log, available in Multimedia Appendix 4. We searched the following selected organizational websites: CanChild [38], Child Neurology Foundation [39], HelseNorge [40], Kids Brain Health Network [41], LHL Stroke [42], Mayo Clinic [43], Motion Rehab Leeds [44], Sask Abilities [45], Shirley Ryan Ability Lab [46], The Learning Portal of the Southern and Eastern Norway Regional Health Authority [47], The Norwegian Childhood Cancer Society [48], The Norwegian National Advisory Unit on Learning and Mastery in Health [49], The Norwegian Personal Injury Association [50], The Norwegian Spinal Cord Injuries Association [51], and The Swiss Paraplegic Centre Nottwil [52].

#### Apps

We searched App Store across the Apple App Store (accessed via fnd.io, a third-party iOS app directory), and the Google Play Store, supplemented by a targeted search using the fnd.io aggregator. We conducted the searches using a list of condition-based search terms derived from the MeSH headings applied to the electronic database searches. These included, but were not limited to, concussion, head injury child, head injury adolescent, spina bifida, spina bifida cystica, meningomyelocele, SCI, spinal cord, hydrocephalus, CP, craniocerebral trauma, spinal cord neoplasms, and neurological disease youth (Multimedia Appendix 1; see Multimedia Appendix 5 for the full search term list).

### Selection of Sources of Evidence

All eligible abstracts were exported to Rayyan (Rayyan Systems Inc) software [53]. The research study reports were independently screened for inclusion by 2 reviewers (LM and JB). Conflicts were resolved by consensus and with the aid of a third reviewer (IE), if needed. Two reviewers independently carried out data extraction from the eligible studies (IE and EJS). The criteria used for screening and extracting are provided in our protocol [33].

The procedure for screening mobile apps and interactive e-learning websites was the same as for research studies. We found that available interactive e-learning websites always had an accompanying mobile app. We used the information provided in the app stores as our source for data extraction in these cases, and we will refer to these as mobile apps or apps for the remainder of this paper.

Mobile apps selected for full examination (equivalent to reading full-text for scientific sources) were downloaded via App Store or Google Play, and data were extracted from the information provided in the app stores. For some apps, additional information could be found in separate webpages provided by the developer or the affiliated organization behind the app. However, sometimes additional information was not available or not consistent. Furthermore, the information given in the stores is what is apparently available for the end user when he or she decides whether to download an app. For these reasons, we found App Store and Google Play to be the most relevant sources from which to extract information.

### Data Collection Process and Data Items

Before extracting data from the included sources, the author team had consensus meetings to decide the variables to be included in the summarization of evidence from the different sources. Discussions were based on previous experience in the author group and scoping reviews on similar topics. For the sources identified in App Store and Google Play, we followed recommendations and suggestions from Gastgeiger et al [54] specifically developed for data extraction from mobile apps. When we reached consensus, we developed 1 evidence table for data items for published reviews, 1 evidence table for data items for primary studies with 1 or more investigations, and 1 evidence table for data items for mobile apps. An overview of the selected data items is given in Multimedia Appendix 6. Detailed content was extracted for all variables from each included source and is available in 3 separate multimedia appendices: Multimedia Appendix 7 [55-57] for reviews, Multimedia Appendix 8 [58-62] for papers reporting interventions, and Multimedia Appendix 9 for mobile apps. The information in the appendices is presented without any edits or interpretations, in order not to change the meaning reported in the original sources of information, and to provide a comprehensive presentation of our findings. From the appendices, we then created summaries of the information we considered most relevant to answer the RQs. Critical appraisal of the included scientific sources was not performed, as our main aim was to map evidence in the field and not to assess study quality. Findings from the scientific sources address RQs 1 and 2, and findings from mobile apps address RQs 3 and 4. In the Results section, these key findings are presented graphically, in tables, and in complementary textual descriptions.

## Results

### Study Selection

The final database search identified 1211 abstracts for screening. After removing duplicates, 821 abstracts were screened in accordance with our screening criteria for titles and abstracts [33]. Two studies, both randomized controlled trials (RCTs), were eligible for data extraction [58,59]. The gray literature search resulted in 6 eligible publications, all found via Google Scholar: One RCT [60], 2 systematic reviews [55,56], 1 master’s thesis with an integrative literature review [57], 1 PhD thesis [61], and 1 doctor of nursing practice thesis [62]. In total, we identified 5 documents investigating 1 or more interventions and 3 review papers. Six of the 8 included publications were published from the United States [56,58-62], 1 from Finland [57], and the last from Australia [55]. Backward and forward citation screening was performed for all included publications but did not result in any additional sources eligible for inclusion. The searches for available mobile apps identified 114 apps for consideration. Of these, 84 were excluded, with wrong outcome or population as the most frequent reasons. Four apps were excluded as they had not been updated in the last 6 years, and 2 apps were excluded because they were no longer available in App Store or Google Play. The final number of eligible unique mobile apps was 30. A flowchart of the search results and record selection based on the search performed on June 16, 2026, is given in Figure 1.

**Figure 1.**
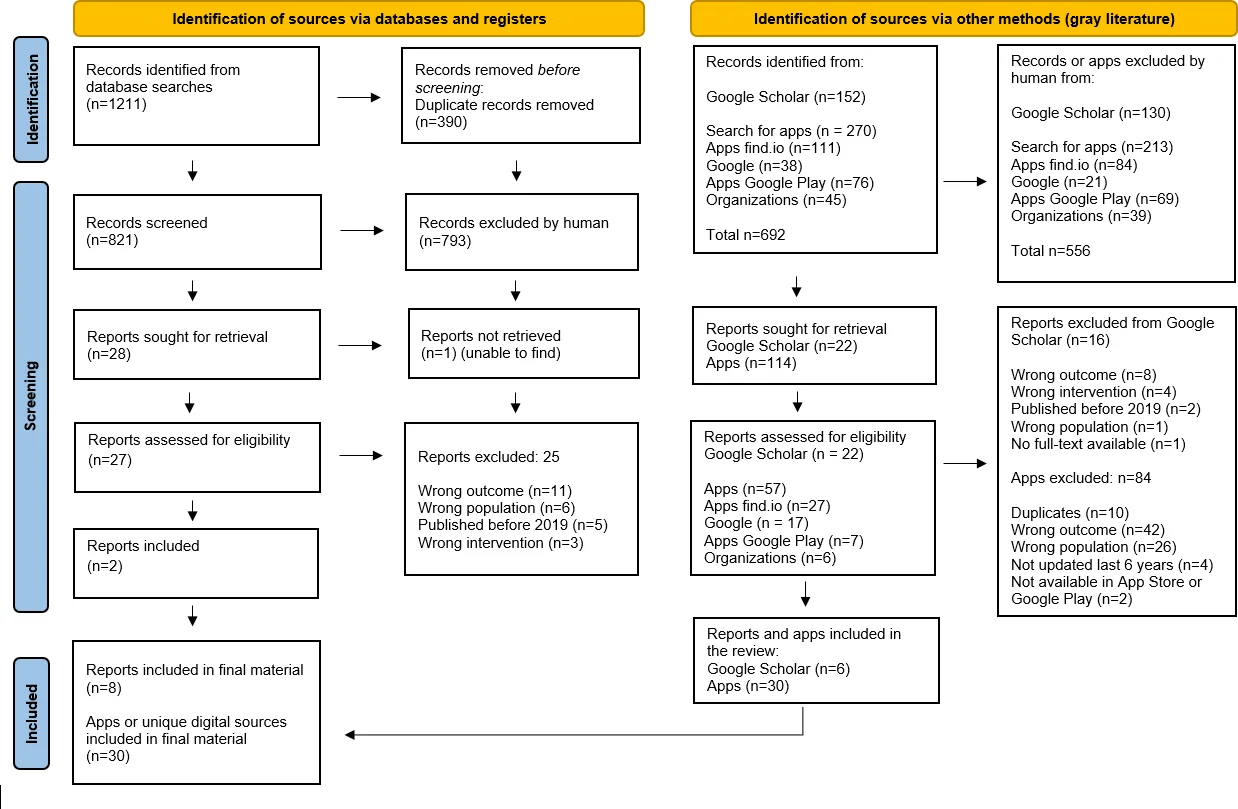
PRISMA (Preferred Reporting Items for Systematic Reviews and Meta-Analyses) 2020 flow diagram showing this study’s search results and record selection.

### What Evidence Exists on Our Topic, and What User Experiences Are Reported? (RQs 1 and 2)

An overview of the number of identified review papers, papers with 1 or more interventions, and mobile apps for brain injury or disease, SCI or disease, and both injuries and diseases from 2019 to 2026 is given in Figure 2. The total number of included sources reveals the sparsity of evidence on the topic for this scoping review and does not indicate an increase in published research. Of 30 apps, 20 have been updated in the last 2 years. Apps targeting brain injury or disease seem to be updated more frequently than the other 2 categories.

Evidence extracted from the review papers and the papers investigating 1 or more interventions addressing RQs 1 and 2 is given in Table 1. For detailed data, we refer to Multimedia Appendix 7 for review studies and Multimedia Appendix 8 for studies with 1 or more interventions. In Table 2, the study design versus target injury or disease is illustrated in a heat map.

**Figure 2.**
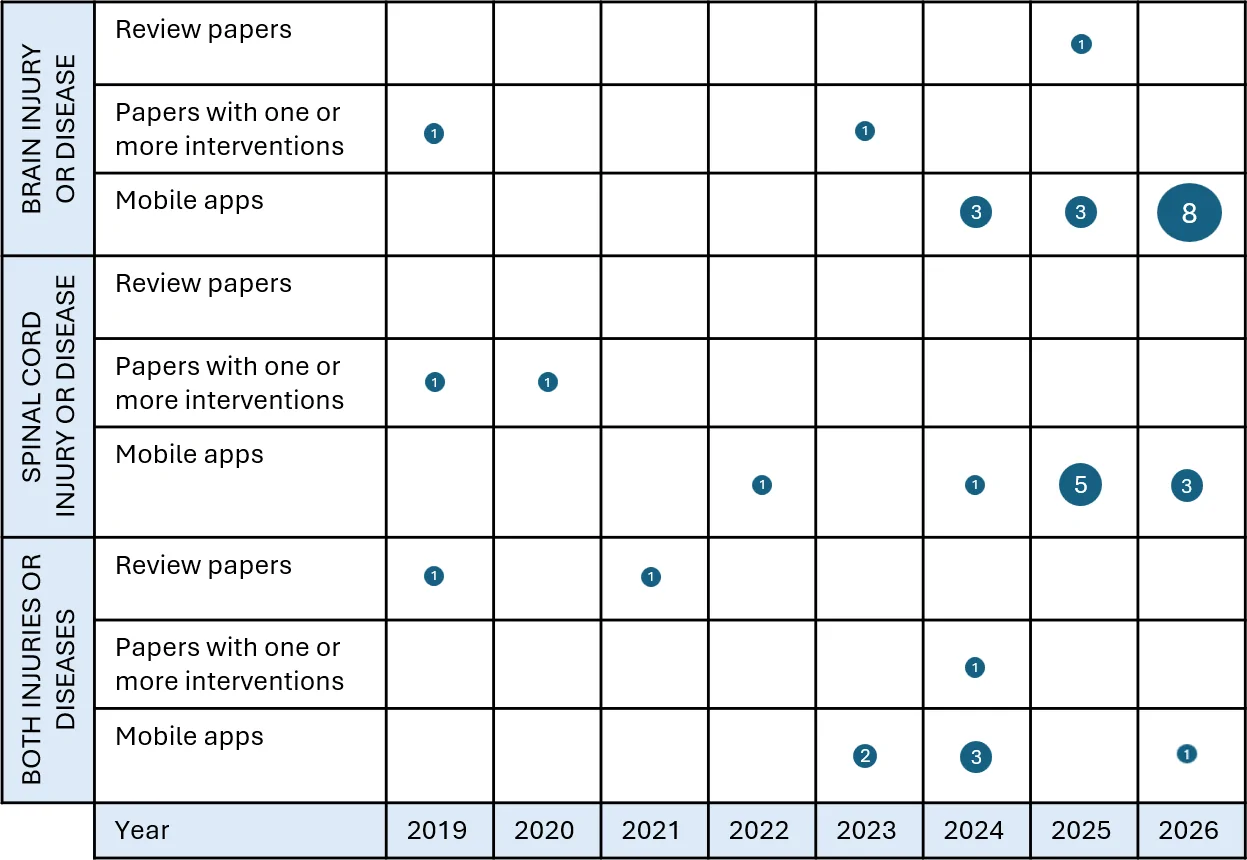
Overview of the number of identified review papers, papers with 1 or more interventions, and mobile apps for brain injury or disease, spinal cord injury or disease, and both injuries and diseases by year from 2019 to 2026.

**Table 1.** Main findings extracted from eligible documents (n=8, alphabetical order).

Author (year), study design, and target injury or disease	Aim	Main findings of relevance
Boulton et al (2025) [55], systematic review, brain injury or disease	To review available mobile apps and digital resources that are targeted at supporting the needs of children with developmental concerns or neurodevelopmental conditions	The lack of evidence across resources means that it is extremely difficult to provide recommendations to families with respect to apps or digital resources that may support their needs. The information quality of resources was mostly low. Apps and digital resources with good credibility and an existing evidence base were largely developed in partnership with research, health, or government institutions, and were rated significantly higher on overall quality.
Kryger et al (2019) [60], RCT^a^, spinal cord injury or disease	To determine whether the use of the iMHere^b^ system would be associated with improved health outcomes	The use of the iMHere mHealth^c^ platform resulted in a statistically significant reduction in UTIs^d^ over time, and a decrease in several other outcome measures, which trended toward, but did not reach, statistical significance.
Mitchell (2020) [62], cohort study, spinal cord injury or disease	To improve transition importance and transition confidence in medically complex adolescents with spina bifida and shunted hydrocephalus with usage of the intervention of HydroAssist Mobile App	An increase in transition importance and transition confidence scores was found with the use of the HydroAssist mobile app. The participants and their families found the app to be convenient, helpful, and informative, and users felt empowered to have their medical information on their phones.
Mäkinen (2021) [57], integrative review, both injuries and diseases	To examine the accessibility of mHealth apps for children and adolescents with neurological disabilities	The evidence-based knowledge about the use of mHealth app for children and adolescents with neurological disabilities is small, but the potential and need for efficient interventions are actual.
Nussbaum et al (2019) [56], systematic review, both injuries and diseases	To better define how mHealth apps have been used in environments relevant to physical medicine and rehabilitation	Of the 102 studies included, 42 were considered relevant for this scoping review as they addressed our target population. Of these, 1 app for SCI^e^, 2 apps for neurorehabilitation, and 1 app for TBI^f^ were evaluated relevant for assessments of digital health literacy.
Setiwan (2024) [61], multiple designs, both injuries and diseases	To develop, evaluate, and investigate the usefulness of an integrated and adaptable mHealth system, iMHere 2.0	The iMHere app was rated as highly usable, and the system was capable of addressing user needs and be adaptable to clinical workflows and settings.
Wade et al. (2019) [58], RCT, brain injury or disease	To examine changes in QoL^g^ in adolescents receiving family problem-solving therapy following traumatic brain injury	Online treatments were associated with clinical improvements, whereas the face-to-face treatment was not.
Wade et al (2023) [59], RCT, brain injury or disease	To examine the efficacy of the SMART (Self-Management After Recent Traumatic Brain Injury) program and potential moderators	The SMART program may be associated with more rapid recovery following mild TBI among youth with higher levels of problem-focused, engaged coping and resilience

a RCT: randomized controlled trial.

b iMHere: interactive Mobile Health and Rehabilitation.

c mHealth: mobile health.

d UTIs: urinary tract infections.

e SCI: spinal cord injury.

f TBI: traumatic brain injury.

g QoL: quality of life.

**Table 2.** Numbers of publications by study design for each target injury or disease.

Study design	Brain injury or disease, n	Spinal cord injury or disease, n	Both injuries and diseases, n	Sum
RCT^a^	2	1	0	3
Multiple	0	0	1	1
Cohort study	0	1	0	1
Integrative review	0	0	1	1
Systematic review	1	0	1	2
Sum	3	2	3	8

a RCT: randomized controlled trial.

We identified 2 systematic reviews [55,56] with 112 and 102 participants, respectively, and 1 integrative literature review [57] with 11 participants. Two of the reviews included individuals from both our target groups [56,57], and the third included individuals with brain injury [55]. The overall findings from these reviews emphasize that the evidence base for the use and efficacy of digital applications for our target population is sparse. The systematic review from Boulton et al [55] found that apps and digital sources developed in partnership with research, health, or government institutions scored higher on overall research quality and were more likely to be based on evidence than on sources developed without such partnerships.

The research studies investigating 1 or more interventions include 3 RCTs, 1 PhD thesis including multiple substudies with different study designs, and 1 DPN thesis with a cohort study design. Of the 3 RCTs, 2 investigated brain injury or disease [58,59] and were published by a single research group. The first of these RCTs [58] investigated differences in QoL in adolescents receiving family problem-solving therapy, either as face-to-face, self-guided online session, or therapist-guided online session. Family problem-solving therapy is a structured, skill-building intervention aiming to help families to communicate, manage emotions, and better solve daily problems. A total of 150 individuals with moderate to severe traumatic brain injury (TBI) and a mean age of 16.5 (SD 1.1) years were included, and the intervention lasted for 6 months. It was concluded that both online treatments, but not the face-to-face condition, were associated with clinical improvements assessed using the Pediatric Quality of Life Inventory and the Health and Behavior Inventory questionnaires. The second RCT from this group [59] compared outcomes from the Coping Strategies Inventory—Short Form, QoL, problem-focused engagement, and the Health and Behavior Inventory during a shorter intervention of 4 weeks involving 71 participants with mild TBI, randomized to either usual care or use of the SMART (Self-Management After Recent Traumatic Brain Injury) web app. The mean age of participants was 14.2 (SD 1.8) years. The authors concluded that the SMART program, which includes 8 interactive, multimedia psychoeducational modules partnered with a tool for symptom and daily activities tracking, was associated with more rapid recovery among those individuals who had higher levels of problem-focused coping and resilience. Furthermore, the need to tailor approaches to postconcussion management as well as further investigation of the SMART program itself was emphasized. The third RCT [60] investigated SCI and evaluated the effect of the interactive Mobile Health and Rehabilitation (iMHere) system. The system is an mHealth (mobile health) platform developed to support patients in managing their health independently. In the RCT, the iMHere system was compared with standard care in an intervention over 9 months with 38 participants with SCI with a mean age of 37.9 (SD 13.4) years. It must be noted that the population had a wide range of ages, including individuals within our defined age range as well as older adults. Outcome measures were health assessments from the number of urinary tract infections, pressure injuries, and number of emergency department visits and hospitalizations, as well as several questionnaires addressing psychological outcomes. Use of the iMHere system gave a significant reduction in urinary tract infections and a decrease in several other outcomes that trended, but were not statistically significant, in favor of the iMHere group.

The PhD thesis [61] comprised multiple substudies, addressing the development, piloting, and user testing of iMHere 2.0. Individuals with spina bifida, SCI, and CP were included, with the number of participants in substudies varying from 6 to 62, with an age range of 14 to 67 years. The iMHere app was rated as usable, and participants were positive about continuing to use it. Furthermore, the iMHere-system 2.0 was shown to be capable of addressing individual evolving needs, even in this heterogeneous group with regard to age and diagnosis. However, it was suggested that incorporating a self-training mechanism might improve patients’ understanding of the use of the app.

The final included study was a doctor of nursing practice thesis, investigating the HydroAssist mobile app for individuals with spina bifida in a cohort study [62]. HydroAssist is made to help patients manage their hydrocephalus symptoms by storing treatment histories, shunt settings, and brain scans. It enables tracking, storing, and sharing symptoms with health care providers. The intervention was provided for 2 weeks and included 7 participants with spina bifida with a mean age of 16.1 years. Transition importance and confidence scores with the use of the HydroAssist increased by 15% from pre- to posttest. The end users found the app to be convenient, helpful, informative, and empowering.

### Which Mobile Apps Are Available, and What Are Their Characteristics? (RQs3 and 4)

A mapping of the 30 included mobile apps with regard to affiliation with an organization, health personnel involvement, setting for use, cost, availability, recent updates, privacy policy, third-party authorizations, and electronic health record (EHR) integration sorted by target injury or disease (brain, spinal cord, or both) is presented in Figure 3. For detailed data, we refer to Multimedia Appendix 9.

Of 30 apps, 14 (47%) of the included apps targeted brain injury or disease or concussion, and 10 (33%) targeted SCI or disease, whereas 6 (20%) were addressing both injury and disease types. The intended minimum age ranged from 4 years to 18 years and older, indicating that many apps were designed for use by children, adolescents, and their caregivers.

Of 30 apps, 16 (53%) reported affiliation with a professional health organization, charity, academic institution, or government body, while the remaining apps were developed by private companies or independent developers without formal institutional affiliation. Apps targeting SCI or disease or both injury and disease types were more often associated with an organization (7/10, 70% and 4/6, 67%, respectively) than apps targeting brain injury or disease (5/14, 36%).

Health personnel were in many cases mentioned as involved in the development of apps, but explicit involvement of health professionals after the app was in use was required only for 12 (40%) of the apps. Of 30 apps, 6 (20%) stated in their information that they encourage data sharing with health professionals, carers, or family (Multimedia Appendix 9, source IDs 1, 6, 13, 14, 15, and 23), but without explaining the procedures enabling data sharing or export of data. Of 30 apps, 23 (77%) were designed primarily for home use supporting self or caregiver-assisted management. The remaining apps were intended for mixed use, combining home-based use with rehabilitation or clinical contexts.

Of 30 apps, 23 (77%) were free to use; 9 apps targeting SCI or disease, 9 targeting brain injury or disease, and 5 targeting both injuries and diseases. Four apps required subscription or one-time payment (Multimedia Appendix 9, source IDs 5, 6, 16, and 26), and 3 followed a freemium model (Multimedia Appendix 9, source IDs 8, 12, and 15), where basic features were available at no cost with paid upgrades for additional content or services. Five of the 7 apps requiring payment were targeting brain injury or disease.

Of 30 apps, 18 (60%) were supported both by iOS (App Store) and Android (Google Play), and 12 (40%) were available only in App Store. Of 30 apps, 5 (17%) combined mobile apps with web-based components (Multimedia Appendix 9, source IDs 6, 17, 20, 26, and 27). Regarding updates in the last 2 years, 78% (11/14) of the apps targeting brain injury or disease and 50% (5/10) of the apps targeting SCI or disease had been updated. None of the apps targeting both injuries and diseases had been updated.

All included apps referred to a developer privacy policy, indicating at least a basic level of transparency regarding data privacy and protection. To access further information, the user needs to follow the referenced link. Declarations of data collection varied considerably in the information provided in the app stores. Several tools reported that no personal data were collected, while others indicated collection of data such as health and fitness information, app usage data, or identifiers. Use of third-party authorizations was stated for 8 (27%) of the apps (Multimedia Appendix 9, source IDs 1, 7, 12, 15, 16, 20, 25, and 29). In all cases, it was specified which data were authorized for sharing. One app did not include any information on third-party authorizations (Multimedia Appendix 9, source ID 17).

Direct integration with EHRs was rare, with only 1 tool reporting full EHR integration (Multimedia Appendix 9, source ID 10) and 1 tool describing conditional sharing dependent on user action (Multimedia Appendix 9, source ID 4).

With regard to content, all apps clustered around a limited number of functional categories. The most common features were education and information provision, including explanations of injury, recovery processes, and self-management strategies. Symptom tracking and health diaries were also highly prevalent, enabling users to monitor symptoms, activities, or well-being over time.

**Figure 3.**
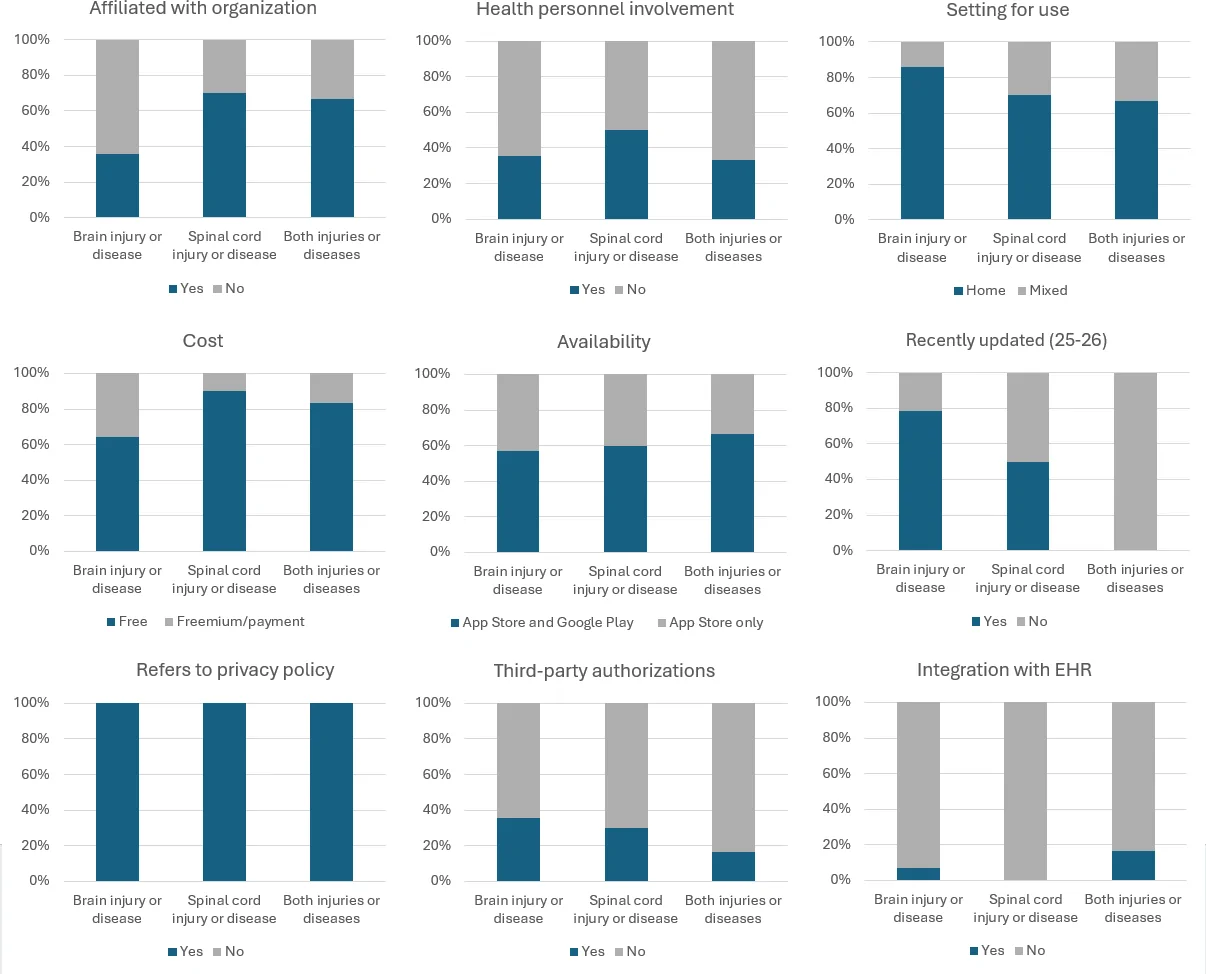
Characteristics of included mobile apps: distribution (percentage) of key variables is presented for included apps (n=30) to illustrate similarities and differences between apps targeting brain injury or disease, spinal cord injury or disease, or both injuries and diseases. EHR: electronic health record.

## Discussion

### Summary of Evidence

The sources we have identified in this scoping review show that the research evidence on the usefulness and efficacy of digital tools for enhancing health literacy in our target groups is sparse. In line with our findings, studies on the effectiveness of mHealth interventions addressing self-management and health care transition in adolescents and young adults with chronic conditions have previously shown to be very limited [31]. Also for healthy young individuals, evidence for usefulness, efficacy for digital tools on multiple health domains, and safety, including privacy or confidentiality concerns, is lacking [63]. It is unknown to us whether this is explained by a lack of research interest or whether the development in the field is so rapid that research is lagging behind. We identified very little reported user experience, and none of the included reviews focused specifically on this. Our findings further suggest that there are several privacy and security issues that may pose a challenge, both for end users’ ability to understand and navigate available apps and from a scientific point of view to make evaluations and recommendations.

Overall, the included mobile apps predominantly emphasized education, self-monitoring, and self-management. Specifically for the tools targeting brain injury or disease, a significant proportion of apps incorporated cognitive training, games, or assessments, while others offered structured rehabilitation or recovery plans, such as graded return-to-activity or stepwise rehabilitation protocols. Less frequently observed features included reminders and scheduling tools and peer or community support functionalities. The predominance of free, home-based tools suggests a strong focus on accessibility and user autonomy, which is important to diminish the digital divide that can arise due to demographic and socioeconomic differences [15].

### Identified Research Gaps and Future Knowledge Needs

#### Overview

Current literature is not consistent with regard to how digital health literacy is defined and understood [6]. In addition, the number of available mHealth apps at present exceeds 350,000 [64,65]. This extensive volume of apps, combined with multiple definitions and understandings of the concept of digital health literacy, made the search for and identification of relevant apps for this scoping review challenging and very time-consuming. Although several frameworks have been developed to guide clinicians and end users in selecting appropriate apps, this landscape is also characterized by considerable redundancy, as the literature references more than 130 different evaluation frameworks [64]. Existing frameworks further present varying criteria and methodologies for assessments, resulting in a lack of standard for evaluation [66]. Approaching the landscape of mobile apps and other digital tools from a scientific perspective is also challenging, as the search functions in app stores are limited, search results are not reproducible, and the information provided is not structured and amenable to easy screening using tools such as Covidence or Rayyan [54]. Instead, apps must be downloaded to extract information, and results may be biased due to factors such as user history, device type, location, and behavior.

Digital health literacy entails the ability of the end user to search, find, understand, assess, and apply information from the internet [3,7]. Mancone et al [30] point out that there is a lack of comprehensive models that encompass the full spectrum of health literacy, including digital health literacy. There are some specific outcome measures for digital health literacy described in the literature, such as the eHealth Literacy Scale [67], the Digital Health Literacy Instrument [7], the Digital Health Care Literacy Scale [68], and the Transactional eHealth Literacy Instrument [69]. Interestingly, none of the documents included in our final data material of published papers or theses used any of these outcome measures. Despite different definitions of the term and lack of consensus on evaluation due to multiple frameworks, digital health literacy is inevitably connected with a number of health outcomes, and how these are understood and managed by each individual. It may seem that research up until now does not incorporate digital health literacy as a main outcome, using the accompanying outcome measures available, but rather defines study outcomes from health- and self-reported variables. One of the main take-home messages from this scoping review is, nevertheless, that evidence for the usefulness and efficacy of mobile apps for improving self-management in our target population is meager.

Stauch et al [6] investigated digital health literacy among healthy children and adolescents and noted that interventions to improve digital health literacy are clearly needed, with regard to both privacy issues and navigation skills and the assessments of available health information. When evidence for the healthy population is sparse, there are limited possibilities to transfer knowledge to patient groups. A systematic review and meta-analysis by Verweel et al [70] investigated the effect of digital interventions on health literacy for adult individuals with chronic conditions. They concluded that evidence of effects of digital interventions on health literacy is limited, and further that there is an evident heterogeneity in study design, population, and outcomes. There are some studies concerning health literacy in adult individuals with SCI or brain injuries. Armstrong et al [71] investigated digital applications to promote the psychosocial health of adult individuals with SCI. The conclusions were that although this is an emerging field, published studies are not of consistent high quality, and interventions are not adapted to the end user needs. Also focused on adults, Silva et al [72] stated that studies on health literacy in SCI are limited, although this population is in need of continuous guidance to make decisions and act on self-care due to the complexity and individual variations of their condition. Their review, however, addressed health literacy in general, not specifically digital health literacy. Finally, 1 review on digital health interventions in TBI rehabilitation and caregiver education [73] concluded that despite some reports showing positive outcomes exist, there is a need for improved methodological rigor, with more precise descriptions of interventions and more consistent outcome measurements.

One reason for the sparsity of published research could be that development of a health mobile app needs funding and then additional funding for evaluation, both to assess efficacy and to meet regulatory demands [74]. It is well established that conducting a trial for evidence of efficacy from the gold standard of an RCT-design is very resource intensive [75], and for smaller companies, this may not be a possible option [74]. A recent review from Showell et al [76] further found the time from trial completion to publication for RCTs to be on average 2.1 years. It is therefore not surprising that several apps that are funded, developed, and evaluated from a scientific approach never become available beyond the clinical trial [77]. In our scoping review, the iMHere system (Multimedia Appendix 9, source ID 10), affiliated with a university, appears to exemplify this challenge. Parmanto et al [78] published a paper presenting the development and implementation of this (then) novel system already in 2013. Two of our included intervention studies investigated iMHere, with an RCT from 2019 [60] and a PhD thesis from 2024 comprising multiple substudies with different designs [61]. From the website information given by the developer [79], the development of the system is accompanied by an ongoing RCT to evaluate outcomes such as self-management-skills, secondary conditions, and costs, but results are not available on the efficacy of these outcomes. The latest publication found from the project is from 2023, when a qualitative study with thematic analyses of focus group feedback was published [80]. At present, only the caregiver version of the app is available to download for all in the App Store and Google Play. End users need to register via the website to get the app, which is not commercially available. An interesting question emerges: is a scientific approach to the development and testing of new apps for small, targeted user groups sustainable—or can it be too rigorous? For management of specific medical procedures such as catheterization or monitoring of a shunt, it can obviously be argued that tailored development is required and also manageable—exemplified with some of the apps included in our material such as the CathRmdr (Multimedia Appendix 9, source ID 3), Kid’s guide: using a catheter (Multimedia Appendix 9, source ID 11), HC&ME (Multimedia Appendix 9, source ID 7), and HydroAssist (Multimedia Appendix 9, source ID 9). But many of the challenges individuals with injuries to the spinal cord or brain face in their daily life are of a more generic nature, such as pain, fatigue, cognitive challenges, structure and planning, and training exercises. If a digital tool can help them understand and manage their symptoms better, this can increase digital health literacy, even if the tool they use is not specifically developed for their condition. In the frame of evidence-based practice [81], this emphasizes the importance of always integrating patient values and preferences when considering digital interventions or recommending an app. By focusing on symptom control rather than specific diseases, one may be able to develop more user-friendly apps subjected to more comprehensive testing.

A possible approach for more rapid development of evidence both enabling health personnel to give recommendations and end users to find appropriate digital tools could be to concentrate future research on the usefulness and efficacy of established and widely used commercially available apps, instead of developing time-consuming and costly small-scale apps. Many different neurological diseases have common psychological and functional challenges; thus, it could be beneficial for scalability, scope, and cost to develop cross-diagnostic digital interventions [82]. Stiles-Shields et al [83] investigated the usability of a well-established existing weight management app for youth with spina bifida and concluded that the approach to adapt existing solutions and personalize them for the needs of a specific population can be an alternative to the development of new tools. Specifically for SCI, it has been suggested that many features of relevance are common with chronic diseases, and that development of digital tools for this specific patient population can build on the architecture of already existing apps, making the development efficient and the outcome usable for several populations [26]. On this matter, it is also an interesting question whether generative artificial intelligence and chatbots may replace the use of apps in their present form. Models for collaborations between industry and academic research groups are suggested as a topic for future research in this field to strengthen the integration of evidence into the development process of the companies involved [74].

User experiences with the HydroAssist app and iMHere system (Multimedia Appendix 9, source IDs 9 and 10) are addressed in the theses from Mithcell [62] and Setiwan [61], respectively. They both report that users stated these 2 specific tools to meet their needs, and they found them convenient to use. For the HydroAssist app, it was further specifically mentioned that users felt empowered to have their medical information available on their phone. The digital transformation in health care alters the role of the end users, who instead of being passive receivers of information from health care providers may have the opportunity to more actively engage in self-management of their condition [84]. Mäkinen [57] mentions in a narrative review the potential for mHealth apps to support self-management and health empowerment. The 2 RCTs included in this scoping review from Wade et al [58,59] also emphasize the need for user-specific digital tools, especially as heterogeneity in user needs requires individualized adjustments. To include user experiences in the development and evaluation of new digital solutions is thus essential.

For end users to be able to use information from digital tools to manage their health condition, they must have social, technical, critical, and analytic skills [85]. The ability to translate health knowledge acquired from digital sources into relevant actions is fundamental for realizing increased autonomy in end users [84]. For the apps available both at the App Store and Google Play, we found that the information was not always consistent between the stores. Sometimes, we also found discrepancies between information given on separate webpages from the developer or affiliated organization. For example, for one of the apps we included, the accompanying webpage states that “data can be easily shared with health providers.” In the App Store, however, it is stated that “data are not recorded.” Consequently, it is likely to be difficult for the users to make sense of this information. Regarding privacy and security, the App Store and Google Play list which data are stored and “may be shared.” All apps included in this review further refer to the developers’ privacy policy through a link to an external webpage. The information provided on these webpages is, however, in most cases 6 to 8 pages of quite complex language, with legal information and paragraphs, which are difficult both to read and understand. This can represent a challenge for the user’s ability to understand which data are collected, and whether and in what way they are stored. It seems like a paradox that an app developed to enhance understanding and management of an individual's health condition does not provide essential information on what data are collected, how they are stored, and whether they are shared with a third party. If understanding the privacy consequences to download an app requires more digital literacy than using the actual app, it may potentially increase the digital divide among skilled and less skilled users of digital tools, consequently also potentially increasing health disparities [16]. Setiwan [61], included in this review, proposes that low retention rates in the use of the iMHere system may suggest that users do not fully comprehend how to use the app. As apps intended for autonomous use in most cases will not include any training or instructions, they suggest incorporating a self-training feature within the app to improve patients’ understanding.

The apparent lack of integration with EHRs, as well as variable data practice transparency, highlights ongoing gaps in interoperability and standardization of mHealth apps. This, combined with the fact that many health apps are considered wellness tools outside the scope of health care regulation and consequently are not evaluated prior to launch, makes it difficult to evaluate privacy management, security, and efficiency from a scientific point of view [64,86]. Few peer-reviewed studies exist to confirm promoted health benefits of health apps; study quality is often low, and existing evaluation studies are not standardized [87]. This diversity in the assessments is often accompanied by a lack of theoretical models, making both comparison of evaluations and recommendations for apps challenging [88]. The majority of app developers do not conduct or publish any studies, and the studies that do exist are characterized by methodological weaknesses [65]. Stricter requirements apply to digital health applications classified as medical devices, including rigorous testing and validation [89]. While these regulations aim to improve safety and quality, they also increase the complexity of the regulatory landscape. For end users, it may be challenging to identify whether an app is a regulated medical device or is intended for general wellness purposes only. This adds an additional layer of difficulty when navigating and selecting safe and appropriate digital health tools.

#### Availability and Characteristics of Apps

Although we identified 30 unique mobile apps, this number is small compared with the abundant number of available health apps. Two of the apps we identified were investigated in published papers we included with 1 or more interventions (Table 1): the HydroAssist app (Multimedia Appendix 9, source ID 9) [62] and the iMHere system (Multimedia Appendix 9, source ID 10) [60,61]. The low number of included apps, combined with both our own limited number of included scientific sources and the knowledge gaps mentioned in the literature on the field, suggests that general health apps are very dominant compared with diagnosis- and age-specific apps. A systematic review from 2019 by Christopher et al [90] concluded that the number of mobile apps for TBI was low, with a dominant focus on sports-related concussion. It was further noted that the evidence for their usefulness for the specific target group with TBI was sparse. Our findings suggest that this still is a valid statement. An interesting point in the review from Boulton et al [55] that we included in this scoping review was that apps developed in partnership with research, health, or government institutions were rated higher on overall quality and supported by stronger evidence than apps not developed in such partnerships. Our findings revealed that half of the apps included were affiliated with a professional medical or health association, charity, or government body. Nine apps stated that data could be shared with health personnel, and 7 apps were stated to have an intended use both at home and at an institution. Thus, the majority of apps are intended to be used independently from home. This aligns with other studies concerning general health apps, stating that the majority of existing apps are free to use and targeted toward the end users, but they also lack evidence for their claimed effects [65,74,91]. During our screening process, we discovered several apps with high recommendation scores and a large number of users that did not meet our eligibility criteria because they were not developed and targeted toward specific patient groups or diagnoses. Still, they included many similar goals and features as our included apps. Among established apps relevant for individuals with head injury, featuring brain-training games, puzzles, and problem-solving, which appears highly relevant, Elevate, Peak, Bearable, MindDoc, and Headspace are examples of frequently recommended apps. Of a more generic nature, focusing on pain, stress management, and organization of daily activities, Manage My Pain and TinyBit can be mentioned. We have not been able to find any studies that have evaluated these or other general health apps for use for our specific target groups. However, there is reason to believe that our target users may benefit from the use of such apps, as exemplified previously in this discussion. An apparent advantage of well-established apps is that they are easily available for end users, usually without cost (premium versions may occur), and that they seem to be updated quite frequently. Mumcu [91] showed that the majority of existing health and fitness apps are free and also that this clearly corresponds to the number of downloads compared with apps with an end user cost. Free use thus seems to be an important user preference. In our review process, we excluded 6 apps that were first considered eligible for inclusion but were no longer available in any app stores or not updated in the past 5 years (Figure 1). Furthermore, among sources that were included, several apps appear to not have been updated in the last 2 years (exemplified from Multimedia Appendix 9, source IDs 10, 11, 13, 14, 18, 19, 21, 24, and 28). This suggests that sustainable maintenance of small, specifically developed apps for targeted user groups is challenging. Intended user age was identified as another issue of concern. Mariani et al [92] highlight an apparent need for clearer distinctions between age groups and stricter privacy regulations for apps targeting younger users. The age given in App Store and Google Play for our included apps seems to be based on whether the content of each app is regarded as age appropriate, rather than whether the content can be understood by the user. For many of the included apps, it seems highly unlikely that a child of 4 years will be able to use the app or understand any of the features, although “4+” is stated in the app stores. Thus, the recommended age given in App Store and Google Play cannot be regarded as meaningful guidance of age target groups for each application.

### Strengths and Limitations of the Scoping Review

This scoping review is based on a thorough mapping of available published studies, reports, and theses through systematic searches in multiple databases, searches in gray literature, and an extensive assessment of mobile apps available on the App Store and Google Play. Two of our included studies, Kryger et al [60] and Setiwan [61], included individuals older than 25 years. Thus, results from these sources are not based only on adolescents or young adults, and results must therefore be interpreted with some caution. We still consider it relevant to include these sources, as the overall evidence in the field is very sparse, and findings also reflect on the younger population. The extremely rapid development in the field of digital apps and resources poses a challenge. Unlike database searches, search results from app stores are not entirely reproducible across users or devices. We did not take screenshots or other forms of documentation to archive the search results, which we in hindsight see could have been useful. We identified apps during our screening process that were no longer available when we started data extraction from the app stores. Some apps had changed names or were not available in the languages the original screening suggested they would be. The probability that new apps have been launched while this manuscript is being processed is present. Due to the rapid development in this field, it is also unlikely that all apps we have included in our material are still available. Finally, the search for gray literature included 15 selected websites, but extracting data from these sources faced some challenges due to the inconsistent quality and, in some cases, the absence of effective search engines on these sites. This process could have been strengthened by incorporating input and recommendations from experts in the field.

### Conclusions

This scoping review provides insight into available digital tools for improving health literacy in a target population of young individuals with injury or disease to the brain and/or spinal cord. We found that published evidence for this topic is sparse. Only 8 publications and 30 apps met eligibility criteria. Compared with the abundant number of available general health and wellness apps, few tailored digital tools with active end user involvement exist. Available apps emphasize education, symptom tracking, and self-management but lack evidence for their claimed effects. Furthermore, privacy information is often difficult to interpret, both for end users and from a scientific perspective. We pose the question of whether understanding an app’s privacy policy actually may require greater digital literacy than using the app itself. More rigorous, user-centered evaluation of effective digital tools is therefore needed to identify evidence-based and safe apps for our target population.

## Supplementary material

10.2196/101750 Multimedia Appendix 1 Searches in scientific databases.

10.2196/101750 Multimedia Appendix 2 Summary of gray literature searches.

10.2196/101750 Multimedia Appendix 3 Searches in Google and Google Scholar.

10.2196/101750 Multimedia Appendix 4 Searches in organizational websites.

10.2196/101750 Multimedia Appendix 5 Searches for mobile apps.

10.2196/101750 Multimedia Appendix 6 Selected data items.

10.2196/101750 Multimedia Appendix 7 Extracted data from review papers.

10.2196/101750 Multimedia Appendix 8 Extracted data from papers with 1 or more interventions.

10.2196/101750 Multimedia Appendix 9 Extracted data from included mobile apps.

10.2196/101750 Checklist 1 PRISMA-ScR checklist.

## Abbreviations

CP: cerebral palsy
EHR: electronic health record
iMHere: interactive Mobile Health and Rehabilitation)
mHealth: mobile health
PRISMA: Preferred Reporting Items for Systematic Reviews and Meta-Analyses
PRISMA-ScR: Preferred Reporting Items for Systematic Reviews and Meta-Analyses extension for Scoping Reviews
QoL: quality of life
RCT: randomized controlled trial
RQ: research question
SCI: spinal cord injury
SMART: Self-Management After Recent Traumatic brain injury
TBI: traumatic brain injury

## References

[1] Whitehead L, Talevski J, Fatehi F, Beauchamp A (2023). Barriers to and facilitators of digital health among culturally and linguistically diverse populations: qualitative systematic review. J Med Internet Res.

[2] Backman C, Papp R, Tonjock Kolle A (2024). Platform-based patient-clinician digital health interventions for care transitions: scoping review. J Med Internet Res.

[3] Fitzpatrick PJ (2023). Improving health literacy using the power of digital communications to achieve better health outcomes for patients and practitioners. Front Digit Health.

[4] Van de Velde D, De Zutter F, Satink T (2019). Delineating the concept of self-management in chronic conditions: a concept analysis. BMJ Open.

[5] (2024). Health literacy. World Health Organization.

[6] Stauch L, Renninger D, Rangnow P (2025). Digital health literacy of children and adolescents and its association with sociodemographic factors: representative study findings from Germany. J Med Internet Res.

[7] van der Vaart R, Drossaert C (2017). Development of the digital health literacy instrument: measuring a broad spectrum of Health 1.0 and Health 2.0 skills. J Med Internet Res.

[8] Emerson MR, Buckland S, Lawlor MA (2022). Addressing and evaluating health literacy in mHealth: a scoping review. Mhealth.

[9] Faux-Nightingale A, Philp F, Chadwick D, Singh B, Pandyan A (2022). tools to evaluate digital health literacy and engagement with eHealth resources: a scoping review. Heliyon.

[10] Estrela M, Semedo G, Roque F, Ferreira PL, Herdeiro MT (2023). Sociodemographic determinants of digital health literacy: a systematic review and meta-analysis. Int J Med Inform.

[11] Yuen E, Winter N, Savira F (2024). Digital health literacy and its association with sociodemographic characteristics, health resource use, and health outcomes: rapid review. Interact J Med Res.

[12] Busse TS, Nitsche J, Kernebeck S (2022). Approaches to improvement of Digital Health Literacy (eHL) in the context of person-centered care. Int J Environ Res Public Health.

[13] Yang K, Hu Y, Qi H (2022). Digital health literacy: bibliometric analysis. J Med Internet Res.

[14] Arias López MDP, Ong BA, Borrat Frigola X (2023). Digital literacy as a new determinant of health: a scoping review. PLOS Digit Health.

[15] Gallegos-Rejas VM, Thomas EE, Kelly JT, Smith AC (2023). A multi-stakeholder approach is needed to reduce the digital divide and encourage equitable access to telehealth. J Telemed Telecare.

[16] Adedinsewo D, Eberly L, Sokumbi O, Rodriguez JA, Patten CA, Brewer LC (2023). Health disparities, clinical trials, and the digital divide. Mayo Clin Proc.

[17] Hochstetler A, Raskin J, Blazer-Yost BL (2022). Hydrocephalus: historical analysis and considerations for treatment. Eur J Med Res.

[18] Paul S, Nahar A, Bhagawati M, Kunwar AJ (2022). A review on recent advances of cerebral palsy. Oxid Med Cell Longev.

[19] Sattoe JNT, Hilberink SR (2023). Impairments and comorbidities in adults with cerebral palsy and spina bifida: a meta-analysis. Front Neurol.

[20] Vitrikas K, Dalton H, Breish D (2020). Cerebral palsy: an overview. Am Fam Physician.

[21] Causio FA, Gandolfi S, Kaur J, Sert B, Fakhfakh M, Angelis LD (2025). Impact of digital health interventions on health literacy: a systematic review with quality appraisal. medRxiv.

[22] Tan Y, Liang X, Ming W (2025). The efficacy of telemedicine interventions on quality of life and depression in individuals with spinal cord injury: a systematic review and meta-analysis. Front Psychiatry.

[23] Zaghloul H, Fanous K, Ahmed L (2025). Digital health literacy in patients with common chronic diseases: systematic review and meta-analysis. J Med Internet Res.

[24] Miranda JPP (2024). Mobile Applications in Advancing Health Literacy: A Review of Methodologies, Participants, and Challenges.

[25] Mahmoudi E, Yejong Yoo P, Chandra A (2024). Gamification in mobile apps for children with disabilities: scoping review. JMIR Serious Games.

[26] Bernard RM, Seijas V, Davis M (2024). Self-management support apps for spinal cord injury: results of a systematic search in app stores and mobile app rating scale evaluation. JMIR Mhealth Uhealth.

[27] Mota A, Ferreira MC, Fernandes CS (2026). Mobile health applications for the rehabilitation of people with spinal cord injury: a scoping review. Disabil Rehabil Assist Technol.

[28] Cha SM (2024). Mobile application applied for cognitive rehabilitation: a systematic review. Life (Basel).

[29] Cao W, Kadir AA, Tang W, Wang J, Yuan J, Hassan II (2024). Effectiveness of mobile application interventions for stroke survivors: systematic review and meta-analysis. BMC Med Inform Decis Mak.

[30] Mancone S, Corrado S, Tosti B, Spica G, Diotaiuti P (2024). Integrating digital and interactive approaches in adolescent health literacy: a comprehensive review. Front Public Health.

[31] Li Z, Lu F, Wu J (2024). Usability and effectiveness of eHealth and mHealth interventions that support self-management and health care transition in adolescents and young adults with chronic disease: systematic review. J Med Internet Res.

[32] Niltakan K (2024). Health literacy in the digital age: challenges and opportunities. JBER.

[33] (2026). Digital health literacy in children and adolescents with brain or spinal cord disorders: a scoping review protocol. Open Science Framework.

[34] Peters MDJ, Marnie C, Tricco AC (2020). Updated methodological guidance for the conduct of scoping reviews. JBI Evid Synth.

[35] Tricco AC, Lillie E, Zarin W (2018). PRISMA extension for scoping reviews (PRISMA-ScR): checklist and explanation. Ann Intern Med.

[36] Google Scholar.

[37] Google.

[38] CanChild. CanChild.

[39] Child Neurology Foundation.

[40] HelseNorge.

[41] Kids Brain Health Network.

[42] LHL Stroke.

[43] Mayo Clinic.

[44] Motion Rehab Leeds.

[45] Sask Abilities.

[46] Shirley Ryan Ability Lab.

[47] The learning portal of the southern and eastern Norway regional health authority. HELSE.

[48] The Norwegian Childhood Cancer Society.

[49] The Norwegian National Advisory Unit for Learning and Mastery in Health.

[50] The Norwegian Personal Injury Association.

[51] The Norwegian Spinal Cord Injuries Association.

[52] The Swiss Paraplegic Centre Nottwil.

[53] Rayyan.

[54] Gasteiger N, Dowding D, Norman G (2023). Conducting a systematic review and evaluation of commercially available mobile applications (apps) on a health-related topic: the TECH approach and a step-by-step methodological guide. BMJ Open.

[55] Boulton KA, Hilton M, Sutton E, Guastella AJ (2025). Apps and digital resources for child neurodevelopment, mental health, and well-being: review, evaluation, and reflection on current resources. J Med Internet Res.

[56] Nussbaum R, Kelly C, Quinby E, Mac A, Parmanto B, Dicianno BE (2019). Systematic review of mobile health applications in rehabilitation. Arch Phys Med Rehabil.

[57] Mäkinen L (2021). MHealth Applications for Children and Adolescents With Neurological Disabilities [Master’s thesis]. https://www.theseus.fi/server/api/core/bitstreams/d1843365-4e84-41ea-8087-7e607935649b/content.

[58] Wade SL, Cassedy AE, Taylor HG (2019). Adolescent quality of life following family problem-solving treatment for brain injury. J Consult Clin Psychol.

[59] Wade SL, Sidol C, Babcock L (2023). Findings from a randomized controlled trial of SMART: An EHealth intervention for mild traumatic brain injury. J Pediatr Psychol.

[60] Kryger MA, Crytzer TM, Fairman A (2019). The effect of the interactive mobile health and rehabilitation system on health and psychosocial outcomes in spinal cord injury: randomized controlled trial. J Med Internet Res.

[61] Setiwan IMA (2024). An integrated and adaptable mhealth system to support individuals with chronic conditions and disabilities: development, evaluation, and exploration for future works. https://d-scholarship.pitt.edu/concern/etds/ebfeab58-5e9b-414e-bd23-c5b8840d5f50?parent_query=Setiawan%20PhD%202024&highlight=true.

[62] Mitchell L (2020). Enhance transition care for medically complex adolescents. https://hdl.handle.net/10355/73324.

[63] Stewart E, Milton A, Yee HF (2022). eHealth tools that assess and track health and well-being in children and young people: systematic review. J Med Internet Res.

[64] Herpertz J, Dwyer B, Taylor J, Opel N, Torous J (2025). Developing a standardized framework for evaluating health apps using natural language processing. Sci Rep.

[65] Morley J, Laitila J, Ross JS, Schamroth J, Zhang J, Floridi L (2025). An app a day will (probably not) keep the doctor away: an evidence audit of health and medical apps available on the apple app store. Minds Mach.

[66] Galavi Z, Norouzi S, Khajouei R (2024). Heuristics used for evaluating the usability of mobile health applications: a systematic literature review. Digit Health.

[67] Norman CD, Skinner HA (2006). eHEALS: the eHealth Literacy Scale. J Med Internet Res.

[68] Nelson LA, Pennings JS, Sommer EC, Popescu F, Barkin SL (2022). A 3-item measure of digital health care literacy: development and validation study. JMIR Form Res.

[69] Paige SR, Stellefson M, Krieger JL, Miller MD, Cheong J, Anderson-Lewis C (2019). Transactional eHealth literacy: developing and testing a multi-dimensional instrument. J Health Commun.

[70] Verweel L, Newman A, Michaelchuk W, Packham T, Goldstein R, Brooks D (2023). The effect of digital interventions on related health literacy and skills for individuals living with chronic diseases: a systematic review and meta-analysis. Int J Med Inform.

[71] Armstrong A, Oetinger K, Weimer K, Hönig K (2024). Digital psychosocial interventions for individuals with spinal cord injury: a scoping review. Front Psychiatry.

[72] Silva FAR, Barbosa MA, Prudente COM (2023). Health literacy of people with spinal cord injury: a systematic review. Spinal Cord.

[73] Avramovic P, Rietdijk R, Attard M, Kenny B, Power E, Togher L (2023). Cognitive and behavioral digital health interventions for people with traumatic brain injury and their caregivers: a systematic review. J Neurotrauma.

[74] Prentice C, Peven K, Zhaunova L (2024). Methods for evaluating the efficacy and effectiveness of direct-to-consumer mobile health apps: a scoping review. BMC Digit Health.

[75] Hariton E, Locascio JJ (2018). Randomised controlled trials—the gold standard for effectiveness research: study design: randomised controlled trials. BJOG.

[76] Showell MG, Cole S, Clarke MJ, DeVito NJ, Farquhar C, Jordan V (2024). Time to publication for results of clinical trials. Cochrane Database Syst Rev.

[77] DeMatteo C, Jakubowski J, Stazyk K, Randall S, Perrotta S, Zhang R (2024). The headaches of developing a concussion app for youth. Int J E-Health Med Commun.

[78] Parmanto B, Pramana G, Yu DX, Fairman AD, Dicianno BE, McCue MP (2013). iMHere: a novel mHealth system for supporting self-care in management of complex and chronic conditions. JMIR Mhealth Uhealth.

[79] iMHere. iMHere.

[80] Hu H, Ambadar Z, Quinby E (2023). The iMHere 2.0 system for family caregivers of older adults: a focus group. Int J Telerehabil.

[81] Dusin J, Melanson A, Mische-Lawson L (2023). Evidence-based practice models and frameworks in the healthcare setting: a scoping review. BMJ Open.

[82] Keevers Y, Sivaneswaran A, Dudeney J, Woldhuis T, Gandy M (2026). Digital psychological interventions in youth with neurological disorders: a systematic review. J Pediatr Psychol.

[83] Stiles-Shields C, Garcia B, Villota K, Wartman E, Winning AM, Holmbeck GN (2019). Exploring an existing weight management app for use with adolescents and young adults with spina bifida: usability study. JMIR Pediatr Parent.

[84] Ban S, Kim Y, Seomun G (2024). Digital health literacy: a concept analysis. Digit Health.

[85] Levin-Zamir D, Van den Broucke S, Bíró É (2025). Measuring digital health literacy and its associations with determinants and health outcomes in 13 countries. Front Public Health.

[86] Mescher T, Hacker RL, Martinez LA, Morris CD, Mishkind MC, Garver-Apgar CE (2025). Mobile health apps: guidance for evaluation and implementation by healthcare workers. J Technol Behav Sci.

[87] Alon N, Torous J (2023). Current challenges for evaluating mobile health applications. J Am Med Inform Assoc.

[88] Hach S, Alder G, Stavric V, Taylor D, Signal N (2024). Usability assessment methods for mobile apps for physical rehabilitation: umbrella review. JMIR Mhealth Uhealth.

[89] Gomis-Pastor M, Berdún J, Borrás-Santos A (2024). Clinical validation of digital healthcare solutions: state of the art, challenges and opportunities. Health Care (Don Mills).

[90] Christopher E, Alsaffarini KW, Jamjoom AA (2019). Mobile health for traumatic brain injury: a systematic review of the literature and mobile application market. Cureus.

[91] Mumcu D (2025). Post-pandemic dynamics of mobile health and fitness applications: an analysis of user profiles, market developments, and emerging trends. MRAJ.

[92] Mariani A, Liberato M, Sperotto A, Affinito A Analyzing privacy implications of mobile apps data collection across age groups.

