# Supplementary material for: Mobile Apps to Enhance Health Literacy in Children and Adolescents With Injury or Disease to the Brain or Spinal Cord: Scoping Review
Source: JMIR Pediatr Parent. 2026 Nov 13;9:e101750. doi: 10.2196/101750 (PMC13762682; doi:10.2196/101750)
Supplement: Supplementary file 2 [file pediatrics-v9-e101750-s001.docx]

**Multimedia Appendix 1. Searches in scientific databases.**

**Documentation of the literature search for: Mobile applications to enhance health literacy in children and adolescents with injury or disease to the brain or spinal cord: A scoping review**

The following databases were searched:

| **Database** | **Number of retrieved references** |
| --- | --- |
| MEDLINE (Ovid) | 164 |
| Embase (Ovid) | 309 |
| APA PsycInfo (Ovid) | 81 |
| AMED (Ovid) | 3 |
| Eric (Ovid) | 7 |
| Cochrane Database of Systematic Reviews (CDSR, Cochrane Library) | 6 |
| Cochrane Protocols (Cochrane Library) | 0 |
| Trials (Cochrane Library) | 36 |
| CINAHL (EBSCO) | 300 |
| Scopus (Elsevier) | 305 |
|  |  |
| Number of references before deduplication: | 1211 |
| Number of references after deduplication: | 821 |

All searches were performed on 16.06.2026 by academic librarian Hilde Strømme, University of Oslo Library.

**MEDLINE**

Ovid MEDLINE(R) ALL <1946 to June 15, 2026>

| 1 | exp Craniocerebral Trauma/ or exp Spinal Cord Injuries/ or exp Hydrocephalus/ or Cerebral Palsy/ or Meningomyelocele/ or Spina Bifida Cystica/ or Spinal Cord Neoplasms/ | 320030 |
| --- | --- | --- |
| 2 | (((head or crani* or cerebr* or capitis or brain* or forebrain* or skull* or hemispher* or intracran* or intercran* or encephalopath*) adj3 (injur* or trauma* or damag* or wound* or fracture* or contusion*)) or (("spinal cord" adj3 (injur* or contusion or lacerat* or transec* or trauma* or ischemia or myelopath*)) or (paraplegi* or quadriplegi* or tetraplegi*)) or (hydrocephal* or hydroencephal* or ventriculomegal* or (ventri* adj1 (enlarge* or dialat*)) or aqueductal stenos* or ((cerebrospinal fluid or cerebro spinal fluid) adj1 accumulat*) or ((intraventricular or extraventricular) adj2 obstruct*)) or (cerebral adj pals*) or cp or (meningomyelocele* or myelomeningocele* or myelocele*) or ("spina bifida" adj1 (aperta or manifest* or open or cystic)) or ("spinal cord" adj3 (tumo* or neoplasm*))).tw,kw,kf. | 459272 |
| 3 | 1 or 2 | 594199 |
| 4 | Mobile Applications/ or exp Cell Phone/ or exp Computers, Handheld/ or Telerehabilitation/ or exp Internet/ or exp Video Games/ or Gamification/ | 162654 |
| 5 | (Mhealth or web* or telerehabilitation or interactive or mobile or smartphone* or (smart adj phone*) or cellphone* or (cell* adj phone*) or tablet* or handheld or ipad* or iphone* or android or app* or web* or internet or device* or ("e-learning" or "e-education" or ((electronic or on-line or online) adj3 (educat* or learn* or train* or course*))) or (((digital or mobile or electronic or computer* or video or app*) adj3 (game or games)) or gaming or gamification)).tw,kw,kf. | 10615028 |
| 6 | 4 or 5 | 10654812 |
| 7 | Health Literacy/ | 13637 |
| 8 | (((digital or ehealth or e-health or "electronic health" or "internet health" or mhealth or m-health) adj3 litera*) or (health* adj3 (litera* or decision* or competenc* or knowledg* or behavio*)) or ("eHealth Literacy Scale" or eHEALS or "Digital Health Literacy Instrument" or DHLI or "Digital Health Literacy Scale" or DHLS or "Transactional eHealth Literacy Instrument" or TeHLI)).tw,kw,kf. | 205900 |
| 9 | 7 or 8 | 208512 |
| 10 | exp child/ or adolescent/ or Young Adult/ | 4233107 |
| 11 | (child* or adolesc* or teen* or youth or minor or minors or juvenile or paediatric* or pediatric* or (young adj3 (people or person* or boy* or girl* or adult* or man or men or woman or women))).tw,kw,kf. | 2998622 |
| 12 | 10 or 11 | 5428487 |
| 13 | 3 and 6 and 9 and 12 | 268 |
| 14 | limit 13 to yr="2019 - 2026" | 164 |

**Embase**

Embase Classic+Embase <1947 to 2026 June 12>

| 1 | exp head injury/ or exp spinal cord injury/ or exp hydrocephalus/ or cerebral palsy/ or meningomyelocele/ or spinal dysraphism/ or exp spinal cord tumor/ | 641810 |
| --- | --- | --- |
| 2 | (((head or crani* or cerebr* or capitis or brain* or forebrain* or skull* or hemispher* or intracran* or intercran* or encephalopath*) adj3 (injur* or trauma* or damag* or wound* or fracture* or contusion*)) or (("spinal cord" adj3 (injur* or contusion or lacerat* or transec* or trauma* or ischemia or myelopath*)) or (paraplegi* or quadriplegi* or tetraplegi*)) or (hydrocephal* or hydroencephal* or ventriculomegal* or (ventri* adj1 (enlarge* or dialat*)) or aqueductal stenos* or ((cerebrospinal fluid or cerebro spinal fluid) adj1 accumulat*) or ((intraventricular or extraventricular) adj2 obstruct*)) or (cerebral adj pals*) or cp or (meningomyelocele* or myelomeningocele* or myelocele*) or ("spina bifida" adj1 (aperta or manifest* or open or cystic)) or ("spinal cord" adj3 (tumo* or neoplasm*))).tw,kw,kf. | 653374 |
| 3 | 1 or 2 | 951913 |
| 4 | exp mobile application/ or exp mobile phone/ or personal digital assistant/ or tablet computer/ or Telerehabilitation/ or exp Internet/ or e-learning/ or exp video game/ or gamification/ | 274561 |
| 5 | (Mhealth or web* or telerehabilitation or interactive or mobile or smartphone* or (smart adj phone*) or cellphone* or (cell* adj phone*) or tablet* or handheld or ipad* or iphone* or android or app* or web* or internet or device* or ("e-learning" or "e-education" or ((electronic or on-line or online) adj3 (educat* or learn* or train* or course*))) or (((digital or mobile or electronic or computer* or video or app*) adj3 (game or games)) or gaming or gamification)).tw,kw,kf. | 13840181 |
| 6 | 4 or 5 | 13891515 |
| 7 | exp Health Literacy/ | 33518 |
| 8 | (((digital or ehealth or e-health or "electronic health" or "internet health" or mhealth or m-health) adj3 litera*) or (health* adj3 (litera* or decision* or competenc* or knowledg* or behavio*)) or ("eHealth Literacy Scale" or eHEALS or "Digital Health Literacy Instrument" or DHLI or "Digital Health Literacy Scale" or DHLS or "Transactional eHealth Literacy Instrument" or TeHLI)).tw,kw,kf. | 250286 |
| 9 | 7 or 8 | 258298 |
| 10 | exp child/ or exp adolescent/ or young adult/ | 5611602 |
| 11 | (child* or adolesc* or teen* or youth or minor or minors or juvenile or paediatric* or pediatric* or (young adj3 (people or person* or boy* or girl* or adult* or man or men or woman or women))).tw,kw,kf. | 4195691 |
| 12 | 10 or 11 | 7021061 |
| 13 | 3 and 6 and 9 and 12 | 511 |
| 14 | limit 13 to yr="2019 - 2026" | 309 |

**APA PsycInfo**

APA PsycInfo <1987 to June 2026 Week 2>

| 1 | exp Brain Injuries/ or exp Spinal Cord Injuries/ or Hydrocephalus/ or Cerebral Palsy/ or Spina Bifida/ | 43117 |
| --- | --- | --- |
| 2 | (((head or crani* or cerebr* or capitis or brain* or forebrain* or skull* or hemispher* or intracran* or intercran* or encephalopath*) adj3 (injur* or trauma* or damag* or wound* or fracture* or contusion*)) or (("spinal cord" adj3 (injur* or contusion or lacerat* or transec* or trauma* or ischemia or myelopath*)) or (paraplegi* or quadriplegi* or tetraplegi*)) or (hydrocephal* or hydroencephal* or ventriculomegal* or (ventri* adj1 (enlarge* or dialat*)) or aqueductal stenos* or ((cerebrospinal fluid or cerebro spinal fluid) adj1 accumulat*) or ((intraventricular or extraventricular) adj2 obstruct*)) or (cerebral adj pals*) or cp or (meningomyelocele* or myelomeningocele* or myelocele*) or ("spina bifida" adj1 (aperta or manifest* or open or cystic)) or ("spinal cord" adj3 (tumo* or neoplasm*))).tw. | 79023 |
| 3 | 1 or 2 | 82448 |
| 4 | exp Mobile Applications/ or exp Mobile Phones/ or "Smartphone Use"/ or Tablet Computers/ or exp Internet/ or Telerehabilitation/ or Digital Gaming/ or Computer Applications/ or exp Computer Games/ or Gamification/ | 74894 |
| 5 | (Mhealth or web* or telerehabilitation or interactive or mobile or smartphone* or (smart adj phone*) or cellphone* or (cell* adj phone*) or tablet* or handheld or ipad* or iphone* or android or app* or web* or internet or device* or ("e-learning" or "e-education" or ((electronic or on-line or online) adj3 (educat* or learn* or train* or course*))) or (((digital or mobile or electronic or computer* or video or app*) adj3 (game or games)) or gaming or gamification)).tw. | 1807025 |
| 6 | 4 or 5 | 1815214 |
| 7 | exp Health Literacy/ | 6111 |
| 8 | (((digital or ehealth or e-health or "electronic health" or "internet health" or mhealth or m-health) adj3 litera*) or (health* adj3 (litera* or decision* or competenc* or knowledg* or behavio*)) or ("eHealth Literacy Scale" or eHEALS or "Digital Health Literacy Instrument" or DHLI or "Digital Health Literacy Scale" or DHLS or "Transactional eHealth Literacy Instrument" or TeHLI)).tw. | 95863 |
| 9 | 7 or 8 | 96618 |
| 10 | ("100" or "200" or "320").ag. | 1406759 |
| 11 | (child* or adolesc* or teen* or youth or minor or minors or juvenile or paediatric* or pediatric* or (young adj3 (people or person* or boy* or girl* or adult* or man or men or woman or women))).tw. | 1059801 |
| 12 | 10 or 11 | 1773574 |
| 13 | 3 and 6 and 9 and 12 | 160 |
| 14 | limit 13 to yr="2019 - 2026" | 81 |

**AMED**

AMED (Allied and Complementary Medicine) <1985 to April 2026>

| 1 | exp Head Injuries/ or exp Spinal Cord Injuries/ or Hydrocephalus/ or Cerebral Palsy/ or Meningomyelocele/ | 15116 |
| --- | --- | --- |
| 2 | (((head or crani* or cerebr* or capitis or brain* or forebrain* or skull* or hemispher* or intracran* or intercran* or encephalopath*) adj3 (injur* or trauma* or damag* or wound* or fracture* or contusion*)) or (("spinal cord" adj3 (injur* or contusion or lacerat* or transec* or trauma* or ischemia or myelopath*)) or (paraplegi* or quadriplegi* or tetraplegi*)) or (hydrocephal* or hydroencephal* or ventriculomegal* or (ventri* adj1 (enlarge* or dialat*)) or aqueductal stenos* or ((cerebrospinal fluid or cerebro spinal fluid) adj1 accumulat*) or ((intraventricular or extraventricular) adj2 obstruct*)) or (cerebral adj pals*) or cp or (meningomyelocele* or myelomeningocele* or myelocele*) or ("spina bifida" adj1 (aperta or manifest* or open or cystic)) or ("spinal cord" adj3 (tumo* or neoplasm*))).ti,ab,et. | 16357 |
| 3 | 1 or 2 | 18500 |
| 4 | Internet/ | 1456 |
| 5 | (Mhealth or web* or telerehabilitation or interactive or mobile or smartphone* or (smart adj phone*) or cellphone* or (cell* adj phone*) or tablet* or handheld or ipad* or iphone* or android or app* or web* or internet or device* or ("e-learning" or "e-education" or ((electronic or on-line or online) adj3 (educat* or learn* or train* or course*))) or (((digital or mobile or electronic or computer* or video or app*) adj3 (game or games)) or gaming or gamification)).ti,ab,et. | 73730 |
| 6 | 4 or 5 | 74180 |
| 7 | (((digital or ehealth or e-health or "electronic health" or "internet health" or mhealth or m-health) adj3 litera*) or (health* adj3 (litera* or decision* or competenc* or knowledg* or behavio*)) or ("eHealth Literacy Scale" or eHEALS or "Digital Health Literacy Instrument" or DHLI or "Digital Health Literacy Scale" or DHLS or "Transactional eHealth Literacy Instrument" or TeHLI)).ti,ab,et. | 2648 |
| 8 | exp child/ or exp Adolescent/ | 24009 |
| 9 | (child* or adolesc* or teen* or youth or minor or minors or juvenile or paediatric* or pediatric* or (young adj3 (people or person* or boy* or girl* or adult* or man or men or woman or women))).ti,ab,et. | 31121 |
| 10 | 8 or 9 | 36139 |
| 11 | 3 and 6 and 7 and 10 | 9 |
| 12 | limit 11 to yr="2019 - 2026" | 3 |

**ERIC**

ERIC <1965 to May 2026>

| 1 | Head Injuries/ or cerebral palsy/ | 2248 |
| --- | --- | --- |
| 2 | (((head or crani* or cerebr* or capitis or brain* or forebrain* or skull* or hemispher* or intracran* or intercran* or encephalopath*) adj3 (injur* or trauma* or damag* or wound* or fracture* or contusion*)) or (("spinal cord" adj3 (injur* or contusion or lacerat* or transec* or trauma* or ischemia or myelopath*)) or (paraplegi* or quadriplegi* or tetraplegi*)) or (hydrocephal* or hydroencephal* or ventriculomegal* or (ventri* adj1 (enlarge* or dialat*)) or aqueductal stenos* or ((cerebrospinal fluid or cerebro spinal fluid) adj1 accumulat*) or ((intraventricular or extraventricular) adj2 obstruct*)) or (cerebral adj pals*) or cp or (meningomyelocele* or myelomeningocele* or myelocele*) or ("spina bifida" adj1 (aperta or manifest* or open or cystic)) or ("spinal cord" adj3 (tumo* or neoplasm*))).tw. | 6902 |
| 3 | 1 or 2 | 6902 |
| 4 | exp Handheld Devices/ or internet/ or Video Games/ or Gamification/ | 37970 |
| 5 | (Mhealth or web* or telerehabilitation or interactive or mobile or smartphone* or (smart adj phone*) or cellphone* or (cell* adj phone*) or tablet* or handheld or ipad* or iphone* or android or app* or web* or internet or device* or ("e-learning" or "e-education" or ((electronic or on-line or online) adj3 (educat* or learn* or train* or course*))) or (((digital or mobile or electronic or computer* or video or app*) adj3 (game or games)) or gaming or gamification)).tw. | 862732 |
| 6 | 4 or 5 | 862732 |
| 7 | Digital Literacy/ | 1825 |
| 8 | (((digital or ehealth or e-health or "electronic health" or "internet health" or mhealth or m-health) adj3 litera*) or (health* adj3 (litera* or decision* or competenc* or knowledg* or behavio*)) or ("eHealth Literacy Scale" or eHEALS or "Digital Health Literacy Instrument" or DHLI or "Digital Health Literacy Scale" or DHLS or "Transactional eHealth Literacy Instrument" or TeHLI)).tw. | 18578 |
| 9 | 7 or 8 | 18578 |
| 10 | exp children/ or adolescents/ or Late Adolescents/ or exp Youth/ or Young Adults/ | 208454 |
| 11 | (child* or adolesc* or teen* or youth or minor or minors or juvenile or paediatric* or pediatric* or (young adj3 (people or person* or boy* or girl* or adult* or man or men or woman or women))).tw. | 497622 |
| 12 | 10 or 11 | 502050 |
| 13 | 3 and 6 and 9 and 12 | 12 |
| 14 | limit 13 to yr="2019 - 2026" | 7 |

**Cochrane Library**

| #1 | ([mh "Craniocerebral Trauma"] OR [mh "Spinal Cord Injuries"] OR [mh "Hydrocephalus"] OR [mh ^"Cerebral Palsy"] OR [mh ^"Meningomyelocele"] OR [mh ^"Spina Bifida Cystica"] OR [mh ^"Spinal Cord Neoplasms"]) | 11654 |
| --- | --- | --- |
| #2 | (((head OR crani* OR cerebr* OR capitis OR brain* OR forebrain* OR skull* OR hemispher* OR intracran* OR intercran* OR encephalopath*) NEAR/3 (injur* OR trauma* OR damag* OR wound* OR fracture* OR contusion*)) OR ((("spinal cord") NEAR/3 (injur* OR contusion or lacerat* or transec* or trauma* or ischemia OR myelopath*)) OR (paraplegi* or quadriplegi* OR tetraplegi*)) OR (hydrocephal* OR hydroencephal* OR ventriculomegal* OR (ventri* NEAR/1 (enlarge* OR dialat*)) OR (aqueductal NEXT stenos*) OR (((cerebrospinal NEXT fluid) OR (cerebro NEXT spinal NEXT fluid)) NEAR/1 accumulat*) OR ((intraventricular OR extraventricular) NEAR/2 obstruct*)) OR (cerebral NEXT pals*) OR cp OR (meningomyelocele* OR myelomeningocele* OR myelocele*) OR ("spina bifida" NEAR/1 (aperta OR manifest* OR open OR cystic)) OR ("spinal cord" NEAR/3 (tumo* OR neoplasm*))):ti,ab,kw | 34446 |
| #3 | #1 OR #2 | 36111 |
| #4 | ([mh ^"Mobile Applications"] OR [mh "Cell Phone"] OR [mh "Computers, Handheld"] OR [mh ^"Telerehabilitation"] OR [mh "Internet"] OR [mh "Video Games"] OR [mh ^"Gamification"]) | 15272 |
| #5 | (Mhealth OR web* OR telerehabilitation OR interactive OR mobile OR smartphone* OR (smart NEXT phone*) OR cellphone* OR (cell* NEXT phone*) OR tablet* OR handheld OR ipad* OR iphone* OR android OR app* OR web* OR internet OR device* OR ("e-learning" OR "e-education" OR ((electronic OR on-line OR online) NEAR/3 (educat* OR learn* OR train* OR course*))) OR (((digital OR mobile OR electronic OR computer* OR video OR app*) NEAR/3 (game OR games)) OR gaming OR gamification)):ti,ab,kw | 756619 |
| #6 | #4 OR #5 | 757354 |
| #7 | [mh ^"Health Literacy"] | 864 |
| #8 | (((digital OR ehealth OR "e-health" OR "electronic health" OR "internet health" OR mhealth OR "m-health") NEAR/3 litera*) OR (health* NEAR/3 (litera* OR decision* OR competenc* OR knowledg* OR behavio*)) OR ("eHealth Literacy Scale" or eHEALS or "Digital Health Literacy Instrument" or DHLI or "Digital Health Literacy Scale" or DHLS or "Transactional eHealth Literacy Instrument" or TeHLI)):ti,ab,kw | 37139 |
| #9 | #7 OR #8 | 37139 |
| #10 | ([mh "child"] OR [mh ^"adolescent"] OR [mh ^"young adult"]) | 242645 |
| #11 | (child* or adolesc* or teen* or youth or minor or minors or juvenile or paediatric* or pediatric* or (young NEAR/2 (people or person* or boy* or girl* or adult* or man or men or woman or women))):ti,ab,kw | 458427 |
| #12 | #10 OR #11 | 458427 |
| #13 | #3 AND #6 AND #9 AND #12 | 76 |
| #14 | #3 AND #6 AND #9 AND #12 with Cochrane Library publication date Between Jan 2019 and Jun 2026, in Cochrane Reviews, Cochrane Protocols | 6 |
| #15 | #3 AND #6 AND #9 AND #12 with Publication Year from 2019 to 2026, in Trials | 36 |

**CINAHL**

| **#** | **Query** | **Results** |
| --- | --- | --- |
| S1 | ((MH "Head Injuries+") OR (MH "Spinal Cord Injuries+") OR (MH "Hydrocephalus+") OR (MH "Cerebral Palsy") OR (MH "Spina Bifida") OR (MH "Spinal Cord Neoplasms")) | 97,387 |
| S2 | TX (((head OR crani* OR cerebr* OR capitis OR brain* OR forebrain* OR skull* OR hemispher* OR intracran* OR intercran* OR encephalopath*) N2 (injur* OR trauma* OR damag* OR wound* OR fracture* OR contusion*)) OR ((("spinal cord") N2 (injur* OR contusion or lacerat* or transec* or trauma* or ischemia OR myelopath*)) OR (paraplegi* or quadriplegi* OR tetraplegi*)) OR (hydrocephal* OR hydroencephal* OR ventriculomegal* OR (ventri* N0 (enlarge* OR dialat*)) OR aqueductal stenos* OR ((cerebrospinal fluid OR cerebro spinal fluid) N0 accumulat*) OR ((intraventricular OR extraventricular) N1 obstruct*)) OR (cerebral N0 pals*) OR cp OR (meningomyelocele* OR myelomeningocele* OR myelocele*) OR ("spina bifida" N0 (aperta OR manifest* OR open OR cystic)) OR ("spinal cord" N2 (tumo* OR neoplasm*))) | 146,534 |
| S3 | S1 OR S2 | 161,094 |
| S4 | ((MH "Mobile Applications") OR (MH "Cellular Phone") OR (MH "Smartphone") OR (MH "Computers, Hand-Held") OR (MH "Telerehabilitation") OR (MH "Internet+") OER (MH "Video Games+") OR (MH "Gamification")) | 31,225 |
| S5 | TX (Mhealth OR web* OR telerehabilitation OR interactive OR mobile OR smartphone* OR (smart N0 phone*) OR cellphone* OR (cell* N0 phone*) OR tablet* OR handheld OR ipad* OR iphone* OR android OR app* OR web* OR internet OR device* OR ("e-learning" OR "e-education" OR ((electronic OR on-line OR online) N2 (educat* OR learn* OR train* OR course*))) OR (((digital OR mobile OR electronic OR computer* OR video OR app*) N2 (game OR games)) OR gaming OR gamification)) | 2,131,620 |
| S6 | S4 OR S5 | 2,133,032 |
| S7 | (MH "Health Literacy") | 11,721 |
| S8 | TX (((digital OR ehealth OR e-health OR "electronic health" OR "internet health" OR mhealth OR m-health) N2 litera*) OR (health* N2 (litera* OR decision* OR competenc* OR knowledg* OR behavio*)) OR ("eHealth Literacy Scale" or eHEALS or "Digital Health Literacy Instrument" or DHLI or "Digital Health Literacy Scale" or DHLS or "Transactional eHealth Literacy Instrument" or TeHLI)) | 239,527 |
| S9 | S7 OR S8 | 239,527 |
| S10 | ((MH "Child+") OR (MH "Adolescence+") OR (MH "Young Adult")) | 1,335,444 |
| S11 | TX (child* or adolesc* or teen* or youth or minor or minors or juvenile or paediatric* or pediatric* or (young N2 (people or person* or boy* or girl* or adult* or man or men or woman or women))) | 2,024,914 |
| S12 | S10 OR S11 | 2,028,126 |
| S13 | S3 AND S6 AND S9 AND S12 | 717 |
| S14 | S3 AND S6 AND S9 AND S12  Publication Date: 01/01/2019 - 06/3/2026 | 300 |

**Scopus**

TITLE-ABS-KEY ((((head OR crani* OR cerebr* OR capitis OR brain* OR forebrain* OR skull* OR hemispher* OR intracran* OR intercran* OR encephalopath*) W/2 (injur* OR trauma* OR damag* OR wound* OR fracture* OR contusion*)) OR ((("spinal cord") W/2 (injur* OR contusion OR lacerat* OR transec* OR trauma* OR ischemia OR myelopath*)) OR (paraplegi* OR quadriplegi* OR tetraplegi*)) OR (hydrocephal* OR hydroencephal* OR ventriculomegal* OR (ventri* W/0 (enlarge* OR dialat*)) OR (aqueductal W/0 stenos*) OR (("cerebrospinal fluid" OR "cerebro spinal fluid") W/0 accumulat*) OR ((intraventricular OR extraventricular) W/1 obstruct*)) OR (cerebral W/0 pals*) OR cp OR (meningomyelocele* OR myelomeningocele* OR myelocele*) OR ("spina bifida" W/0 (aperta OR manifest* OR open OR cystic)) OR ("spinal cord" W/2 (tumo* OR neoplasm*))) AND (mhealth OR web* OR telerehabilitation OR interactive OR mobile OR smartphone* OR (smart W/0 phone*) OR cellphone* OR (cell* W/0 phone*) OR tablet* OR handheld OR ipad* OR iphone* OR android OR app* OR web* OR internet OR device* OR ("e-learning" OR "e-education" OR ((electronic OR on-line OR online) W/3 (educat* OR learn* OR train* OR course*))) OR (((digital OR mobile OR electronic OR computer* OR video OR app*) W/3 (game OR games)) OR gaming OR gamification)) AND (((digital OR ehealth OR "e-health" OR "electronic health" OR "internet health" OR mhealth OR "m-health") W/3 litera*) OR (health* W/3 (litera* OR decision* OR competenc* OR knowledg* OR behavio*)) OR ("eHealth Literacy Scale" OR eHEALS OR "Digital Health Literacy Instrument" OR DHLI OR "Digital Health Literacy Scale" OR DHLS OR "Transactional eHealth Literacy Instrument" OR TeHLI)) AND (child* OR adolesc* OR teen* OR youth OR minor OR minors OR juvenile OR paediatric* OR pediatric* OR (young W/2 (people OR person* OR boy* OR girl* OR adult* OR man OR men OR woman OR women)))) AND PUBYEAR > 2018 AND PUBYEAR < 2026

305 hits
