# Supplementary material for: Mobile Apps to Enhance Health Literacy in Children and Adolescents With Injury or Disease to the Brain or Spinal Cord: Scoping Review
Source: JMIR Pediatr Parent. 2026 Nov 13;9:e101750. doi: 10.2196/101750 (PMC13762682; doi:10.2196/101750)
Supplement: Supplementary file 3 [file pediatrics-v9-e101750-s002.docx]

**Multimedia Appendix 2. Summary of gray literature searches (January to April 2025).**

| **Source** | **String(s) used** | **Results screened** | **Archiving method** |
| --- | --- | --- | --- |
| Google Scholar | Strings 1–6 (see Appendix 3) | First 10 pages | URL^a^+ title logged at point of research |
| Google | Condition-specific simplified strings (see Appendix 3) | First 20 results (2 pages) per query | URL + title logged |
| Organisational websites | Internal search or site: operator (see Appendix 4) | All returned results | Page URLs logged with date of access |
| Fnd.io | Condition-specific terms (see Appendix 5) | All returned results | Full table logged; zero results recorded |
| Google Play | Condition-specific terms (see Appendix 5) | All returned results | Full table logged; |
| Apple App Store | Condition-specific terms (see Appendix 5) | All returned results | Full table logged; |
| ^a^ URL: Uniform Resource Locator | | | |
