# Supplementary material for: Mobile Apps to Enhance Health Literacy in Children and Adolescents With Injury or Disease to the Brain or Spinal Cord: Scoping Review
Source: JMIR Pediatr Parent. 2026 Nov 13;9:e101750. doi: 10.2196/101750 (PMC13762682; doi:10.2196/101750)
Supplement: Supplementary file 4 [file pediatrics-v9-e101750-s003.docx]

**Multimedia Appendix 3. Searches in Google and Google Scholar.**

We conducted a gray literature search using the search engines Google and Google Scholar and relevant international organizations for children/youth with injury to the brain or spinal cord. The initial search string for Google scholar was (child* OR adolescen* OR youth OR teen*) AND (Mhealth or web* or telerehabilitation or interactive or mobile or smartphone*OR cell phone OR mobile applications) AND (Craniocerebral Trauma OR Spinal Cord Injuries OR Hydrocephalus OR Cerebral Palsy OR Meningomyelocele OR Spina Bifida Cystica OR Spinal Cord Neoplasms). As only 7 hits were retrieved by using the full string, we decided to modify the string (child* OR adolescen* OR youth OR teen*) AND (web* or interactive or mobile or smartphone*OR cell phone OR mobile applications) AND (brain trauma OR Spinal Cord Injuries OR Hydrocephalus OR Cerebral Palsy OR Meningomyelocele OR Spina Bifida Cystica OR Spinal Cord Neoplasms)

Google strings were simplified to “apps about [disease OR disease] and [children OR youth]. “

## Google Scholar Summary

String1: (child* OR adolescen* OR youth OR teen*) AND (Mhealth or web* or telerehabilitation or interactive or mobile or smartphone*OR cell phone OR mobile applications) AND (Craniocerebral Trauma OR Spinal Cord Injuries OR Hydrocephalus OR Cerebral Palsy OR Meningomyelocele OR Spina Bifida Cystica OR Spinal Cord Neoplasms).

String 2: (child* OR adolescen* OR youth OR teen*) AND (web* or interactive or mobile or smartphone*OR cell phone OR mobile applications) AND (brain trauma OR Spinal Cord Injuries OR Hydrocephalus OR Cerebral Palsy OR Meningomyelocele OR Spina Bifida Cystica OR Spinal Cord Neoplasms).

String 3: (child* OR adolescen* OR youth OR teen*) AND (web* OR 'interactive web pages' OR 'mobile applications' OR mobile tool* OR digital OR telehealth OR gaming ) AND ('health literacy OR 'patient education') AND (brain trauma OR brain injury OR 'spinal cord injury' OR Hydrocephalus OR Cerebral Palsy OR Spina Bifida Cystica).

String 4: (child* OR adolescen* OR youth OR teen*) AND (web* OR 'mobile applications' OR mobile OR digital OR telehealth OR gaming ) AND ('health literacy' OR 'patient education') AND (brain trauma OR brain injury OR 'spinal cord injury' OR Hydrocephalus OR Cerebral Palsy OR Spina Bifida).

String 5: (Web app OR App OR digital app OR game OR digital) AND (child* or youth) AND (cerebral palsy OR brain disease OR neurolog*).

| **NO** | **Title** | **Link** |
| --- | --- | --- |
| 1 | Remote follow-up technologies in traumatic brain injury: a scoping review | [https://www.liebertpub.com/doi/pdf/10.1089/neu.2022.0138](https://protect.checkpoint.com/v2/r02/___https:/www.liebertpub.com/doi/pdf/10.1089/neu.2022.0138___.YzJlOmRpcmVrdG9yYXRmb3JtZWRpc2luc2tlcHJvZHVrdGVyOmM6bzplZDNmY2I5NTQ2MjExMTQ4YjVmMDQ0NjBkZDM5OTUwYzo3Ojk0MDM6ZjU0YzZjNzc0MTUwYTlmZTJkMTQ2NjNhNGVkODlhMGRlZGRkYjMwODM3YTQ2NDMxM2Q3NDY1MmVkOTk5YzUyZjpwOlQ6Rg) |
| 2 | The impact of interactive telerehabilitiation on people with physical disabilities | [https://vb.lsu.lt/object/elaba:165573482/](https://protect.checkpoint.com/v2/r02/___https:/vb.lsu.lt/object/elaba:165573482/___.YzJlOmRpcmVrdG9yYXRmb3JtZWRpc2luc2tlcHJvZHVrdGVyOmM6bzplZDNmY2I5NTQ2MjExMTQ4YjVmMDQ0NjBkZDM5OTUwYzo3OjQxNjg6MmNkNzA4OThiZjM4OGI3OTNhMDQwZDk5YTUxNzY4NGI5NzBlNjRmNzU0YTU3ZDJkYjBlZWMyMjQ3ZTZmYjZhNzpwOlQ6Rg) |
| 3 | Systematic review of telemedicine in spine surgery | [https://thejns.org/spine/view/journals/j-neurosurg-spine/34/2/article-p161.xml](https://protect.checkpoint.com/v2/r02/___https:/thejns.org/spine/view/journals/j-neurosurg-spine/34/2/article-p161.xml___.YzJlOmRpcmVrdG9yYXRmb3JtZWRpc2luc2tlcHJvZHVrdGVyOmM6bzplZDNmY2I5NTQ2MjExMTQ4YjVmMDQ0NjBkZDM5OTUwYzo3OmMzYzE6MTNjNjk2NGNiYjgxOGJiNGNmYzZlNDk5NzMwOTczODA1OTY4OTZhMzE4NDU1NTJmMTE4NzIxNWZlNTIzZjYyMTpwOlQ6Rg) |
| 4 | Physical Medicine & Rehabilitation Secrets: Physical Medicine & Rehabilitation Secrets | [https://books.google.no/books?hl=en&lr=&id=frqjEAAAQBAJ&oi=fnd&pg=PP1&dq=++(child*+OR+adolescen*+OR+youth+OR+teen*)+AND+(Mhealth+or+web*+or+telerehabilitation+or+interactive+or+mobile+or+smartphone*OR+cell+phone+OR+mobile+applications)+AND+(Craniocerebral+Trauma+OR+Spinal+Cord+Injuries+OR+Hydrocephalus+OR+Cerebral+Palsy+OR+Meningomyelocele+OR+Spina+Bifida+Cystica+OR+Spinal+Cord+Neoplasms)+&ots=FatGX2Gjn_&sig=YzAc3kcEOyuA5XtNco2ESGPqifM&redir_esc=y#v=onepage&q&f=false](https://protect.checkpoint.com/v2/r02/___https:/books.google.no/books?hl=en&lr=&id=frqjEAAAQBAJ&oi=fnd&pg=PP1&dq=++(child*+OR+adolescen*+OR+youth+OR+teen*)+AND+(Mhealth+or+web*+or+telerehabilitation+or+interactive+or+mobile+or+smartphone*OR+cell+phone+OR+mobile+applications)+AND+(Craniocerebral+Trauma+OR+Spinal+Cord+Injuries+OR+Hydrocephalus+OR+Cerebral+Palsy+OR+Meningomyelocele+OR+Spina+Bifida+Cystica+OR+Spinal+Cord+Neoplasms)+&ots=FatGX2Gjn_&sig=YzAc3kcEOyuA5XtNco2ESGPqifM&redir_esc=y%23v=onepage&q&f=false___.YzJlOmRpcmVrdG9yYXRmb3JtZWRpc2luc2tlcHJvZHVrdGVyOmM6bzplZDNmY2I5NTQ2MjExMTQ4YjVmMDQ0NjBkZDM5OTUwYzo3OjJmNzM6NDRhOWM5MGYyNWMyYTdlZTA5NGI3YThiYmZhMzhjMzZjZDU2YzVlMGU1MjJlMGMyOTNjOTcyMzNlNGI1Njk4NjpwOlQ6Rg) |
| 5 | [AOCNR EPoster Abstract](https://protect.checkpoint.com/v2/r02/___https:/journals.lww.com/iaph/fulltext/2021/15001/aocnr_eposter_abstract.1.aspx___.YzJlOmRpcmVrdG9yYXRmb3JtZWRpc2luc2tlcHJvZHVrdGVyOmM6bzplZDNmY2I5NTQ2MjExMTQ4YjVmMDQ0NjBkZDM5OTUwYzo3OmVhMTU6OWIzZjExZGRjNjIzNjA4MDE1OGU2OWRhMDI5NTQ2M2U0ZDQzMGRmMjJlOWJlMjI3NGY1Mjg3OTkxZTdhNzQwZjpwOlQ6Rg) | [https://scholarshare.temple.edu/handle/20.500.12613/9260](https://protect.checkpoint.com/v2/r02/___https:/scholarshare.temple.edu/handle/20.500.12613/9260___.YzJlOmRpcmVrdG9yYXRmb3JtZWRpc2luc2tlcHJvZHVrdGVyOmM6bzplZDNmY2I5NTQ2MjExMTQ4YjVmMDQ0NjBkZDM5OTUwYzo3OmNlMWY6NWNmMDI5NjM4Mjg1OWM1ZmI2YThlZTk3ZTQ1M2UxODI0ZmQ4ZTZhNGViNDIzMWE3ODlmYTQ2ODhkN2U3OGNiNzpwOlQ6Rg) |
| 6 | Pelvic Floor Muscle Training Systematic Review Search Strategy | [https://scholarshare.temple.edu/handle/20.500.12613/9260](https://protect.checkpoint.com/v2/r02/___https:/scholarshare.temple.edu/handle/20.500.12613/9260___.YzJlOmRpcmVrdG9yYXRmb3JtZWRpc2luc2tlcHJvZHVrdGVyOmM6bzplZDNmY2I5NTQ2MjExMTQ4YjVmMDQ0NjBkZDM5OTUwYzo3OmNlMWY6NWNmMDI5NjM4Mjg1OWM1ZmI2YThlZTk3ZTQ1M2UxODI0ZmQ4ZTZhNGViNDIzMWE3ODlmYTQ2ODhkN2U3OGNiNzpwOlQ6Rg) |
| 7 | High Flow Nasal Cannula as respiratory support post-discharge in infants with bronchopulmonary dysplasia: description of a cohort of infants from a Northern … | [https://edotplus.ch/api/v1/public/euppt2024/events/euppt2024/media/3b2ba9e5582f4893aca6ab8c4ebadc72.pdf](https://protect.checkpoint.com/v2/r02/___https:/edotplus.ch/api/v1/public/euppt2024/events/euppt2024/media/3b2ba9e5582f4893aca6ab8c4ebadc72.pdf___.YzJlOmRpcmVrdG9yYXRmb3JtZWRpc2luc2tlcHJvZHVrdGVyOmM6bzplZDNmY2I5NTQ2MjExMTQ4YjVmMDQ0NjBkZDM5OTUwYzo3OmU0NDU6MmU4ZGIyZjU2NDQzMDk1MjQ5OWFjNGZlZjY2NjE3Mjk5YTliYTkwZjk5YTY4ODQxNGQ0ZDE2YmE4MDYyMzRhNjpwOlQ6Rg) |
| 8 | Peer support interventions for individuals with acquired brain injury, cerebral palsy, and spina bifida: a systematic review | [https://link.springer.com/article/10.1186/s12913-019-4110-5](https://protect.checkpoint.com/v2/r02/___https:/link.springer.com/article/10.1186/s12913-019-4110-5___.YzJlOmRpcmVrdG9yYXRmb3JtZWRpc2luc2tlcHJvZHVrdGVyOmM6bzplZDNmY2I5NTQ2MjExMTQ4YjVmMDQ0NjBkZDM5OTUwYzo3OjE3Njk6MzYzNTFjNzdhOTBhOWRiNWJjZWY2YjllMjIyYmZhZWNjZjkyNDZkOTY1MGY4Y2I4MWJjYjExMDRlNGFkNWM2MDpwOlQ6Rg) |
| 9 | Systematic review of telemedicine in spine surgery |  |
| 10 | Supporting the transition of children and young people with spinal cord injury from pediatric to adult healthcare services | [https://researchers-admin.westernsydney.edu.au/ws/portalfiles/portal/94849435/uws_71454.pdf](https://protect.checkpoint.com/v2/r02/___https:/researchers-admin.westernsydney.edu.au/ws/portalfiles/portal/94849435/uws_71454.pdf___.YzJlOmRpcmVrdG9yYXRmb3JtZWRpc2luc2tlcHJvZHVrdGVyOmM6bzplZDNmY2I5NTQ2MjExMTQ4YjVmMDQ0NjBkZDM5OTUwYzo3OjZjM2I6YjI1N2Q0OGJlMGFkYTBmNzY5ZmY3ZjJiMGI1NDE4ZDc3ZDI4ZDVjMzAyNDY3OWZiNTkxNzE4NjcyNjE4N2RlNzpwOlQ6Rg) |
| 11 | The impact of interactive telerehabilitiation on people with physical disabilities |  |
| 12 | Clinical Capacity Building Through Partnerships: Boots on the Ground in Global Neurosurgery | [https://journals.lww.com/neurosurgery/fulltext/2024/10000/clinical_capacity_building_through_partnerships_.3.aspx?context=latestarticles](https://protect.checkpoint.com/v2/r02/___https:/journals.lww.com/neurosurgery/fulltext/2024/10000/clinical_capacity_building_through_partnerships_.3.aspx?context=latestarticles___.YzJlOmRpcmVrdG9yYXRmb3JtZWRpc2luc2tlcHJvZHVrdGVyOmM6bzplZDNmY2I5NTQ2MjExMTQ4YjVmMDQ0NjBkZDM5OTUwYzo3OjRhZWY6YThkM2VkYjljMmE3OWMxZDRjNjI3MTZkMWJmMWU2NDZkODBlNmUxZTJmODdjN2IzZTdjMDFhNjRiMmQ1ZjBhZjpwOlQ6Rg) |
| 13 | The neurosurgical workforce of the Philippines | [https://journals.lww.com/neurosurgery/abstract/2024/01000/the_neurosurgical_workforce_of_the_philippines.23.aspx](https://protect.checkpoint.com/v2/r02/___https:/journals.lww.com/neurosurgery/abstract/2024/01000/the_neurosurgical_workforce_of_the_philippines.23.aspx___.YzJlOmRpcmVrdG9yYXRmb3JtZWRpc2luc2tlcHJvZHVrdGVyOmM6bzplZDNmY2I5NTQ2MjExMTQ4YjVmMDQ0NjBkZDM5OTUwYzo3OjkwY2Y6YmIzODVhOTg1Mzk2YjFmNjAxOTY0ZDRhZmRiMWFmOWE5MzNkZTQ0OTUxYzk3NDQwOWUxOTQyMDA0ZmQ2ODdiNTpwOlQ6Rg) |
| 14 | Pediatric hydrocephalus and the primary care provider | [https://www.pediatric.theclinics.com/article/S0031-3955(21)00052-3/abstract](https://protect.checkpoint.com/v2/r02/___https:/www.pediatric.theclinics.com/article/S0031-3955(21)00052-3/abstract___.YzJlOmRpcmVrdG9yYXRmb3JtZWRpc2luc2tlcHJvZHVrdGVyOmM6bzplZDNmY2I5NTQ2MjExMTQ4YjVmMDQ0NjBkZDM5OTUwYzo3OjdiNWE6YzYzOWM3ZjdkMTlhYTdiNzkyYzk2MTVmMTBiZTkzYWU4ZWNhMmEyMjg1MDVjMGU0NzAwNmI1ZDZjNjc0YTY0MTpwOlQ6Rg) |
| 15 | Remote follow-up technologies in traumatic brain injury: a scoping review | [https://www.liebertpub.com/doi/full/10.1089/neu.2022.0138](https://protect.checkpoint.com/v2/r02/___https:/www.liebertpub.com/doi/full/10.1089/neu.2022.0138___.YzJlOmRpcmVrdG9yYXRmb3JtZWRpc2luc2tlcHJvZHVrdGVyOmM6bzplZDNmY2I5NTQ2MjExMTQ4YjVmMDQ0NjBkZDM5OTUwYzo3OmE3YjU6NWY3YzBhNDcyMWQ2NDkwNDRkNzUyYmEyODM2MzFhMTJmODc3ZDYwZGUxMWU1N2I0ZmYyMzk2OGQ0ZTU4OTkxZTpwOlQ6Rg) |
| 16 | Spinal Cord Disabilities and Other Spinal Conditions | [https://books.google.no/books?id=43c4EAAAQBAJ&lpg=PT386&ots=M_U5ldu6VX&dq=%20%20(child*%20OR%20adolescen*%20OR%20youth%20OR%20teen*)%20AND%20(web*%20or%20interactive%20or%20mobile%20or%20smartphone*OR%20cell%20phone%20OR%20mobile%20applications)%20AND%20(brain%20trauma%20OR%20Spinal%20Cord%20Injuries%20OR%20Hydrocephalus%20OR%20Cerebral%20Palsy%20OR%20Meningomyelocele%20OR%20Spina%20Bifida%20Cystica%20OR%20Spinal%20Cord%20Neoplasms)%20&lr&pg=PT386#v=onepage&q&f=false](https://protect.checkpoint.com/v2/r02/___https:/books.google.no/books?id=43c4EAAAQBAJ&lpg=PT386&ots=M_U5ldu6VX&dq=%20%20(child*%20OR%20adolescen*%20OR%20youth%20OR%20teen*)%20AND%20(web*%20or%20interactive%20or%20mobile%20or%20smartphone*OR%20cell%20phone%20OR%20mobile%20applications)%20AND%20(brain%20trauma%20OR%20Spinal%20Cord%20Injuries%20OR%20Hydrocephalus%20OR%20Cerebral%20Palsy%20OR%20Meningomyelocele%20OR%20Spina%20Bifida%20Cystica%20OR%20Spinal%20Cord%20Neoplasms)%20&lr&pg=PT386%23v=onepage&q&f=false___.YzJlOmRpcmVrdG9yYXRmb3JtZWRpc2luc2tlcHJvZHVrdGVyOmM6bzplZDNmY2I5NTQ2MjExMTQ4YjVmMDQ0NjBkZDM5OTUwYzo3OmMyOTM6YzVhOGU4ODQ0YTM2MDE3NTQ2YjdiNDQwM2IyNDc1NGYyNTc5NjQ4ODNlYTYxNjhlMmE2MDU1Mjk5YmRmYmRiYTpwOlQ6Rg) |
| 17 | Physical Medicine & Rehabilitation Secrets: Physical Medicine & Rehabilitation Secrets | [https://books.google.no/books?id=frqjEAAAQBAJ&lpg=PP1&ots=FatGY-Cni4&dq=%20%20(child*%20OR%20adolescen*%20OR%20youth%20OR%20teen*)%20AND%20(web*%20or%20interactive%20or%20mobile%20or%20smartphone*OR%20cell%20phone%20OR%20mobile%20applications)%20AND%20(brain%20trauma%20OR%20Spinal%20Cord%20Injuries%20OR%20Hydrocephalus%20OR%20Cerebral%20Palsy%20OR%20Meningomyelocele%20OR%20Spina%20Bifida%20Cystica%20OR%20Spinal%20Cord%20Neoplasms)%20&lr&pg=PP1#v=onepage&q&f=false](https://protect.checkpoint.com/v2/r02/___https:/books.google.no/books?id=frqjEAAAQBAJ&lpg=PP1&ots=FatGY-Cni4&dq=%20%20(child*%20OR%20adolescen*%20OR%20youth%20OR%20teen*)%20AND%20(web*%20or%20interactive%20or%20mobile%20or%20smartphone*OR%20cell%20phone%20OR%20mobile%20applications)%20AND%20(brain%20trauma%20OR%20Spinal%20Cord%20Injuries%20OR%20Hydrocephalus%20OR%20Cerebral%20Palsy%20OR%20Meningomyelocele%20OR%20Spina%20Bifida%20Cystica%20OR%20Spinal%20Cord%20Neoplasms)%20&lr&pg=PP1%23v=onepage&q&f=false___.YzJlOmRpcmVrdG9yYXRmb3JtZWRpc2luc2tlcHJvZHVrdGVyOmM6bzplZDNmY2I5NTQ2MjExMTQ4YjVmMDQ0NjBkZDM5OTUwYzo3OmY5Yjk6ODkwYWI2ZmNmMDFiY2I2MThjZDA0Njk2M2Q0OTFiYmZhNTQzNzc0OWJjMjk3MzY1MThmNjcyNGY3OGE0ZWNmYzpwOlQ6Rg) |
| 18 | Sitting analysis in wheelchair tennis | [https://upcommons.upc.edu/handle/2117/383005](https://protect.checkpoint.com/v2/r02/___https:/upcommons.upc.edu/handle/2117/383005___.YzJlOmRpcmVrdG9yYXRmb3JtZWRpc2luc2tlcHJvZHVrdGVyOmM6bzplZDNmY2I5NTQ2MjExMTQ4YjVmMDQ0NjBkZDM5OTUwYzo3OjI0N2U6ZjYwMjI5NDU4NTljMmY1Mjg0MmI3OGY0ZjQ2YmM0NWY1NmU5MjY3MmQ1MWM1ZDgxZDlmZTM5MDMwMmMyNGVmMTpwOlQ6Rg) |
| 19 | Intelligent robotics in pediatric cooperative neurorehabilitation: a review | [https://www.mdpi.com/2218-6581/13/3/49](https://protect.checkpoint.com/v2/r02/___https:/www.mdpi.com/2218-6581/13/3/49___.YzJlOmRpcmVrdG9yYXRmb3JtZWRpc2luc2tlcHJvZHVrdGVyOmM6bzplZDNmY2I5NTQ2MjExMTQ4YjVmMDQ0NjBkZDM5OTUwYzo3OmIyNDc6MmE2MWE2YWY4ZGY0ZDI2YjM0ZWQ5N2Y1MDFmYjlhM2ViMzgzMThhMmQ3MGM1YjM4YTg0NjM5NWY1YTEzNDk2NTpwOlQ6Rg) |
| 20 | Neuroscience Research notes | [https://www.researchgate.net/profile/Noraishah-Abdul-Aziz/publication/361563655_I_have_spina_bifida_and_what_this_means_to_Malaysia/links/62c5562834fb735279380b47/I-have-spina-bifida-and-what-this-means-to-Malaysia.pdf?_sg%5B0%5D=started_experiment_milestone&origin=journalDetail](https://protect.checkpoint.com/v2/r02/___https:/www.researchgate.net/profile/Noraishah-Abdul-Aziz/publication/361563655_I_have_spina_bifida_and_what_this_means_to_Malaysia/links/62c5562834fb735279380b47/I-have-spina-bifida-and-what-this-means-to-Malaysia.pdf?_sg%5B0%5D=started_experiment_milestone&origin=journalDetail___.YzJlOmRpcmVrdG9yYXRmb3JtZWRpc2luc2tlcHJvZHVrdGVyOmM6bzplZDNmY2I5NTQ2MjExMTQ4YjVmMDQ0NjBkZDM5OTUwYzo3OjAxZGQ6NDkwOTA0NDMyYWU3YTBiMDAwNjNhN2FmNjQ5MDgyNjJkYWZmMDJkNzJmNzcyYzk2NzhiMTg1MzFmZmQ2ODNiNjpwOlQ6Rg) |
| 21 | Factors associated with falls among wheelchair users living with spinal cord injury | [https://www.ideals.illinois.edu/items/124716](https://protect.checkpoint.com/v2/r02/___https:/www.ideals.illinois.edu/items/124716___.YzJlOmRpcmVrdG9yYXRmb3JtZWRpc2luc2tlcHJvZHVrdGVyOmM6bzplZDNmY2I5NTQ2MjExMTQ4YjVmMDQ0NjBkZDM5OTUwYzo3OmQwYzY6MzA0NDg3YTFhYmE5MDMwZTQxNTY5MGZmZGZlMzUwYTU3MTk5OGRhZDUzOWI3NmE0MDgwOTBkN2Q3NTU0YmE2MzpwOlQ6Rg) |
| 22 | Weight management interventions that include dietary components for young people with chronic health care needs: a systematic review | [https://onlinelibrary.wiley.com/doi/abs/10.1111/1747-0080.12698?casa_token=E9DbM7IRVTgAAAAA%3AZoP1rKRAy0ZEaUWEZvc3o4WV3e_jtAjF6pW2T8fr0aOy6CJoOckQRFapWpH-2nJQyWXFjCPmjQ6fHv18](https://protect.checkpoint.com/v2/r02/___https:/onlinelibrary.wiley.com/doi/abs/10.1111/1747-0080.12698?casa_token=E9DbM7IRVTgAAAAA%3AZoP1rKRAy0ZEaUWEZvc3o4WV3e_jtAjF6pW2T8fr0aOy6CJoOckQRFapWpH-2nJQyWXFjCPmjQ6fHv18___.YzJlOmRpcmVrdG9yYXRmb3JtZWRpc2luc2tlcHJvZHVrdGVyOmM6bzplZDNmY2I5NTQ2MjExMTQ4YjVmMDQ0NjBkZDM5OTUwYzo3OmYyNTg6MmJhNjczMjY5ZmE5NDQyZjFkZDFhNWZlMDFiZWIyYjQxYjc3MDM5ODlmYmQyYzVjOGQ1ZGM5OWI2YWE4MTk4YzpwOlQ6Rg) |
| 23 | Asian Spinal Cord Network (ASCoN) Conference 2023 | [http://202.4.109.28:8080/xmlui/handle/123456789/922](https://protect.checkpoint.com/v2/r02/___http:/202.4.109.28:8080/xmlui/handle/123456789/922___.YzJlOmRpcmVrdG9yYXRmb3JtZWRpc2luc2tlcHJvZHVrdGVyOmM6bzplZDNmY2I5NTQ2MjExMTQ4YjVmMDQ0NjBkZDM5OTUwYzo3OmE5MmI6YjAwOTFiODYwZjhiNWRlMDIyMmFmNDNiYjhmMWVmNDA4NWNmMWYxMjRiODA0N2ZkMGU1ZTQyODNlZGIyZjNmMDpwOlQ6Rg) |
| 24 | Clinical application of rehabilitation therapy technologies to children with CNS damage | [https://link.springer.com/chapter/10.1007/978-3-031-08995-4_14](https://protect.checkpoint.com/v2/r02/___https:/link.springer.com/chapter/10.1007/978-3-031-08995-4_14___.YzJlOmRpcmVrdG9yYXRmb3JtZWRpc2luc2tlcHJvZHVrdGVyOmM6bzplZDNmY2I5NTQ2MjExMTQ4YjVmMDQ0NjBkZDM5OTUwYzo3OjhlMzk6YjViNDg0NDlmNzZkMGIwOTA5MWZlZjEwM2I0YTAwZjVhYzQ5NTA0MGRlNTEyNzc5NTc4ZDg4NzIwNWRmOWY3YjpwOlQ6Rg) |
| 25 | A survey of robots in healthcare | [https://www.mdpi.com/2227-7080/9/1/8](https://protect.checkpoint.com/v2/r02/___https:/www.mdpi.com/2227-7080/9/1/8___.YzJlOmRpcmVrdG9yYXRmb3JtZWRpc2luc2tlcHJvZHVrdGVyOmM6bzplZDNmY2I5NTQ2MjExMTQ4YjVmMDQ0NjBkZDM5OTUwYzo3OjJkNmY6MzcxM2JlN2NkZTdlYmM0N2ZjMzg2MTVjNjg1NjA4OTNhZGIzMjdkMWRjM2Y5NDg1MzFlMGFmN2VkMDZiOTA5ZjpwOlQ6Rg) |
| 26 | American academy of spinal cord injury professionals ASCIP 2019 educational conference & expo September 1–4, 2019 Omni Hotel, Nashville, TN, USA | [https://pmc.ncbi.nlm.nih.gov/articles/PMC6758623/](https://protect.checkpoint.com/v2/r02/___https:/pmc.ncbi.nlm.nih.gov/articles/PMC6758623/___.YzJlOmRpcmVrdG9yYXRmb3JtZWRpc2luc2tlcHJvZHVrdGVyOmM6bzplZDNmY2I5NTQ2MjExMTQ4YjVmMDQ0NjBkZDM5OTUwYzo3OjdhM2M6ZjU3NDQ1YTg0ODExODFkMmI4ODIwM2FiZDVmNjQ5N2RhYmEzZjI4NTZkZjBkOTliZDViZWI0YmQ3N2UyNGVjYjpwOlQ6Rg) |
| 27 | Pelvic Floor Muscle Training Systematic Review Search Strategy |  |
| 28 | Disability across the developmental lifespan: An introduction for the helping professions | [https://books.google.no/books?id=FuiZDwAAQBAJ&lpg=PP1&ots=FVK9zsKl1C&dq=%20%20(child*%20OR%20adolescen*%20OR%20youth%20OR%20teen*)%20AND%20(web*%20or%20interactive%20or%20mobile%20or%20smartphone*OR%20cell%20phone%20OR%20mobile%20applications)%20AND%20(brain%20trauma%20OR%20Spinal%20Cord%20Injuries%20OR%20Hydrocephalus%20OR%20Cerebral%20Palsy%20OR%20Meningomyelocele%20OR%20Spina%20Bifida%20Cystica%20OR%20Spinal%20Cord%20Neoplasms)%20&lr&pg=PP1#v=onepage&q&f=false](https://protect.checkpoint.com/v2/r02/___https:/books.google.no/books?id=FuiZDwAAQBAJ&lpg=PP1&ots=FVK9zsKl1C&dq=%20%20(child*%20OR%20adolescen*%20OR%20youth%20OR%20teen*)%20AND%20(web*%20or%20interactive%20or%20mobile%20or%20smartphone*OR%20cell%20phone%20OR%20mobile%20applications)%20AND%20(brain%20trauma%20OR%20Spinal%20Cord%20Injuries%20OR%20Hydrocephalus%20OR%20Cerebral%20Palsy%20OR%20Meningomyelocele%20OR%20Spina%20Bifida%20Cystica%20OR%20Spinal%20Cord%20Neoplasms)%20&lr&pg=PP1%23v=onepage&q&f=false___.YzJlOmRpcmVrdG9yYXRmb3JtZWRpc2luc2tlcHJvZHVrdGVyOmM6bzplZDNmY2I5NTQ2MjExMTQ4YjVmMDQ0NjBkZDM5OTUwYzo3OjE1NjY6Y2EzMjg2YjFkNGMzM2U3YmRkYTUzNjE5ZjAxOWE0OWI4OGUwYjk1NDc4M2JiYzNlZDIwYmNmMzNlZDFjMTU5YzpwOlQ6Rg) |
| 29 | ScreenABLE: Breast cancer screening among women with disabilities from community identified challenges to community-based programs | [https://muse.jhu.edu/article/730024](https://protect.checkpoint.com/v2/r02/___https:/muse.jhu.edu/article/730024___.YzJlOmRpcmVrdG9yYXRmb3JtZWRpc2luc2tlcHJvZHVrdGVyOmM6bzplZDNmY2I5NTQ2MjExMTQ4YjVmMDQ0NjBkZDM5OTUwYzo3OmIyZjU6NmNjMjU1NjM4NGNlOTg0ZmExMjVlMjYzZjgzZWVjNzAyNDUxYTMwNTFjZGRkYzRjNTJkMmYyOTczM2QyMGM5NDpwOlQ6Rg) |
| 30 | Surgical outcome of spinal subdural abscesses: a report of four consecutive patients and brief review of the literature | [https://www.thieme-connect.com/products/ejournals/abstract/10.1055/s-0041-1739210](https://protect.checkpoint.com/v2/r02/___https:/www.thieme-connect.com/products/ejournals/abstract/10.1055/s-0041-1739210___.YzJlOmRpcmVrdG9yYXRmb3JtZWRpc2luc2tlcHJvZHVrdGVyOmM6bzplZDNmY2I5NTQ2MjExMTQ4YjVmMDQ0NjBkZDM5OTUwYzo3OjA4MjI6YjhlMzMxZmIwZGQwMWU2OTQzODE0ZmMwMzRlMDQ0NTUwNmFkNTEzZjkzZGY5NTQ2YmYxMmQxOTNhZTkzZTg1MTpwOlQ6Rg) |
| 31 | IoT based brain-wave assistive system for paralyzed individuals | [https://dspace.bracu.ac.bd/xmlui/handle/10361/14519](https://protect.checkpoint.com/v2/r02/___https:/dspace.bracu.ac.bd/xmlui/handle/10361/14519___.YzJlOmRpcmVrdG9yYXRmb3JtZWRpc2luc2tlcHJvZHVrdGVyOmM6bzplZDNmY2I5NTQ2MjExMTQ4YjVmMDQ0NjBkZDM5OTUwYzo3OmQyMjM6NDZmODQwOTNlZjc3YWFmNzY0NGFiODNjY2E0NjlmMDhmYWIzZGZhOTM3YWExZTBiZmE0MDJjN2M1MmI5Yzc5NjpwOlQ6Rg) |
| 32 | Scientific Posters Friday, September 20, 2019 3: 30pm–4: 30pm | [https://openurl.ebsco.com/EPDB%3Agcd%3A15%3A24485199/detailv2?sid=ebsco%3Aplink%3Ascholar&id=ebsco%3Agcd%3A138688621&crl=c&link_origin=scholar.google.com](https://protect.checkpoint.com/v2/r02/___https:/openurl.ebsco.com/EPDB%3Agcd%3A15%3A24485199/detailv2?sid=ebsco%3Aplink%3Ascholar&id=ebsco%3Agcd%3A138688621&crl=c&link_origin=scholar.google.com___.YzJlOmRpcmVrdG9yYXRmb3JtZWRpc2luc2tlcHJvZHVrdGVyOmM6bzplZDNmY2I5NTQ2MjExMTQ4YjVmMDQ0NjBkZDM5OTUwYzo3OjgwMzE6NmM3ZDJjODM4ODY2ZGE3ZTJjMzMzYmQwMTg0MmQwZjRjMTcwN2Q3NDBkYjA5Yzc2MzVmN2JmZDljYWFhNzc5NTpwOlQ6Rg) |
| 33 | Deconstructing the delivery of peer mentorship for persons with spinal cord injury | [https://open.library.ubc.ca/soa/cIRcle/collections/ubctheses/24/items/1.0398717](https://protect.checkpoint.com/v2/r02/___https:/open.library.ubc.ca/soa/cIRcle/collections/ubctheses/24/items/1.0398717___.YzJlOmRpcmVrdG9yYXRmb3JtZWRpc2luc2tlcHJvZHVrdGVyOmM6bzplZDNmY2I5NTQ2MjExMTQ4YjVmMDQ0NjBkZDM5OTUwYzo3OjBlMmM6MzhmZWVkYWY3NTNjM2NlZTA4NjNlZWYyNGZkOWNiMjc2MGVmMWQ0NzViNjg5NzFhZjQyY2FlYzRlZTgxOTQwODpwOlQ6Rg) |
| 34 | Home-based physiotherapy programmes for individuals with neurological diseases: systematic review | [https://www.scielo.br/j/fm/a/YjMV5YyjM49kTwHVyCHttMp/?lang=en](https://protect.checkpoint.com/v2/r02/___https:/www.scielo.br/j/fm/a/YjMV5YyjM49kTwHVyCHttMp/?lang=en___.YzJlOmRpcmVrdG9yYXRmb3JtZWRpc2luc2tlcHJvZHVrdGVyOmM6bzplZDNmY2I5NTQ2MjExMTQ4YjVmMDQ0NjBkZDM5OTUwYzo3OmRiMDI6NGQ3NGQ5Mjc0M2E5OWU0MzkxMTAyYWUxY2I5ZjM0M2ZiODY4NzJiZGY5MTU5MzkxZGNiNGE1N2ExODIyZmU4MDpwOlQ6Rg) |
| 35 | [TUESDAY 5TH APRIL 2022](https://protect.checkpoint.com/v2/r02/___http:/oceaniaseatingsymposium.com/assets/sm/upload/56/2h/fh/kk/Tuesday%205th%20April%20Abstracts.pdf___.YzJlOmRpcmVrdG9yYXRmb3JtZWRpc2luc2tlcHJvZHVrdGVyOmM6bzplZDNmY2I5NTQ2MjExMTQ4YjVmMDQ0NjBkZDM5OTUwYzo3OjhkNzE6MmVhZWVhYmMzOWZlMjZkYTEzY2JlMzRkNjI3NGVhNTgxNTFlMmJkMWUzZWYwMWFiNzNhZmNjNDkzZjk2MTUzODpwOlQ6Rg) | [https://oceaniaseatingsymposium.com/assets/sm/upload/56/2h/fh/kk/Tuesday%205th%20April%20Abstracts.pdf](https://protect.checkpoint.com/v2/r02/___https:/oceaniaseatingsymposium.com/assets/sm/upload/56/2h/fh/kk/Tuesday%205th%20April%20Abstracts.pdf___.YzJlOmRpcmVrdG9yYXRmb3JtZWRpc2luc2tlcHJvZHVrdGVyOmM6bzplZDNmY2I5NTQ2MjExMTQ4YjVmMDQ0NjBkZDM5OTUwYzo3OmQ5NmU6NGZhMmNiZjM0MTVlY2UyN2I4OTIxZjE4OTY5N2U0NGUyOGVlMzE2ODdjMjMwYWIzNGZjMGNkOWMxMTM3NGE4OTpwOlQ6Rg) |
| 36 | Basics of child neuropsychology: A primer for educators and clinicians | [Basics of child neuropsychology: A primer for educators and clinicians](https://protect.checkpoint.com/v2/r02/___https:/books.google.com/books?hl=en&lr=&id=rdiEEAAAQBAJ&oi=fnd&pg=PP1&dq=++(child*+OR+adolescen*+OR+youth+OR+teen*)+AND+(web*+or+interactive+or+mobile+or+smartphone*OR+cell+phone+OR+mobile+applications)+AND+(brain+trauma+OR+Spinal+Cord+Injuries+OR+Hydrocephalus+OR+Cerebral+Palsy+OR+Meningomyelocele+OR+Spina+Bifida+Cystica+OR+Spinal+Cord+Neoplasms)+&ots=vSkxYdgssc&sig=sz4obw8L2BwxnKYLGcO4F3EcLPg___.YzJlOmRpcmVrdG9yYXRmb3JtZWRpc2luc2tlcHJvZHVrdGVyOmM6bzplZDNmY2I5NTQ2MjExMTQ4YjVmMDQ0NjBkZDM5OTUwYzo3OjYyNWE6ODg5OWQ0YmEwYThkN2I2MmU4OWIxZDEwYzc1NGFmMzcyZTg5MDI3MTVjNmY5MDY0ZGE2ZTlmOGVmNjMzY2UxZjpwOlQ6Rg) |
| 37 | AOCNR EPoster Abstract | [https://journals.lww.com/iaph/fulltext/2021/15001/aocnr_eposter_abstract.1.aspx](https://protect.checkpoint.com/v2/r02/___https:/journals.lww.com/iaph/fulltext/2021/15001/aocnr_eposter_abstract.1.aspx___.YzJlOmRpcmVrdG9yYXRmb3JtZWRpc2luc2tlcHJvZHVrdGVyOmM6bzplZDNmY2I5NTQ2MjExMTQ4YjVmMDQ0NjBkZDM5OTUwYzo3OmVhMTU6OWIzZjExZGRjNjIzNjA4MDE1OGU2OWRhMDI5NTQ2M2U0ZDQzMGRmMjJlOWJlMjI3NGY1Mjg3OTkxZTdhNzQwZjpwOlQ6Rg) |
| 38 | Physical Therapy Poster Presentations at the Combined Sections Meeting | [https://journals.lww.com/pedpt/fulltext/2023/01000/abstracts_of_the_academy_of_pediatric_physical.24.aspx](https://protect.checkpoint.com/v2/r02/___https:/journals.lww.com/pedpt/fulltext/2023/01000/abstracts_of_the_academy_of_pediatric_physical.24.aspx___.YzJlOmRpcmVrdG9yYXRmb3JtZWRpc2luc2tlcHJvZHVrdGVyOmM6bzplZDNmY2I5NTQ2MjExMTQ4YjVmMDQ0NjBkZDM5OTUwYzo3OmY3ZDQ6MjgzNWNkOGE5YWU2MjJiMDlhYWUyNzExNzhjMDA4NWNhY2Y0OWNiYzJiMTk0ODZjYmU2NTI4Yzc5YzVlMGE5ZjpwOlQ6Rg) |
| 39 | Presented at Physiatry'24 | [https://journals.lww.com/ajpmr/citation/2024/05002/abstracts_of_scientific_papers_and_posters.1.aspx](https://protect.checkpoint.com/v2/r02/___https:/journals.lww.com/ajpmr/citation/2024/05002/abstracts_of_scientific_papers_and_posters.1.aspx___.YzJlOmRpcmVrdG9yYXRmb3JtZWRpc2luc2tlcHJvZHVrdGVyOmM6bzplZDNmY2I5NTQ2MjExMTQ4YjVmMDQ0NjBkZDM5OTUwYzo3OjAwNjQ6OWM2OWI2ODUzMTg4MWM1N2EzMzg2NGFkZDhmN2ViNGJiMjI4OTU1MmYwYWNiYTMzODRjZjNkMTYwYTUzOWI2YjpwOlQ6Rg) |
| 40 | Exosomes as neurological nanosized machines | [https://pubs.acs.org/doi/full/10.1021/acsnanoscienceau.1c00062](https://protect.checkpoint.com/v2/r02/___https:/pubs.acs.org/doi/full/10.1021/acsnanoscienceau.1c00062___.YzJlOmRpcmVrdG9yYXRmb3JtZWRpc2luc2tlcHJvZHVrdGVyOmM6bzplZDNmY2I5NTQ2MjExMTQ4YjVmMDQ0NjBkZDM5OTUwYzo3OjMyZWE6NGQyODA2Y2FlZWQzYTVjZDE2ZDZjYTkzM2NkNDM5MWQ1MzczNDRjNGY1NjYwZTVjNDQzNGFmNjI3YTQ1YTFiNzpwOlQ6Rg) |
| 41 | The Musculoskeletal Disorders, Pain Characteristics, and Activity Levels of Caregivers During the Rehabilitation Process and the Consequences on Sleep Condition … | [https://www.thieme-connect.com/products/ejournals/abstract/10.1055/a-1617-4339](https://protect.checkpoint.com/v2/r02/___https:/www.thieme-connect.com/products/ejournals/abstract/10.1055/a-1617-4339___.YzJlOmRpcmVrdG9yYXRmb3JtZWRpc2luc2tlcHJvZHVrdGVyOmM6bzplZDNmY2I5NTQ2MjExMTQ4YjVmMDQ0NjBkZDM5OTUwYzo3OmY3ZDA6MDY2OWU4MjAzN2ViZWFhMjcyYmQ3OGIxNTY2MTQ3MmEwMTY3Nzg2NmNiMDY5ZTY4YWJlYWY3MDQwMGNhMmUyNTpwOlQ6Rg) |
| 42 | The Disability Experience: Working Toward Belonging | The Disability Experience: Working Toward Belonging |
| 43 | Brain Tumor Targeting Drug Delivery Systems: Advanced Nanoscience for Theranostics Applications | Brain Tumor Targeting Drug Delivery Systems: Advanced Nanoscience for Theranostics Applications |
| 44 | World Congress and Annual Meeting of the Association of Academic Physiatrists | [https://journals.lww.com/ajpmr/fulltext/2020/03001/abstracts_of_scientific_papers_and_posters.1.aspx?casa_token=tfvn8hzuAmkAAAAA:Ajwkp4fXAbTOu68srZKELuJiIxVsyBd3rP7oWfmwCsdZDJ7LaMp5ZTSkXfowrZSCzAD3M7Sf71BOMMtgFiKRFGDc&casa_token=pvqMjhuIUqwAAAAA:g7JviRKFRpfS5mQ42MxLyUqzB4aHYcjj5Ry1a3yexeA2dsEXo5Zzq3IuvS6cBHA0do-tfGdIMbm5CBGQhcvVK0-9](https://protect.checkpoint.com/v2/r02/___https:/journals.lww.com/ajpmr/fulltext/2020/03001/abstracts_of_scientific_papers_and_posters.1.aspx?casa_token=tfvn8hzuAmkAAAAA:Ajwkp4fXAbTOu68srZKELuJiIxVsyBd3rP7oWfmwCsdZDJ7LaMp5ZTSkXfowrZSCzAD3M7Sf71BOMMtgFiKRFGDc&casa_token=pvqMjhuIUqwAAAAA:g7JviRKFRpfS5mQ42MxLyUqzB4aHYcjj5Ry1a3yexeA2dsEXo5Zzq3IuvS6cBHA0do-tfGdIMbm5CBGQhcvVK0-9___.YzJlOmRpcmVrdG9yYXRmb3JtZWRpc2luc2tlcHJvZHVrdGVyOmM6bzplZDNmY2I5NTQ2MjExMTQ4YjVmMDQ0NjBkZDM5OTUwYzo3OjAxMzQ6YzNhNjZiYzM3ZmE3OTE2NThkM2VhNmZjYmRlMTE3NzE2MTNlMWNkYTgxNTEyZWM2OWQ5MmNkYzhiOTAxMzAxODpwOlQ6Rg) |
| 45 | Designing Socially Assistive Robots for Paediatric Rehabilitation (In Situ Design for Ongoing Clinical Deployment) | [https://figshare.swinburne.edu.au/articles/thesis/Designing_Socially_Assistive_Robots_for_Paediatric_Rehabilitation_In_Situ_Design_for_Ongoing_Clinical_Deployment_/26297050?file=47665009](https://protect.checkpoint.com/v2/r02/___https:/figshare.swinburne.edu.au/articles/thesis/Designing_Socially_Assistive_Robots_for_Paediatric_Rehabilitation_In_Situ_Design_for_Ongoing_Clinical_Deployment_/26297050?file=47665009___.YzJlOmRpcmVrdG9yYXRmb3JtZWRpc2luc2tlcHJvZHVrdGVyOmM6bzplZDNmY2I5NTQ2MjExMTQ4YjVmMDQ0NjBkZDM5OTUwYzo3OjkzZDg6YjEwYmUyN2ZjMjBjOTYzYzU1YjFlMWNhOThiYjdlY2RjOGVhMjIzOTZlOWQ2YzYxYjI3YjVkNjkyYzQ4MWNjMzpwOlQ6Rg) |
| 46 | Management of Patient with Hydrocephalus | [https://link.springer.com/chapter/10.1007/978-981-15-0458-7_11](https://protect.checkpoint.com/v2/r02/___https:/link.springer.com/chapter/10.1007/978-981-15-0458-7_11___.YzJlOmRpcmVrdG9yYXRmb3JtZWRpc2luc2tlcHJvZHVrdGVyOmM6bzplZDNmY2I5NTQ2MjExMTQ4YjVmMDQ0NjBkZDM5OTUwYzo3OmI0YzQ6ZmU4ODg4YzhkNTgyYTM1MzIxNmM5ZDQzMTUwNWZkNGE4NjZjM2U4MDA4MmQ0NTJmYzRlODNiNzhhYzE3OWE0MjpwOlQ6Rg) |
| 47 | Key Conditions and Principles of Orthopaedic Management | [https://onlinelibrary.wiley.com/doi/abs/10.1002/9781119833413.ch13](https://protect.checkpoint.com/v2/r02/___https:/onlinelibrary.wiley.com/doi/abs/10.1002/9781119833413.ch13___.YzJlOmRpcmVrdG9yYXRmb3JtZWRpc2luc2tlcHJvZHVrdGVyOmM6bzplZDNmY2I5NTQ2MjExMTQ4YjVmMDQ0NjBkZDM5OTUwYzo3OjYzNTA6YmU1NWU0ZjhkZTMwYWJmNzE5NGMyMGYzZGQ2YjBmM2E1ZWZmYTU5ZDE2YjYxZWIxNjZkYjljZmYyMDA1NDIzNjpwOlQ6Rg) |
| 48 | Sinai Health tests new robotic surgery application | https://www.canhealth.com/wp-content/uploads/2024/11/Canadian-Healthcare-Technology-2024-08.pdf |
| 49 | High Flow Nasal Cannula as respiratory support post-discharge in infants with bronchopulmonary dysplasia: description of a cohort of infants from a Northern … | |
| 50 | Dear Colleagues | https://my.clevelandclinic.org/-/scassets/files/org/obgyn/whi-year-in-review-2019.pdf |
| 51 | Brain and spinal tumors of childhood | [Brain and spinal tumors of childhood](https://protect.checkpoint.com/v2/r02/___https:/api.taylorfrancis.com/content/books/mono/download?identifierName=doi&identifierValue=10.1201/9781003090120&type=googlepdf___.YzJlOmRpcmVrdG9yYXRmb3JtZWRpc2luc2tlcHJvZHVrdGVyOmM6bzplZDNmY2I5NTQ2MjExMTQ4YjVmMDQ0NjBkZDM5OTUwYzo3OjE5M2M6NGFjNWFjYjZkNGE3MWI0MzJkMWJmNTA4NjM2YjJmYjE5ZGVmZDkxM2M2NjdiNmZiYmZlNzQxOTRkYjViNWQyYzpwOlQ6Rg) |
| 52 | Physical activity barriers in wheelchair users, and cardiometabolic health effects of upper-body rowing exercise in individuals with spinal cord injury | [https://vbn.aau.dk/ws/portalfiles/portal/549539566/phd_RKH_e_pdf.pdf](https://protect.checkpoint.com/v2/r02/___https:/vbn.aau.dk/ws/portalfiles/portal/549539566/phd_RKH_e_pdf.pdf___.YzJlOmRpcmVrdG9yYXRmb3JtZWRpc2luc2tlcHJvZHVrdGVyOmM6bzplZDNmY2I5NTQ2MjExMTQ4YjVmMDQ0NjBkZDM5OTUwYzo3OjY1NGE6NGE3ZjQyOTA1ZDI5YjI1Y2E4YzRlNGNiYjFmYWJkNTQ2YmRkZmZhMDU5ZThlNDg4NDY4ZDJhZDdmODFlZmYzNzpwOlQ6Rg) |
| 53 | A Case Study of Educational Resources for Parents of Chronically Ill Students | [https://www.proquest.com/docview/2455950058?fromopenview=true&pq-origsite=gscholar&sourcetype=Dissertations%20&%20Theses](https://protect.checkpoint.com/v2/r02/___https:/www.proquest.com/docview/2455950058?fromopenview=true&pq-origsite=gscholar&sourcetype=Dissertations%20&%20Theses___.YzJlOmRpcmVrdG9yYXRmb3JtZWRpc2luc2tlcHJvZHVrdGVyOmM6bzplZDNmY2I5NTQ2MjExMTQ4YjVmMDQ0NjBkZDM5OTUwYzo3OjE3MDc6MDg0MWEzZGJhMjZjOTQzNmE0MjI3OTI4NmY2Nzc3MjhmYjM4NTg0MzI2MDMwNDcwZTE4OGM1ZjhlNmYwNmNjNTpwOlQ6Rg) |
| 54 | Department of Urology | [https://www.jefferson.edu/content/dam/academic/skmc/departments/urology/documents/Jefferson-Urology-YIR2020.pdf](https://protect.checkpoint.com/v2/r02/___https:/www.jefferson.edu/content/dam/academic/skmc/departments/urology/documents/Jefferson-Urology-YIR2020.pdf___.YzJlOmRpcmVrdG9yYXRmb3JtZWRpc2luc2tlcHJvZHVrdGVyOmM6bzplZDNmY2I5NTQ2MjExMTQ4YjVmMDQ0NjBkZDM5OTUwYzo3OmUwZWE6MTUyMmFjZWIwZGNjNGIzMjUwOGU2Zjg1ODg5YzVhYjMyMThhNGVhNmZhMjJjZWM4NGU2ZmFmODI4NGMzNDk3ZjpwOlQ6Rg) |
| 55 | Validity of two wheelchair-mounted devices for estimating wheelchair speed and distance traveled | [https://journals.humankinetics.com/view/journals/apaq/38/3/article-p435.xml](https://protect.checkpoint.com/v2/r02/___https:/journals.humankinetics.com/view/journals/apaq/38/3/article-p435.xml___.YzJlOmRpcmVrdG9yYXRmb3JtZWRpc2luc2tlcHJvZHVrdGVyOmM6bzplZDNmY2I5NTQ2MjExMTQ4YjVmMDQ0NjBkZDM5OTUwYzo3OjMyNGI6NzJkMmE3ZWQ4OTc0MDk0ZTY4OGQ1OTZlZDU4M2IzM2E5MzVhYmQ4MWNiNGNmN2E3MjBjNWIxZjg4NTI0ZjY1MzpwOlQ6Rg) |
| 56 | Neurological bedside examination:“can i confirm my anatomical hypothesis?” | [https://link.springer.com/chapter/10.1007/978-3-031-43335-1_3](https://protect.checkpoint.com/v2/r02/___https:/link.springer.com/chapter/10.1007/978-3-031-43335-1_3___.YzJlOmRpcmVrdG9yYXRmb3JtZWRpc2luc2tlcHJvZHVrdGVyOmM6bzplZDNmY2I5NTQ2MjExMTQ4YjVmMDQ0NjBkZDM5OTUwYzo3Ojk4M2E6ZGY2NDlkYWZmMDA4OTA5YzhiOTljODVjZWZkOTM5YTA1ODUzMThjYzFhNzI0ZGY4YTY1YjcwMWQyYzBkY2FmYjpwOlQ6Rg) |
| 57 | California Children's Services Program: Title V Needs Assessment 2018-2019 | [https://fhop.ucsf.edu/sites/g/files/tkssra4481/f/DRAFT_Presentation_for_final_Stakeholder_meeting_9-25-19_CORRECTED.pdf](https://protect.checkpoint.com/v2/r02/___https:/fhop.ucsf.edu/sites/g/files/tkssra4481/f/DRAFT_Presentation_for_final_Stakeholder_meeting_9-25-19_CORRECTED.pdf___.YzJlOmRpcmVrdG9yYXRmb3JtZWRpc2luc2tlcHJvZHVrdGVyOmM6bzplZDNmY2I5NTQ2MjExMTQ4YjVmMDQ0NjBkZDM5OTUwYzo3OmM4OTY6NDYzMWI5ZDc0NDI5OWE0NThjNzJkMGIwZmMwM2I1OTZkMjZhYmZmZGU3MGY2ZTRlY2Y0NWEwMTc1ODBlNWViODpwOlQ6Rg) |
| 58 | Mobile applications in children with cerebral palsy | [https://www.researchgate.net/publication/335107997_Mobile_applications_in_children_with_cerebral_palsy](https://protect.checkpoint.com/v2/r02/___https:/www.researchgate.net/publication/335107997_Mobile_applications_in_children_with_cerebral_palsy___.YzJlOmRpcmVrdG9yYXRmb3JtZWRpc2luc2tlcHJvZHVrdGVyOmM6bzplZDNmY2I5NTQ2MjExMTQ4YjVmMDQ0NjBkZDM5OTUwYzo3OjljZTY6ZTgzYTM3Y2M1YzVlNGEwN2Y2OTI3YWI2Y2IyMmRkOTNlYzk5ZjMyZGJkZWY1Y2U3ZDhjYjNiZDc0ZDZkNjUwNDpwOlQ6Rg) |
| 70 | Interventions in the Home and Community for Medically Complex Children: A Systematic Review | [https://publications.aap.org/pediatrics/article/151/5/e2022058352/191050/Interventions-in-the-Home-and-Community-for](https://protect.checkpoint.com/v2/r02/___https:/publications.aap.org/pediatrics/article/151/5/e2022058352/191050/Interventions-in-the-Home-and-Community-for___.YzJlOmRpcmVrdG9yYXRmb3JtZWRpc2luc2tlcHJvZHVrdGVyOmM6bzplZDNmY2I5NTQ2MjExMTQ4YjVmMDQ0NjBkZDM5OTUwYzo3OmU0NWI6NWEzMDczYTU2NGM3M2E1Y2IwMzQ2NTk1OGFhZDJjNzdmMTQyODNiYmNiOTA3ZGMwYTg0NTJmODAzNDBmM2VkMDpwOlQ6Rg) |
| 71 | Living with Spina Bifida myelomeningocele as adults (The LifestyleApp – a health application for persons living with SCI ) | [https://www.diva-portal.org/smash/get/diva2:1662178/FULLTEXT01.pdf](https://protect.checkpoint.com/v2/r02/___https:/www.diva-portal.org/smash/get/diva2:1662178/FULLTEXT01.pdf___.YzJlOmRpcmVrdG9yYXRmb3JtZWRpc2luc2tlcHJvZHVrdGVyOmM6bzplZDNmY2I5NTQ2MjExMTQ4YjVmMDQ0NjBkZDM5OTUwYzo3OjI1MWU6ZDM0ZjlmMTg3MGVlOGQwMjkzYzQ2MzhkYTBkM2VlNTdmMjM5ZWYyM2M0YmQ1OTBjN2ZkYjRkNjcyZTJmYTMxYTpwOlQ6Rg) |
| 72 | Enhance Transition Care for Medically Complex Adolescents | [https://core.ac.uk/download/pdf/322851766.pdf](https://protect.checkpoint.com/v2/r02/___https:/core.ac.uk/download/pdf/322851766.pdf___.YzJlOmRpcmVrdG9yYXRmb3JtZWRpc2luc2tlcHJvZHVrdGVyOmM6bzplZDNmY2I5NTQ2MjExMTQ4YjVmMDQ0NjBkZDM5OTUwYzo3OjRlOTE6ODFmYWJmOWNmMDQzYjk3NTNhOWJiYWJkZGJlZmFhNTBlOTVkYTcwZjk1YzFhOTliYWNlOTM4Nzk4MDAxNmM0ZjpwOlQ6Rg) |
| 92 | Internet-Delivered Cognitive Behavioural Therapy for People with Mild Traumatic Brain Injury: A Feasibility Study | [https://www.proquest.com/docview/2787192943?pq-origsite=gscholar&fromopenview=true&sourcetype=Dissertations%20&%20Theses](https://protect.checkpoint.com/v2/r02/___https:/www.proquest.com/docview/2787192943?pq-origsite=gscholar&fromopenview=true&sourcetype=Dissertations%20&%20Theses___.YzJlOmRpcmVrdG9yYXRmb3JtZWRpc2luc2tlcHJvZHVrdGVyOmM6bzplZDNmY2I5NTQ2MjExMTQ4YjVmMDQ0NjBkZDM5OTUwYzo3OmU4NGE6YzgyMjMxZjEzZWRjZjVhYjM0Mjk1MGVmNTA3ZjBkYzk5MGNlYjcwN2FkMmNjZGZlMWFhOWFiZTU3YWY5NWI4ZTpwOlQ6Rg) |
| 101 | Developing a Multi-Component Mobile Health App to Provide Ongoing Support for Family Caregivers of People with Chronic Conditions and Disabilities | [https://d-scholarship.pitt.edu/47175/](https://protect.checkpoint.com/v2/r02/___https:/d-scholarship.pitt.edu/47175/___.YzJlOmRpcmVrdG9yYXRmb3JtZWRpc2luc2tlcHJvZHVrdGVyOmM6bzplZDNmY2I5NTQ2MjExMTQ4YjVmMDQ0NjBkZDM5OTUwYzo3Ojg1OGU6NDZjOWFmZjZhMGJkMzY2OTkzM2I4OWNlMzExOGNiOGQ3MjFiYjZmNmRlNGM2NzI0NDMyOTNhMzYxMTQzYjMxMTpwOlQ6Rg) |
| 102 | The Effect of the Interactive Mobile Health and Rehabilitation System on Health and Psychosocial Outcomes in Spinal Cord Injury: Randomized Controlled Trial | [https://www.jmir.org/2019/8/e14305/](https://protect.checkpoint.com/v2/r02/___https:/www.jmir.org/2019/8/e14305/___.YzJlOmRpcmVrdG9yYXRmb3JtZWRpc2luc2tlcHJvZHVrdGVyOmM6bzplZDNmY2I5NTQ2MjExMTQ4YjVmMDQ0NjBkZDM5OTUwYzo3OmY1MDA6OTA0MjllNTZiMDQxMmExY2JkMjcwMDg2YjAxMjk2N2JkMDUzNTFjYmQ0OWMzNWMzZTc4YWIwYjMzZWJhMTE3ZDpwOlQ6Rg) |
| 103 | mHealth applications for children and adolescents with neurological disabilities | [https://www.theseus.fi/handle/10024/490054](https://protect.checkpoint.com/v2/r02/___https:/www.theseus.fi/handle/10024/490054___.YzJlOmRpcmVrdG9yYXRmb3JtZWRpc2luc2tlcHJvZHVrdGVyOmM6bzplZDNmY2I5NTQ2MjExMTQ4YjVmMDQ0NjBkZDM5OTUwYzo3OmM3ZGE6M2I4YzZmYjNlMjVlMmFiZWRmYWRmYWE3ODM4Zjc0Y2MxOWNiYTk3ZGVlZWJiMTdkMzAyZDYzMzNjMzg2MTYxMDpwOlQ6Rg) |
| 104 | Assessment of Health Literacy and Self-reported Readiness for Transition to Adult Care Among Adolescents and Young Adults With Spina Bifida | [https://jamanetwork.com/journals/jamanetworkopen/fullarticle/2784544](https://protect.checkpoint.com/v2/r02/___https:/jamanetwork.com/journals/jamanetworkopen/fullarticle/2784544___.YzJlOmRpcmVrdG9yYXRmb3JtZWRpc2luc2tlcHJvZHVrdGVyOmM6bzplZDNmY2I5NTQ2MjExMTQ4YjVmMDQ0NjBkZDM5OTUwYzo3OjYxMDc6NTgyY2ZiMmYxY2ZhYTdhNTc0ZTIyMDI0MTkzMjc5N2Q1ZTM3MWQ5YWYwOWFmYTExYmI3NjBjOTExMjg3ZDY2YzpwOlQ6Rg) |
| 105 | Systematic Review of Mobile Health Applications in Rehabilitation | [https://www.sciencedirect.com/science/article/pii/S0003999318311754?casa_token=dVNEIlt423gAAAAA:4_clQwJIBQHEF5igN4choCGtOLy0VdpORTqLBOLP3QQDUiFMmt5TPjZnqquUoD-zozyVVDlcStg](https://protect.checkpoint.com/v2/r02/___https:/www.sciencedirect.com/science/article/pii/S0003999318311754?casa_token=dVNEIlt423gAAAAA:4_clQwJIBQHEF5igN4choCGtOLy0VdpORTqLBOLP3QQDUiFMmt5TPjZnqquUoD-zozyVVDlcStg___.YzJlOmRpcmVrdG9yYXRmb3JtZWRpc2luc2tlcHJvZHVrdGVyOmM6bzplZDNmY2I5NTQ2MjExMTQ4YjVmMDQ0NjBkZDM5OTUwYzo3OjVlYzE6NDllMDhmZmVhMGRhN2Y2NzUwMmZlZWEyNTRiYjc4ZWU5ZGJiM2ZhYzUwMzRkNDJlZTFlYjI1MjcxNzYyYzJjODpwOlQ6Rg) |
| 106 | Apps and Mobile Health Technology in Rehabilitation | [https://www.pmr.theclinics.com/article/S1047-9651(18)30865-9/abstract](https://protect.checkpoint.com/v2/r02/___https:/www.pmr.theclinics.com/article/S1047-9651(18)30865-9/abstract___.YzJlOmRpcmVrdG9yYXRmb3JtZWRpc2luc2tlcHJvZHVrdGVyOmM6bzplZDNmY2I5NTQ2MjExMTQ4YjVmMDQ0NjBkZDM5OTUwYzo3OmU1YmI6ZDcyMTllMDI2MTllMmY0MDdjZTY3NDRiZTBmZjkwOWQwOGE3NDEwYjE1YmFlY2ZkYTUwN2UyMjcwY2M4MmFiMTpwOlQ6Rg) |
| 107 | An Adaptive Mobile Health System to Support Self-Management for Persons With Chronic Conditions and Disabilities: Usability and Feasibility Studies | [https://formative.jmir.org/2019/2/e12982](https://protect.checkpoint.com/v2/r02/___https:/formative.jmir.org/2019/2/e12982___.YzJlOmRpcmVrdG9yYXRmb3JtZWRpc2luc2tlcHJvZHVrdGVyOmM6bzplZDNmY2I5NTQ2MjExMTQ4YjVmMDQ0NjBkZDM5OTUwYzo3OmIxNGI6MzZmNDhmODg2OTdjMDI3ZTVlNjY2MzYxNzY3ZmFmMTRhMzQ5MmU2Nzg0YWYxYjVkYTNjMjEzYThhNDdlMjk4MDpwOlQ6Rg) |
| 108 | Assistive Mobile Technologies for Health Monitoring and Brain–Computer Interface for Patients with Motor Impairments | [https://link.springer.com/chapter/10.1007/978-3-319-93491-4_11](https://protect.checkpoint.com/v2/r02/___https:/link.springer.com/chapter/10.1007/978-3-319-93491-4_11___.YzJlOmRpcmVrdG9yYXRmb3JtZWRpc2luc2tlcHJvZHVrdGVyOmM6bzplZDNmY2I5NTQ2MjExMTQ4YjVmMDQ0NjBkZDM5OTUwYzo3OjhhNGY6MWZmNTcwYzZiZjU1MTZkZDk5MTU1YThkMDlkYzllZGE5MWZjNGQ1NTE1NThjOWQ0NGM2ZmU4NjBkZjg4NmNiYTpwOlQ6Rg) |
| 109 | Reaching People With Disabilities in Underserved Areas Through Digital Interventions: Systematic Review | [https://www.jmir.org/2019/10/e12981/](https://protect.checkpoint.com/v2/r02/___https:/www.jmir.org/2019/10/e12981/___.YzJlOmRpcmVrdG9yYXRmb3JtZWRpc2luc2tlcHJvZHVrdGVyOmM6bzplZDNmY2I5NTQ2MjExMTQ4YjVmMDQ0NjBkZDM5OTUwYzo3OmMzMzY6ZjY1MDc2ZjI5NDk1NTMzNzAyYzA2MjUxYmRiY2MwZjY1YjNiNjdkMTgzZjJiYWNlZGU0OTQzMjRhZTlmYWRiZDpwOlQ6Rg) |
| 110 | Feasibility and Acceptability of a Codesigned Health Care Transition Intervention for Young People With Spinal Cord Injuries | [https://meridian.allenpress.com/tscir/article/29/3/89/494933](https://protect.checkpoint.com/v2/r02/___https:/meridian.allenpress.com/tscir/article/29/3/89/494933___.YzJlOmRpcmVrdG9yYXRmb3JtZWRpc2luc2tlcHJvZHVrdGVyOmM6bzplZDNmY2I5NTQ2MjExMTQ4YjVmMDQ0NjBkZDM5OTUwYzo3OmJmNjc6NmZjN2IwMjVmM2I0OTU5ZjFiZjc3Y2YxMTczY2FiMDQ4YzJjNWI4YTQ1YjI1NjFmN2NkZWY4NGE0YWNmOWJhYTpwOlQ6Rg) |
| 111 | An Integrated and Adaptable mHealth System to Support Individuals with Chronic Conditions and Disabilities: Development, Evaluation, and Exploration for Future Works | [https://www.proquest.com/docview/3122639973?pq-origsite=gscholar&fromopenview=true&sourcetype=Dissertations%20&%20Theses](https://protect.checkpoint.com/v2/r02/___https:/www.proquest.com/docview/3122639973?pq-origsite=gscholar&fromopenview=true&sourcetype=Dissertations%20&%20Theses___.YzJlOmRpcmVrdG9yYXRmb3JtZWRpc2luc2tlcHJvZHVrdGVyOmM6bzplZDNmY2I5NTQ2MjExMTQ4YjVmMDQ0NjBkZDM5OTUwYzo3OjU5YTM6ZjY5MTE3ZDcwNzZmNWI3NDZjYmI3MTgwNGY1MDlmMmE4ODMxY2RlZTVlNGVlNTQ5MzMyMDk0NGJkYTM3MDY0YTpwOlQ6Rg) |
| 112 | Virtual Reality-Based Exercise with Exergames as Medicine in Different Contexts: A Short Review | [https://pmc.ncbi.nlm.nih.gov/articles/PMC6407662/](https://protect.checkpoint.com/v2/r02/___https:/pmc.ncbi.nlm.nih.gov/articles/PMC6407662/___.YzJlOmRpcmVrdG9yYXRmb3JtZWRpc2luc2tlcHJvZHVrdGVyOmM6bzplZDNmY2I5NTQ2MjExMTQ4YjVmMDQ0NjBkZDM5OTUwYzo3OmYxNmY6MmMwZmVkZTE3NmNkYWQ0ODIxODk3NTNiYTc1NWNkMGM2Y2NmNGFhN2E4N2M5MTBiMDU1NGFmNTNiM2U5MDliYzpwOlQ6Rg) |
| 113 | Chapter 24 - Pediatric Telerehabilitation | [https://www.sciencedirect.com/science/article/abs/pii/B9780323824866000241](https://protect.checkpoint.com/v2/r02/___https:/www.sciencedirect.com/science/article/abs/pii/B9780323824866000241___.YzJlOmRpcmVrdG9yYXRmb3JtZWRpc2luc2tlcHJvZHVrdGVyOmM6bzplZDNmY2I5NTQ2MjExMTQ4YjVmMDQ0NjBkZDM5OTUwYzo3OjhkMzg6OTQ0ZmFiMDk2YjdjZWZlZDMyYmQ3NzBlNTMwMDE3NTQzMmZkZDcwYzFlNzcwYjkwZTI1ZDUxOGRhOGU2NzIzYjpwOlQ6Rg) |
| 114 | Mobile and web-based apps that support self-management and transition in young people with chronic illness: systematic review | [https://pubmed.ncbi.nlm.nih.gov/31746773/](https://protect.checkpoint.com/v2/___https:/pubmed.ncbi.nlm.nih.gov/31746773/___.YzJlOmRpcmVrdG9yYXRmb3JtZWRpc2luc2tlcHJvZHVrdGVyOmM6bzo4ZTBjN2I1Y2UxMWEwODc0NzMyYTk1NDg4MDYwYWUxNjo2OmIwMzc6YTA4YTZiYmI3ZTAxODAwMjYwYmQ0ZmIwZTdiNjgzZTYxODM3YjI0NzUwOTIyYjQyYjY2OTQyYTJmYTcyNWI5ODpwOlQ6Tg) |
| 115 | Development of MyREADY Transition BBD Mobile App, a Health Intervention Technology Platform, to Improve Care Transition for Youth With Brain-Based Disabilities: User-Centered Design Approach | https://pubmed.ncbi.nlm.nih.gov/39352737/ |
| 116 | Monitoring Educational Intervention Programs for Children and Young People with Disabilities through a Web Application | [https://www.mdpi.com/2227-7102/14/3/306](https://protect.checkpoint.com/v2/___https:/www.mdpi.com/2227-7102/14/3/306___.YzJlOmRpcmVrdG9yYXRmb3JtZWRpc2luc2tlcHJvZHVrdGVyOmM6bzo4ZTBjN2I1Y2UxMWEwODc0NzMyYTk1NDg4MDYwYWUxNjo2OjI0NjE6ZTk2OTRmYzAxNGFkYzRiNWZkOGE2MjlhMTM4NTk0N2Q4YmIzYTc0ZmRiZmJmZjkwNTQ2NWRkYmRiNTg0Mzg0ZDpwOlQ6Tg) |
| 117 | CHILD-BRIGHT READYorNot Brain-Based Disabilities Trial: protocol of a randomised controlled trial (RCT) investigating the effectiveness of a patient-facing e-health intervention designed to enhance healthcare transition readiness in youth | [https://pubmed.ncbi.nlm.nih.gov/33771833/](https://protect.checkpoint.com/v2/___https:/pubmed.ncbi.nlm.nih.gov/33771833/___.YzJlOmRpcmVrdG9yYXRmb3JtZWRpc2luc2tlcHJvZHVrdGVyOmM6bzo4ZTBjN2I1Y2UxMWEwODc0NzMyYTk1NDg4MDYwYWUxNjo2OjU1ODg6NmEyZDU4MGNkYzZiNzFiZDZlMzZlNjY0ODRiN2NmZTNiNjc5NDY0ZjUzNTBkMTkwZmMyNmQwZjI0OTkxMjEwYTpwOlQ6Tg) |
| 118 | The Effectiveness of Web-Based Interventions Delivered to Children and Young People With Neurodevelopmental Disorders: Systematic Review and Meta-Analysis | [https://pubmed.ncbi.nlm.nih.gov/31682573/](https://protect.checkpoint.com/v2/___https:/pubmed.ncbi.nlm.nih.gov/31682573/___.YzJlOmRpcmVrdG9yYXRmb3JtZWRpc2luc2tlcHJvZHVrdGVyOmM6bzo4ZTBjN2I1Y2UxMWEwODc0NzMyYTk1NDg4MDYwYWUxNjo2OjFlYjg6ZjJjZmE4MGE4YzNjMTU1OTM2NDlmZmZiNTliMzY5MjBlZjY4N2FjMzk4ZWI3MzcxNDQ1NWEwNmM0NjU0MjQxODpwOlQ6Tg) |
| 119 | Pilot study of a mobile application-based intervention to induce changes in neural activity in the frontal region and behaviors in children with attention deficit hyperactivity disorder and/or intellectual disability | [https://pubmed.ncbi.nlm.nih.gov/34785036/](https://protect.checkpoint.com/v2/___https:/pubmed.ncbi.nlm.nih.gov/34785036/___.YzJlOmRpcmVrdG9yYXRmb3JtZWRpc2luc2tlcHJvZHVrdGVyOmM6bzo4ZTBjN2I1Y2UxMWEwODc0NzMyYTk1NDg4MDYwYWUxNjo2OmY2MmE6ZDU5MDlmZjc4YzdiYjMwNDRhOGUyMjAzMWQ0Zjc0NjNmZjI1YzNjZTkwMzhmYjBiMGVjNTJiNzRkOTViZDc5ODpwOlQ6Tg) |
| 120 | The treatable intellectual disability APP www.treatable-id.org: A digital tool to enhance diagnosis & care for rare diseases | [https://ojrd.biomedcentral.com/articles/10.1186/1750-1172-7-47](https://protect.checkpoint.com/v2/___https:/ojrd.biomedcentral.com/articles/10.1186/1750-1172-7-47___.YzJlOmRpcmVrdG9yYXRmb3JtZWRpc2luc2tlcHJvZHVrdGVyOmM6bzo4ZTBjN2I1Y2UxMWEwODc0NzMyYTk1NDg4MDYwYWUxNjo2OjFmM2I6ODZlZDE1N2QwZDA3ZDE5ZGQzN2U1ZTAzYjliM2I5MTUwMzUyYzYwNzA1N2M5YWQ5M2E2ODBmNWQ0MTU4ZjlmMzpwOlQ6Tg) |
| 121 | mHealth applications for children and adolescents with neurological disabilities | [https://www.theseus.fi/handle/10024/490054](https://protect.checkpoint.com/v2/___https:/www.theseus.fi/handle/10024/490054___.YzJlOmRpcmVrdG9yYXRmb3JtZWRpc2luc2tlcHJvZHVrdGVyOmM6bzo4ZTBjN2I1Y2UxMWEwODc0NzMyYTk1NDg4MDYwYWUxNjo2OjkwYWE6MjcwMjVlNTk2YWQwOGM2NjEwZDgwZjQ2OWQwZjZkYjRmZjk4YjVlN2YxZTFjM2RmZjgyNjg3ZmEyOWQyNjNkNjpwOlQ6Tg) |
| 122 | User Experience (re)Design and Evaluation of a Self-Guided, Mobile Health App for Adolescents with Mild Traumatic Brain Injury | [https://link.springer.com/article/10.1007/s41686-019-00038-x](https://protect.checkpoint.com/v2/___https:/link.springer.com/article/10.1007/s41686-019-00038-x___.YzJlOmRpcmVrdG9yYXRmb3JtZWRpc2luc2tlcHJvZHVrdGVyOmM6bzo4ZTBjN2I1Y2UxMWEwODc0NzMyYTk1NDg4MDYwYWUxNjo2OmJhZWE6YTJhNDU3OWQ3OTk1NzdlMjAwNTc1ODQwNDBmYWMyNjc1YTNjNzQ1YzczZGJhZjBmOGM5OGNlZTExYWExNjU3ODpwOlQ6Tg) |
| 123 | Could virtual reality applications pose real risks to children and adolescents? A systematic review of ethical issues and concerns | [https://link.springer.com/article/10.1007/s10055-021-00563-w](https://protect.checkpoint.com/v2/___https:/link.springer.com/article/10.1007/s10055-021-00563-w___.YzJlOmRpcmVrdG9yYXRmb3JtZWRpc2luc2tlcHJvZHVrdGVyOmM6bzo4ZTBjN2I1Y2UxMWEwODc0NzMyYTk1NDg4MDYwYWUxNjo2OjgyMjc6ZTI4MGRiYTU5YTNlMTk5YzIzYmQzMTYzYjhlNjdiMmJmOWFiNWQyMDAwN2ZhOTRhMTMzMTkwNTZhYjQ1MGNhOTpwOlQ6Tg) |
| 124 | Annual Research Review: Digital health interventions for children and young people with mental health problems – a systematic and meta-review | [https://acamh.onlinelibrary.wiley.com/doi/full/10.1111/jcpp.12663?casa_token=oMXe5NkaPBAAAAAA%3A3jwfNgh3jAbkPu-Vi3QYxSkQQEkMQndcCW7QKj7rkhIv3a34A3ghpcKWRQxvBznw_24DsN5IQuDFF0AN](https://protect.checkpoint.com/v2/___https:/acamh.onlinelibrary.wiley.com/doi/full/10.1111/jcpp.12663?casa_token=oMXe5NkaPBAAAAAA%3A3jwfNgh3jAbkPu-Vi3QYxSkQQEkMQndcCW7QKj7rkhIv3a34A3ghpcKWRQxvBznw_24DsN5IQuDFF0AN___.YzJlOmRpcmVrdG9yYXRmb3JtZWRpc2luc2tlcHJvZHVrdGVyOmM6bzo4ZTBjN2I1Y2UxMWEwODc0NzMyYTk1NDg4MDYwYWUxNjo2OmE4YmQ6NGRhYjM4ODJmM2NhODBlMzhhYTQwOWQ1ZWYyNDFkMzIyNmU0ZmZkZjk2ZGJkOWEyNmIxZTBiNTk0OTliYjcyODpwOlQ6Tg) |
| 125 | Exploring Digital-based Therapies for Adolescents with Substance Use Disorders: A Brief Literature Review | [https://journals.sagepub.com/doi/full/10.1177/09731342241248896](https://protect.checkpoint.com/v2/___https:/journals.sagepub.com/doi/full/10.1177/09731342241248896___.YzJlOmRpcmVrdG9yYXRmb3JtZWRpc2luc2tlcHJvZHVrdGVyOmM6bzo4ZTBjN2I1Y2UxMWEwODc0NzMyYTk1NDg4MDYwYWUxNjo2OmZkZmQ6OTE3NjdlNmFlNzA1YTFmYmY4MGI1M2FkYTQ2ZjZmODMwZTU3MGRhYTg1NjgyN2U1YmNlNmRmODg3ZDIxMTYwZTpwOlQ6Tg) |
| 126 | Attention-deficit/ hyperactivity disorder mobile apps: A systematic review | [https://www.sciencedirect.com/science/article/pii/S138650561830323X?casa_token=vWvxwX6_U5MAAAAA:GMCErd38lfpeofTqJ3c9gZh14GD-bf6QAfRnEWPgy4mOe5VfN64X5J-yL50ajVuidAdqP_LBq08](https://protect.checkpoint.com/v2/___https:/www.sciencedirect.com/science/article/pii/S138650561830323X?casa_token=vWvxwX6_U5MAAAAA:GMCErd38lfpeofTqJ3c9gZh14GD-bf6QAfRnEWPgy4mOe5VfN64X5J-yL50ajVuidAdqP_LBq08___.YzJlOmRpcmVrdG9yYXRmb3JtZWRpc2luc2tlcHJvZHVrdGVyOmM6bzo4ZTBjN2I1Y2UxMWEwODc0NzMyYTk1NDg4MDYwYWUxNjo2OmRlYTM6ZDM0ZmFjZmM1OWZkOGVhNmFkZjdkYTNhZmI4NzFiM2IwNjBjZmJhM2NmMzIxMzI3MzZlNzI1YWFjYjhlZTVlMTpwOlQ6Tg) |
| 127 | Apps and Digital Resources for Child Neurodevelopment, Mental Health, and Well-Being: Review, Evaluation, and Reflection on Current Resources | [https://www.jmir.org/2025/1/e58693/](https://protect.checkpoint.com/v2/___https:/www.jmir.org/2025/1/e58693/___.YzJlOmRpcmVrdG9yYXRmb3JtZWRpc2luc2tlcHJvZHVrdGVyOmM6bzo4ZTBjN2I1Y2UxMWEwODc0NzMyYTk1NDg4MDYwYWUxNjo2OmMyYTk6MmNjOTEzMTI4Y2EyOTFlZDBiOTNhZjk0NDU4MDgzOWViMmRmZjQ1MmQ5NGY0YjZmMzkyZTY0ZmMwNGEyNzA5NDpwOlQ6Tg) |
| 128 | Navigating meaningful engagement: lessons from partnering with youth and families in brain-based disability research | [https://link.springer.com/article/10.1186/s40900-024-00543-9](https://protect.checkpoint.com/v2/___https:/link.springer.com/article/10.1186/s40900-024-00543-9___.YzJlOmRpcmVrdG9yYXRmb3JtZWRpc2luc2tlcHJvZHVrdGVyOmM6bzo4ZTBjN2I1Y2UxMWEwODc0NzMyYTk1NDg4MDYwYWUxNjo2OjA5ZDk6ZWIzMTZkMTRlNWRhYTdjM2FmMzJmMDdiNTFkMjIzNWFlZGFlMzI2Y2U0NTM4Y2NjMzc2Y2U0YjI3MTc3Y2QzODpwOlQ6Tg) |
| 129 | A Multidisciplinary Telerehabilitation Approach for Supporting Social Interaction in Autism Spectrum Disorder Families: An Italian Digital Platform in Response to COVID-19 | [https://www.mdpi.com/2076-3425/11/11/1404](https://protect.checkpoint.com/v2/___https:/www.mdpi.com/2076-3425/11/11/1404___.YzJlOmRpcmVrdG9yYXRmb3JtZWRpc2luc2tlcHJvZHVrdGVyOmM6bzo4ZTBjN2I1Y2UxMWEwODc0NzMyYTk1NDg4MDYwYWUxNjo2OmZmZTk6N2U5M2FhOGU5Njc2ZWFiY2Q2YWJhMWFjNzUwYzY4ZWRiZWE3NTBiZjZhOWFkMDM1Njc3ZWQ1MTYzZDU0NWQxNDpwOlQ6Tg) |
| 130 | Ecological Momentary Assessment and Mood Disorders in Children and Adolescents: a Systematic Review | [https://link.springer.com/article/10.1007/s11920-018-0913-z](https://protect.checkpoint.com/v2/___https:/link.springer.com/article/10.1007/s11920-018-0913-z___.YzJlOmRpcmVrdG9yYXRmb3JtZWRpc2luc2tlcHJvZHVrdGVyOmM6bzo4ZTBjN2I1Y2UxMWEwODc0NzMyYTk1NDg4MDYwYWUxNjo2OjJmZjY6NzhjNWJlMmM0ZjMyY2MzOTVlYzdmMzlmYjBhNWRiZTRlMTJhZTFiZjEzMjc2ZDIxMTIyZDMwMmRhY2EwZWNlYzpwOlQ6Tg) |
| 131 | Understanding Digital Dementia and Cognitive Impact in the Current Era of the Internet: A Review | [https://assets.cureus.com/uploads/review_article/pdf/278251/20241024-468093-avv35f.pdf](https://protect.checkpoint.com/v2/___https:/assets.cureus.com/uploads/review_article/pdf/278251/20241024-468093-avv35f.pdf___.YzJlOmRpcmVrdG9yYXRmb3JtZWRpc2luc2tlcHJvZHVrdGVyOmM6bzo4ZTBjN2I1Y2UxMWEwODc0NzMyYTk1NDg4MDYwYWUxNjo2OjkyNTY6NjkyYWI1NjIzZDNmYzhmNDU1NDk4NTIyZmVmYzkzZjA4YmNiNGJiZjYzZDM4YWQxMWQ4MjFlNjQ1OTA0MDA5NDpwOlQ6Tg) |
| 132 | Digital Addiction Intervention for Children and Adolescents: A Scoping Review | [https://www.mdpi.com/1660-4601/20/6/4777](https://protect.checkpoint.com/v2/___https:/www.mdpi.com/1660-4601/20/6/4777___.YzJlOmRpcmVrdG9yYXRmb3JtZWRpc2luc2tlcHJvZHVrdGVyOmM6bzo4ZTBjN2I1Y2UxMWEwODc0NzMyYTk1NDg4MDYwYWUxNjo2OjhjMGU6ZWViZDFmYjQ5ZjcwZDA2YzMwMTNhMTA0NzRmZDY3YjkwOGFhODBmOGViY2E5OTEzYWEzYTRmYTU4YzEyNGQwNDpwOlQ6Tg) |
| 133 | Remote Digital Psychiatry for Mobile Mental Health Assessment and Therapy: MindLogger Platform Development Study | [https://www.jmir.org/2021/11/e22369/](https://protect.checkpoint.com/v2/___https:/www.jmir.org/2021/11/e22369/___.YzJlOmRpcmVrdG9yYXRmb3JtZWRpc2luc2tlcHJvZHVrdGVyOmM6bzo4ZTBjN2I1Y2UxMWEwODc0NzMyYTk1NDg4MDYwYWUxNjo2OjlhY2E6YWFlOGZiOTU0MThkOTYzYTRjZTc2ZjVhYjMxNzc5NjRlZDZmZmQzOGUxMDU1ZWVlMzQ4M2JkZTA5NjRjNjA4NzpwOlQ6Tg) |
| 134 | Smartphone App–Delivered Mindfulness-Based Intervention for Mild Traumatic Brain Injury in Adolescents: Protocol for a Feasibility Randomized Controlled Trial | [https://www.researchprotocols.org/2024/1/e57226/](https://protect.checkpoint.com/v2/___https:/www.researchprotocols.org/2024/1/e57226/___.YzJlOmRpcmVrdG9yYXRmb3JtZWRpc2luc2tlcHJvZHVrdGVyOmM6bzo4ZTBjN2I1Y2UxMWEwODc0NzMyYTk1NDg4MDYwYWUxNjo2OjRkZmM6MGQ0OTdlZmZjMTFjYjljOTBjMzQzYTEzMDUxZmQwYzM1M2RjYWE4YzQ5YjJjNTUzMjE0MDliNzQxYzBhMGEyMDpwOlQ6Tg) |
| 135 | Mobile Health for Traumatic Brain Injury: A Systematic Review of the Literature and Mobile Application Market | [https://assets.cureus.com/uploads/review_article/pdf/19916/1612428831-1612428827-20210204-18204-wegguk.pdf](https://protect.checkpoint.com/v2/___https:/assets.cureus.com/uploads/review_article/pdf/19916/1612428831-1612428827-20210204-18204-wegguk.pdf___.YzJlOmRpcmVrdG9yYXRmb3JtZWRpc2luc2tlcHJvZHVrdGVyOmM6bzo4ZTBjN2I1Y2UxMWEwODc0NzMyYTk1NDg4MDYwYWUxNjo2OjRlM2M6ZjIyZDJiNGNhYTU2ODY5NTc5OTVjNGUzMzdjYzg2ODYzMzVmMzljYTcyY2Q1NTc0ODNlYzBmY2Q1OWJiODJhMjpwOlQ6Tg) |
| 136 | A Mobile Phone App for the Self-Management of Pediatric Concussion: Development and Usability Testing | [https://humanfactors.jmir.org/2019/2/e12135/](https://protect.checkpoint.com/v2/___https:/humanfactors.jmir.org/2019/2/e12135/___.YzJlOmRpcmVrdG9yYXRmb3JtZWRpc2luc2tlcHJvZHVrdGVyOmM6bzo4ZTBjN2I1Y2UxMWEwODc0NzMyYTk1NDg4MDYwYWUxNjo2OmVhMTg6ZTkzMjdiOGVhZDUwOTdkZDZiMGM0NzBmMGVjMzFkNDFjMDFiNjM5OGRjYWFjYmIxOGRjYjJmZjkyZDE0OWNlYTpwOlQ6Tg) |
| 137 | Practical applications for use of PDAs and smartphones with children and adolescents who have traumatic brain injury | [https://journals.sagepub.com/doi/abs/10.3233/NRE-2008-23605](https://protect.checkpoint.com/v2/___https:/journals.sagepub.com/doi/abs/10.3233/NRE-2008-23605___.YzJlOmRpcmVrdG9yYXRmb3JtZWRpc2luc2tlcHJvZHVrdGVyOmM6bzo4ZTBjN2I1Y2UxMWEwODc0NzMyYTk1NDg4MDYwYWUxNjo2OmQ0YzU6OTBiN2FhZWVkY2ZhYTgwN2U1MjI2MTkwYWQwNGRmNmYwNzhmYzM1ZDk1NzY2MDQyZmRlNTJhMGJhYjIxMWVkMTpwOlQ6Tg) |
| 138 | Reducing concussion symptoms among teenage youth: Evaluation of a mobile health app | [https://www.tandfonline.com/doi/full/10.1080/02699052.2017.1332388?casa_token=ZFXitf8BnysAAAAA%3AdBD1pw6yZpdAuQ7UJsxELEyaPIsriOa5lT1xCW2Zjgfli5QrccFm8Qj2J7YQb9G8op9IXYJkT7ENkA](https://protect.checkpoint.com/v2/___https:/www.tandfonline.com/doi/full/10.1080/02699052.2017.1332388?casa_token=ZFXitf8BnysAAAAA%3AdBD1pw6yZpdAuQ7UJsxELEyaPIsriOa5lT1xCW2Zjgfli5QrccFm8Qj2J7YQb9G8op9IXYJkT7ENkA___.YzJlOmRpcmVrdG9yYXRmb3JtZWRpc2luc2tlcHJvZHVrdGVyOmM6bzo4ZTBjN2I1Y2UxMWEwODc0NzMyYTk1NDg4MDYwYWUxNjo2OmJkZTE6YjMwY2JlMGU4Zjc0NTBlZGJlMzRhYWViNDhlNGVhOGRjZjZkYmNmZDJiOGY1OGI2MDZhZGRjYzUyOWU3ZWE5ODpwOlQ6Tg) |
| 139 | The Use of a Mobile Application to Teach Concussion-Related Health Knowledge | [https://pmc.ncbi.nlm.nih.gov/articles/PMC8133699/](https://protect.checkpoint.com/v2/___https:/pmc.ncbi.nlm.nih.gov/articles/PMC8133699/___.YzJlOmRpcmVrdG9yYXRmb3JtZWRpc2luc2tlcHJvZHVrdGVyOmM6bzo4ZTBjN2I1Y2UxMWEwODc0NzMyYTk1NDg4MDYwYWUxNjo2OmIxZjM6MTcyNmY1MWI3YTZhOThiNTllNzEwMDk1YTMzNzBlOWQ2MGQxYTljYjY1NWViYWU1MGZhOTRlMThiYjcwZTNhNDpwOlQ6Tg) |
| 140 | Informing design of an app-based coaching intervention to promote social participation of teenagers with traumatic brain injury | [https://www.tandfonline.com/doi/full/10.1080/17518423.2016.1237584?casa_token=WFchna1Gkx0AAAAA%3AcB6ZMCzowabuS-3g7kiIKJixmvIb1CswB5RAEYv89jQRX51l_XH96u82wPNcNPXomYaM51ai2NxDHg](https://protect.checkpoint.com/v2/___https:/www.tandfonline.com/doi/full/10.1080/17518423.2016.1237584?casa_token=WFchna1Gkx0AAAAA%3AcB6ZMCzowabuS-3g7kiIKJixmvIb1CswB5RAEYv89jQRX51l_XH96u82wPNcNPXomYaM51ai2NxDHg___.YzJlOmRpcmVrdG9yYXRmb3JtZWRpc2luc2tlcHJvZHVrdGVyOmM6bzo4ZTBjN2I1Y2UxMWEwODc0NzMyYTk1NDg4MDYwYWUxNjo2OmQwZmY6NzJjOGJlNzU3MzgyNzMyY2M3OTI4YzgzZTE3Mjg3MmQzNjFiYTdmMWU0OGNkNTQwYjZkMjRkMmE0ZDg3ZDFiYzpwOlQ6Tg) |
| 141 | Games for Health for Children—Current Status and Needed Research | [https://www.liebertpub.com/doi/abs/10.1089/g4h.2015.0026](https://protect.checkpoint.com/v2/___https:/www.liebertpub.com/doi/abs/10.1089/g4h.2015.0026___.YzJlOmRpcmVrdG9yYXRmb3JtZWRpc2luc2tlcHJvZHVrdGVyOmM6bzo4ZTBjN2I1Y2UxMWEwODc0NzMyYTk1NDg4MDYwYWUxNjo2OjRmN2E6YjQyYzc3YzU0ZTkyYmEyNTIyNTY3Y2E3ZTcyMzA4MGUwZjgzYWVjYTE2N2M0YTkyYjU5MGIyMWFjMDAxMDcwYTpwOlQ6Tg) |
| 142 | Mobile medical and health apps: state of the art, concerns, regulatory control and certification | [https://ojphi.jmir.org/2013/3/e61275/PDF](https://protect.checkpoint.com/v2/___https:/ojphi.jmir.org/2013/3/e61275/PDF___.YzJlOmRpcmVrdG9yYXRmb3JtZWRpc2luc2tlcHJvZHVrdGVyOmM6bzo4ZTBjN2I1Y2UxMWEwODc0NzMyYTk1NDg4MDYwYWUxNjo2OjZjNGU6NWU0MzE0NWQyZmJlODE4NWY5YjQ1Zjk1NDUzNjY0M2EzYTk4ZGRlMmJlMTI3YTZiZjdiMzRjN2RiMGM3N2MxYzpwOlQ6Tg) |
| 143 | Online videos promote brain health literacy | [https://academic.oup.com/heapro/article-abstract/36/5/1243/6056681](https://protect.checkpoint.com/v2/___https:/academic.oup.com/heapro/article-abstract/36/5/1243/6056681___.YzJlOmRpcmVrdG9yYXRmb3JtZWRpc2luc2tlcHJvZHVrdGVyOmM6bzo4ZTBjN2I1Y2UxMWEwODc0NzMyYTk1NDg4MDYwYWUxNjo2OmMyZmI6NTk1ZTFkNGIyZjY3ZmFmY2ZmZjAyOWM1MDU3M2VjNGI5OGNjMDQ2YTg3N2MzZDBiNmE1MmQ1N2E1YzQ4ODA4ZDpwOlQ6Tg) |
| 144 | Gaming Technology for Pediatric Neurorehabilitation: A Systematic Review | [https://www.frontiersin.org/journals/pediatrics/articles/10.3389/fped.2022.775356/full](https://protect.checkpoint.com/v2/___https:/www.frontiersin.org/journals/pediatrics/articles/10.3389/fped.2022.775356/full___.YzJlOmRpcmVrdG9yYXRmb3JtZWRpc2luc2tlcHJvZHVrdGVyOmM6bzo4ZTBjN2I1Y2UxMWEwODc0NzMyYTk1NDg4MDYwYWUxNjo2Ojc0ZDI6OWVmZmVkNWU2MDJlMTc4NTA4N2ZiYThkNWRmYjkxYWNlZGIyZjYyMzc3MTc5ODRlY2E2NGExOWQ0YWIzNWI5NjpwOlQ6Tg) |
| 145 | Serious games for improving knowledge and self-management in young people with chronic conditions: a systematic review and meta-analysis | [https://academic.oup.com/jamia/article/23/1/230/2380055](https://protect.checkpoint.com/v2/___https:/academic.oup.com/jamia/article/23/1/230/2380055___.YzJlOmRpcmVrdG9yYXRmb3JtZWRpc2luc2tlcHJvZHVrdGVyOmM6bzo4ZTBjN2I1Y2UxMWEwODc0NzMyYTk1NDg4MDYwYWUxNjo2OjMwMTU6OTg4MWFiMjRhODU1NGU4NzA5MjNiNTgwZWYwODYwYTg0OGMwODE5ZTEzN2QxNzVhODUwMjYxNDdkZDM2NTJmNTpwOlQ6Tg) |
| 146 | Feasibility of a Commercially Available Virtual Reality System to Achieve Exercise Guidelines in Youth With Spina Bifida: Mixed Methods Case Study | [https://games.jmir.org/2020/3/e20667](https://protect.checkpoint.com/v2/___https:/games.jmir.org/2020/3/e20667___.YzJlOmRpcmVrdG9yYXRmb3JtZWRpc2luc2tlcHJvZHVrdGVyOmM6bzo4ZTBjN2I1Y2UxMWEwODc0NzMyYTk1NDg4MDYwYWUxNjo2OjJiNzg6ZjUwNmI4NjRiZDg4M2ExNTk1YWRhY2RkNjc5YjhjZjE3ZDJmYmZmN2VhYjY3ZWQ0NWViYTkyYzdhOTRlYTM2MDpwOlQ6Tg) |
| 147 | Augmented Reality for Learning of Children and Adolescents With Autism Spectrum Disorder (ASD): A Systematic Review | [https://ieeexplore.ieee.org/abstract/document/9060971](https://protect.checkpoint.com/v2/___https:/ieeexplore.ieee.org/abstract/document/9060971___.YzJlOmRpcmVrdG9yYXRmb3JtZWRpc2luc2tlcHJvZHVrdGVyOmM6bzo4ZTBjN2I1Y2UxMWEwODc0NzMyYTk1NDg4MDYwYWUxNjo2OjU0ZjE6Y2MzMzI4NWYzYTk3Y2QwYjcyNjgyNTkxYzZkMjY1YTU0OTYzYzg1ZjI3N2U2MmE2Njg0MDgxOTBhYTMzMDg0ZjpwOlQ6Tg) |
| 148 | Impact of Digital Innovations on Health Literacy Applied to Patients with Special Needs: A Systematic Review | [https://www.mdpi.com/2078-2489/15/11/663](https://protect.checkpoint.com/v2/___https:/www.mdpi.com/2078-2489/15/11/663___.YzJlOmRpcmVrdG9yYXRmb3JtZWRpc2luc2tlcHJvZHVrdGVyOmM6bzo4ZTBjN2I1Y2UxMWEwODc0NzMyYTk1NDg4MDYwYWUxNjo2OjNlYzM6NTNmZWNiZmY0MGU5NjM5ZmI0MzA5NTg0YzhmYTcwZmUxNGE3OWUzMjFhMWI1NWZkZWUyYjIyY2ZjYzVlMTdkOTpwOlQ6Tg) |
| 149 | Feasibility of Using Mobile Health to Promote Self-Management in Spina Bifida | [https://journals.lww.com/ajpmr/abstract/2016/06000/feasibility_of_using_mobile_health_to_promote.4.aspx](https://protect.checkpoint.com/v2/___https:/journals.lww.com/ajpmr/abstract/2016/06000/feasibility_of_using_mobile_health_to_promote.4.aspx___.YzJlOmRpcmVrdG9yYXRmb3JtZWRpc2luc2tlcHJvZHVrdGVyOmM6bzo4ZTBjN2I1Y2UxMWEwODc0NzMyYTk1NDg4MDYwYWUxNjo2OmZhYWQ6YTBjOWI2OGQ0YjQ2YTA4NWY1MTgwNTQ0MjlhZjExNDUwNTA3YzIzZGU1ZjRhNjY0OTk2MzA2NWQ0NWRiZjY3MzpwOlQ6Tg) |
| 150 | Exploring an Existing Weight Management App for Use With Adolescents and Young Adults With Spina Bifida: Usability Study | [https://pediatrics.jmir.org/2019/2/e15153/](https://protect.checkpoint.com/v2/___https:/pediatrics.jmir.org/2019/2/e15153/___.YzJlOmRpcmVrdG9yYXRmb3JtZWRpc2luc2tlcHJvZHVrdGVyOmM6bzo4ZTBjN2I1Y2UxMWEwODc0NzMyYTk1NDg4MDYwYWUxNjo2OjI3MTg6Y2EzMDUzY2E4Mjg1MTAwMWMzYjBjYThkNWEwMTJhNjI2ZGVkOTZkMGFhYzUzMDhmYWQ0ZWMzZGZmNmZkMzY5MTpwOlQ6Tg) |
| 151 | Development of a Self-Management App for People with Spinal Cord Injury | [https://link.springer.com/article/10.1007/s10916-019-1273-x](https://protect.checkpoint.com/v2/___https:/link.springer.com/article/10.1007/s10916-019-1273-x___.YzJlOmRpcmVrdG9yYXRmb3JtZWRpc2luc2tlcHJvZHVrdGVyOmM6bzo4ZTBjN2I1Y2UxMWEwODc0NzMyYTk1NDg4MDYwYWUxNjo2OmJlMzE6YmQ1ZTYyMjhjMGYwNTA0Y2UyYjgyY2ZlZDI2M2MwZjE1MGJjMmYyMmMzNGI2NTg5ZTI2MWEyOWRjNDE2NzMyODpwOlQ6Tg) |
| 152 | Patients’ Perspectives on the Usability of a Mobile App for Self-Management following Spinal Cord Injury | [https://link.springer.com/article/10.1007/s10916-019-1487-y?fromPaywallRec=false](https://protect.checkpoint.com/v2/___https:/link.springer.com/article/10.1007/s10916-019-1487-y?fromPaywallRec=false___.YzJlOmRpcmVrdG9yYXRmb3JtZWRpc2luc2tlcHJvZHVrdGVyOmM6bzo4ZTBjN2I1Y2UxMWEwODc0NzMyYTk1NDg4MDYwYWUxNjo2OjY2MjU6MTU2MzFmN2ViZTlhZTllMDQ3NmVhNTU0ZjMyMDJiN2ZmYzg3NTIyZTc3YmY2OWU5ZmU2YTk5NTAyMmRmNmFiOTpwOlQ6Tg) |

## Example of Google searches

Google strings were simplified to “apps about [disease OR disease] and [children OR youth]. “

| **Condition** | **Name** | **Lin** |
| --- | --- | --- |
| Brain Injury | My Brain and Me | <https://braininjurygroup.co.uk/news/theres-app-young-people-affected-brain-injury/> |
|  | CanPlan | <https://www.thechildrenstrust.org.uk/brain-injury-information/real-stories/hobbies-apps> |
|  | My Medical | <https://www.thechildrenstrust.org.uk/brain-injury-information/real-stories/hobbies-apps> |
|  | Cozi Family Organizer | <https://www.thechildrenstrust.org.uk/brain-injury-information/real-stories/hobbies-apps> |
|  | ChoiceWorks | <https://www.thechildrenstrust.org.uk/brain-injury-information/real-stories/hobbies-apps> |
|  | Dexteria | <https://www.thechildrenstrust.org.uk/brain-injury-information/real-stories/hobbies-apps> |
|  | Injini: Child Development Game Suit | <https://www.thechildrenstrust.org.uk/brain-injury-information/real-stories/hobbies-apps> |
|  | iBookds with VoiceOVer | <https://www.thechildrenstrust.org.uk/brain-injury-information/real-stories/hobbies-apps> |
|  | GE MIND App | <https://www.thechildrenstrust.org.uk/brain-injury-information/real-stories/hobbies-apps> |
|  | Space Retrieval Therapy | <https://www.thechildrenstrust.org.uk/brain-injury-information/real-stories/hobbies-apps> |
|  | Lumosity: Brain Training | <https://www.thechildrenstrust.org.uk/brain-injury-information/real-stories/hobbies-apps> |
|  | Qcard: Outsmart Forgetfulness | <https://www.thechildrenstrust.org.uk/brain-injury-information/real-stories/hobbies-apps> |
|  | SmallTalk Aphasia | <https://www.thechildrenstrust.org.uk/brain-injury-information/real-stories/hobbies-apps> |
|  | Answers Yes no HD | <https://www.thechildrenstrust.org.uk/brain-injury-information/real-stories/hobbies-apps> |
|  | D2u Transcription | <https://www.thechildrenstrust.org.uk/brain-injury-information/real-stories/hobbies-apps> |
|  | QR Reader, using "if i need help customs ID cards | <https://www.thechildrenstrust.org.uk/brain-injury-information/real-stories/hobbies-apps> |
|  | Naming therapy | <https://www.thechildrenstrust.org.uk/brain-injury-information/real-stories/hobbies-apps> |
| Concussion | SupperBetter | <https://wexnermedical.osu.edu/our-stories/concussion-gaming-app> |
|  | Back2Play App | <https://www.canchild.ca/en/research-in-practice/current-studies/the-back2play-app-study> |
|  | NeuroCare | <https://pmc.ncbi.nlm.nih.gov/articles/PMC6658289/> |
| Neuro Rehab | Peak | <https://www.matrixneurological.org/what-we-do/neuro-rehabilitation/apps-to-aid-neuro-rehabilitation/> |
|  | Lumosity | https://www.matrixneurological.org/what-we-do/neuro-rehabilitation/apps-to-aid-neuro-rehabilitation/ |
|  | Elevate | https://www.matrixneurological.org/what-we-do/neuro-rehabilitation/apps-to-aid-neuro-rehabilitation/ |
| Chronic conditions | Pitt+Me | <https://pittplusme.org/studyarms/publicdetails?guid=840520cf-b25e-4a4d-9887-789a43a79bcf> |
| Spinal Cord | Managing an Intermittent Catheter | <https://neupsykey.com/mobile-health-apps-and-self-management-for-spinal-cord-injury-rehabilitation/> |
|  | PHIRE app |  |
|  | Amman et al (pressure injuries) |  |
|  | SCI Health storylines |  |
|  | iMHere |  |
|  | SCI Step Together mobile app | https://apps.  apple.com/ca/app/curatio-stronger-together/id1517935369 https://cdpp.ca/sites/default/files/Step%20Together%20Module%202/index.pdf |
|  | SCI health toolkit | <https://www.medicalrepublic.com.au/digital-tool-for-spinal-cord-injury/17915> |

| **Condition** | **Name** | **Lin** |
| --- | --- | --- |
| Spina Bifida | SBA | <https://apps.apple.com/us/app/spina-bifida-association/id1492488550> |
|  | SpinApp - ASBIHP | <https://play.google.com/store/apps/details?id=com.asbihp.app&hl=en> |
|  | Set Brave Goals app | [https://www.rileychildrens.org/research/featured-research/continence-goal-setting-app-enables-more-personalized-treatment-approach-for-spina-bifida https://pubmed.ncbi.nlm.nih.gov/38969555/](https://www.rileychildrens.org/research/featured-research/continence-goal-setting-app-enables-more-personalized-treatment-approach-for-spina-bifida) |
|  | iMHere | <https://economictimes.indiatimes.com/magazines/panache/new-smartphone-app-to-help-spina-bifida-patients/articleshow/49515406.cms?from=mdr> |
|  | PTPal™ Mobile Health App | <https://publications.aap.org/pediatrics/article/149/1%20Meeting%20Abstracts%20February%202022/216/186058/The-Development-of-a-Structured-Exercise-Program?autologincheck=redirected> |
|  | PACER Center Mobile App |  |

| **Condition** | **Name** | **Lin** |
| --- | --- | --- |
| Hydrocephalus | Hibi | <https://harrys-hat.org/get-help/helpful-hydro-apps/> |
|  | HC & Me | https://harrys-hat.org/get-help/helpful-hydro-apps/ |
|  | HydroAssist | https://harrys-hat.org/get-help/helpful-hydro-apps/ |
|  | MyREADY Transition BBD Mobile | <https://pediatrics.jmir.org/2024/1/e51606?__hstc=102212634.b8259356fcccdd9cfcb617fbd39b1ac4.1730851200214.1730851200215.1730851200216.1&__hssc=102212634.1.1730851200217&__hsfp=1169088524> |
