# Supplementary material for: Mobile Apps to Enhance Health Literacy in Children and Adolescents With Injury or Disease to the Brain or Spinal Cord: Scoping Review
Source: JMIR Pediatr Parent. 2026 Nov 13;9:e101750. doi: 10.2196/101750 (PMC13762682; doi:10.2196/101750)
Supplement: Supplementary file 5 [file pediatrics-v9-e101750-s004.docx]

**Multimedia Appendix 4. Searches in organizational websites.**

|  | **Organization** | **Website/link** | **Comments** |
| --- | --- | --- | --- |
| 1 | CanChild | [CanChild](https://protect.checkpoint.com/v2/r02/___https:/www.canchild.ca/___.YzJlOmRpcmVrdG9yYXRmb3JtZWRpc2luc2tlcHJvZHVrdGVyOmM6bzplZDNmY2I5NTQ2MjExMTQ4YjVmMDQ0NjBkZDM5OTUwYzo3OmFjNDU6Nzk1MjEyNDcxNTg3NWQxODcwYTJjZmJjMGQ5MzMwMzExNjdkMWYyMDQ5MGJhNjljNWMyMjc5Y2JjN2UyMDdhMjpwOlQ6Rg) | Information, webinars, videos, no interactive tools |
| 2 | SaskAbilities | [Home - SaskAbilities](https://protect.checkpoint.com/v2/r02/___https:/www.saskabilities.ca/___.YzJlOmRpcmVrdG9yYXRmb3JtZWRpc2luc2tlcHJvZHVrdGVyOmM6bzplZDNmY2I5NTQ2MjExMTQ4YjVmMDQ0NjBkZDM5OTUwYzo3OjE3Yjg6YzFmOGQ2OGM2ZGMxMjY3ODkyNTA2N2M3NmE4MGRiNTY0OGU4ODc5MWM3MGE4YmY5ZTEyMWJiODQyOWZlYzUwNzpwOlQ6Rg) | No hit |
| 3 | Kids brain health network | [https://kidsbrainhealth.ca/](https://protect.checkpoint.com/v2/r02/___https:/kidsbrainhealth.ca/___.YzJlOmRpcmVrdG9yYXRmb3JtZWRpc2luc2tlcHJvZHVrdGVyOmM6bzplZDNmY2I5NTQ2MjExMTQ4YjVmMDQ0NjBkZDM5OTUwYzo3OjgzNmU6YjQ4NGZhMzY1MmJlY2YxOGZmYTAxZjI1YTI1ZjlmNmYwMDM0ZDc3ZGQ1NTA0MzJiNDM3ZTc0M2YwZmYzY2IzZDpwOlQ6Rg) | No hit |
| 4 | Child Neurology foundation | [Child Neurology Foundation](https://protect.checkpoint.com/v2/r02/___https:/www.childneurologyfoundation.org/___.YzJlOmRpcmVrdG9yYXRmb3JtZWRpc2luc2tlcHJvZHVrdGVyOmM6bzplZDNmY2I5NTQ2MjExMTQ4YjVmMDQ0NjBkZDM5OTUwYzo3OjY1NDQ6ODQxNzljYWQ2NDdiYzkwYzdiN2I1ZGFmYjc4NGM3ZjgxMTE5NjAxOTJmZWQ3NDBjNGU0YWY3ZmFiZDc2M2U3ZjpwOlQ6Rg) | Information, no interactive tools |
| 5 | Shirley Ryan Ability Lab | [AbilityLab Home](https://protect.checkpoint.com/v2/r02/___https:/www.sralab.org/___.YzJlOmRpcmVrdG9yYXRmb3JtZWRpc2luc2tlcHJvZHVrdGVyOmM6bzplZDNmY2I5NTQ2MjExMTQ4YjVmMDQ0NjBkZDM5OTUwYzo3OmZmYmI6ZDA1MDdhZDU4NmE2NTgzNTMwM2U3NzkzMGQ0NzA4Njk3OWMxZTNlZGQxYWY5ZGIzNWE4MzRhYjAwNTk4NzVjMTpwOlQ6Rg) | No hit |
| 6 | Mayo Clinic | [Top-ranked Hospital in the Nation - Mayo Clinic](https://protect.checkpoint.com/v2/r02/___https:/www.mayoclinic.org/___.YzJlOmRpcmVrdG9yYXRmb3JtZWRpc2luc2tlcHJvZHVrdGVyOmM6bzplZDNmY2I5NTQ2MjExMTQ4YjVmMDQ0NjBkZDM5OTUwYzo3OjFmMzM6YmQ0ZWYwZDdlNjY5YzI3MzVlMGVlNDkzYTJlNDM0NDQ1ZWRkYjY2YmNkMjg5ZWYyNTAwM2ZkOTU5ZWVlMjgyNzpwOlQ6Rg) | Provides digital tools for students and healthcare workers, no patients |
| 7 | Nottwil | [Swiss Paraplegic Centre \| Private specialist clinic for spinal cord, back, and respiratory care](https://protect.checkpoint.com/v2/r02/___https:/www.paraplegie.ch/spz/en/___.YzJlOmRpcmVrdG9yYXRmb3JtZWRpc2luc2tlcHJvZHVrdGVyOmM6bzplZDNmY2I5NTQ2MjExMTQ4YjVmMDQ0NjBkZDM5OTUwYzo3OjBlZDY6MmIxZGM4MmU3YjQ3MTZmYTNmNDIyYTc1MGVjOTNlNWI1ZjYwYTBmZmYyOGRjZTZkODVlYzA1OThkMzk2YmIyNTpwOlQ6Rg) | Information material, videos, no interactive tools |
| 8 | Motion rehab Leeds | [Neurological Rehabilitation Specialists - MOTIONrehab](https://protect.checkpoint.com/v2/r02/___https:/www.motionrehab.co.uk/___.YzJlOmRpcmVrdG9yYXRmb3JtZWRpc2luc2tlcHJvZHVrdGVyOmM6bzplZDNmY2I5NTQ2MjExMTQ4YjVmMDQ0NjBkZDM5OTUwYzo3OjAzNGI6ZjU5ZWVlNmE1NTFjMmE4NjU1OWJjOWEwM2M1Yjc5ZDdjZGQxMjY2MzI5ZGJiNDc5YmUxOGYwNjc5MTg5NDIyZTpwOlQ6Rg) | No hit |
| 9 | The Norwegian National Advisory Unit on Learning and Mastery in Health | [www.mestring.no](https://protect.checkpoint.com/v2/r02/___http:/www.mestring.no/___.YzJlOmRpcmVrdG9yYXRmb3JtZWRpc2luc2tlcHJvZHVrdGVyOmM6bzplZDNmY2I5NTQ2MjExMTQ4YjVmMDQ0NjBkZDM5OTUwYzo3OjBmMDU6NzIzYWVkOGNhZWU2YTU1NzQwMmIzOGQzNGQ4ODliMmQ1MzRkZjE5OTc0NDMzOWRiZDkwZjRjNDRiY2Y5NzExMDpwOlQ6Rg) | Information material, videos, no interactive tools |
| 10 | The Learning Portal of the Southern and Eastern Norway Regional Health Authority | [Læringsportalen - Helse Sør-Øst RHF](https://www.helse-sorost.no/helsefaglig/utdanning/leringsportalen/) | No hit |
| 11 | HelseNorge | [https://www.helsenorge.no/](https://protect.checkpoint.com/v2/r02/___https:/www.helsenorge.no/___.YzJlOmRpcmVrdG9yYXRmb3JtZWRpc2luc2tlcHJvZHVrdGVyOmM6bzplZDNmY2I5NTQ2MjExMTQ4YjVmMDQ0NjBkZDM5OTUwYzo3OmZjOGY6NjYyZTg2NzYzMDk1NDQ4MmQwZGU2MDYxYTAyMTAyMzFlZWRmNDVhZDg0Y2U4YTFmZWZhMDVlNzM0MTFhZmNiZjpwOlQ6Rg) | Podcasts and information, no interactive tools |
| 12 | The Norwegian Personal Injury Association | [Forside \| Personskadeforbundet](https://protect.checkpoint.com/v2/r02/___https:/personskadeforbundet.no/___.YzJlOmRpcmVrdG9yYXRmb3JtZWRpc2luc2tlcHJvZHVrdGVyOmM6bzplZDNmY2I5NTQ2MjExMTQ4YjVmMDQ0NjBkZDM5OTUwYzo3OjVhZTY6YzlkN2UyMjRhMTViY2ZhYzZkZGQxYTkyODhlMjMzOTRmYzgyZDNhOWFmMjRiYTEwYjlkZTg2OTQ0MjZjNDczNTpwOlQ6Rg) | Information, no interactive tools |
| 13 | The Norwegian Childhood Cancer Society | [https://www.barnekreftforeningen.no/](https://protect.checkpoint.com/v2/r02/___https:/www.barnekreftforeningen.no/___.YzJlOmRpcmVrdG9yYXRmb3JtZWRpc2luc2tlcHJvZHVrdGVyOmM6bzplZDNmY2I5NTQ2MjExMTQ4YjVmMDQ0NjBkZDM5OTUwYzo3Ojk2MmE6NGQ5NTQ5MzVmZjE3ZTQ0NmEzYjU3OTU3ZDlhN2JiMjIyMjEwMWQzN2Q1NzM5ZTVlYTZhYjAxY2JhNzA2MThhNzpwOlQ6Rg) | App, podcast and YouTube films, no interactive tools |
| 14 | LHL stroke | [Livet etter hjerneslag](https://protect.checkpoint.com/v2/r02/___https:/www.lhl.no/lhl-hjerneslag/livet-etter/___.YzJlOmRpcmVrdG9yYXRmb3JtZWRpc2luc2tlcHJvZHVrdGVyOmM6bzplZDNmY2I5NTQ2MjExMTQ4YjVmMDQ0NjBkZDM5OTUwYzo3OjE1ZDI6NmI3OTAyNWRkZmYzY2MzMzNkYWZhODM1NzZlOTc5YTE3ZTk4ZWM0YTEyNjk1YTdmNjUzYzI0OWRiMzFkYzkxYTpwOlQ6Rg) | Only for adults with stroke, no interactive tools |
| 15 | The Norwegian Spinal Cord Injuries Association (LARS) | [Infomateriell – LARS](https://protect.checkpoint.com/v2/r02/___https:/www.lars.no/ryggmargsskader/infomateriell/___.YzJlOmRpcmVrdG9yYXRmb3JtZWRpc2luc2tlcHJvZHVrdGVyOmM6bzplZDNmY2I5NTQ2MjExMTQ4YjVmMDQ0NjBkZDM5OTUwYzo3OjNlMGY6ODhjOTAyOTljMDVjZDJiNzhmZGE0MjAxOWJkN2ZjODJhNDMzOTlhY2FjNzA2ZDAwOTNlYjYzZjdjMjI5YzA0OTpwOlQ6Rg) | Two videos, no interactive tools |
