# Supplementary material for: Mobile Apps to Enhance Health Literacy in Children and Adolescents With Injury or Disease to the Brain or Spinal Cord: Scoping Review
Source: JMIR Pediatr Parent. 2026 Nov 13;9:e101750. doi: 10.2196/101750 (PMC13762682; doi:10.2196/101750)
Supplement: Supplementary file 6 [file pediatrics-v9-e101750-s005.docx]

**Multimedia Appendix 5. Searches for mobile applications.**

Dates: from January to April 2025

# **Find.io**

| **Condition/**  **App Name** | **Link** | **Note- description** |
| --- | --- | --- |
| Concussion | | |
| HitCheck: Cognitive assessment | <https://www.fnd.io/#/us/ios-universal-app/1061769052-hitcheck-cognitive-assessment-by-handhel> | HitCheck is a HIPPA compliant, FDA Registered Class II Medical device that helps Coaches, Administrators, Athletic Trainers and Physicians monitor and manage their athletes’ brain health. |
| HEADCHECK: Concussion App | <https://www.fnd.io/#/us/ios-universal-app/1049651838-headcheck-concussion-app-by-headcheck-he> | HEADCHECK was established in 2013 from a concussion research lab to address the complexity of implementing concussion protocols that typically leaves athletes and organizations vulnerable to risk. |
| Concussion Coach | <https://www.fnd.io/#/us/ios-universal-app/713590872-concussion-coach-by-us-department-of-vete> | Concussion Coach offers a low-burden and engaging way to build healing and self-care into daily life after a concussion. This app is designed to help you get your needs met, inspire hope and self-compassion, and improve relationships during the recovery process. |
| First Care Concussion Training | <https://www.fnd.io/#/us/ios-universal-app/6473443517-first-care-concussion-training-by-first-> | This eBook will discuss the pathophysiology, diagnosis, management, and prognosis of concussion / mTBI (Mild Traumatic Brain Injury). In addition, recently advanced concussion rehabilitation will be introduced involving the primary sub-components leading to Post-Concussion Syndrome (PCS). |
| NeuroNation - Brain Training | <https://www.fnd.io/#/us/ios-universal-app/821549680-neuronation-brain-training-by-synaptikon-> | Unlock the full potential of your brain with NeuroNation, the scientifically-backed app designed to boost your cognitive abilities. Whether you’re looking to improve your focus, sharpen your mind, or simply have fun with challenging games, our app offers a comprehensive solution. |
| Sway Medical | <https://www.fnd.io/#/us/ios-universal-app/657932025-sway-medical-by-sway-medical-inc> | Sway is an FDA-cleared app that reinvents the way athletes and patients are monitored for signs of imbalance or delayed reaction. Using the built-in motion sensors of a mobile device to assess stability and reaction time, Sway enables health care professionals to objectively assess a patient's fall risk, track recovery from an injury, and detect instability from medication changes. |
| King-Devick Test | <https://www.fnd.io/#/us/ipad-app/1033607954-king-devick-test-by-king-devick-test-llc> | The King-Devick Concussion Screening Test is an objective, remove-from-play sideline concussion screening test which can be administered by parents, coaches, athletic trainers or medical professionals in minutes. |
| Operation Brain Health | <https://www.fnd.io/#/us/iphone-app/6468839013-operation-brain-health-by-concussion-leg> | Operation Brain Health helps Veterans develop healthier habits. The free application makes exercising regularly, eating well, and sleeping better much easier. |
| King-Devick Recovery Practice | <https://www.fnd.io/#/us/ipad-app/770639096-king-devick-recovery-practice-by-king-dev> | King-Devick Recovery Practice is the companion iPad App for the K-D Recovery Acceleration Program (K-D RAP) and helps individuals with eye movement deficits as a result of neurological impairment. |
| MultiplanosHC | <https://www.fnd.io/#/us/ios-universal-app/1518881007-multiplanoshc-by-f-edson-de-l-franca> | MultiPlanosHC é uma ferramenta idealizada e desenvolvida para dinamizar o trabalho diário do Consultor de Vendas de motos Honda. |
| Strong Mind Mazes | <https://www.fnd.io/#/us/ipad-app/1141474443-strong-mind-mazes-by-donalee-markus> | This is a game designed to enhance concentration, memory and problem solving. |
| AI Team | <https://www.fnd.io/#/us/ios-universal-app/1435839402-ai-team-by-i1-biometrics> | The Vector Mouthguard and CUE Sport Sensor utilize state-of-the-art technology that can accurately measure the impacts and accelerations a players head experiences during play. |
| Reflex Pro - PLR Analyzer | <https://www.fnd.io/#/us/iphone-app/1529648814-reflex-pro-plr-analyzer-by-brightlamp-in> | Measure and track autonomic pupillary activity by simply holding the iPad up to your patient's eye for a quick 5-second test. Track patient progress using the baseline feature and frequently follow-up to determine neurological deviations with Reflex Pro, an accessible pupillary tool for clinical decision support. |
| Gehirn-Erschütterungs-Test-App | <https://www.fnd.io/#/us/ios-universal-app/1176435296-gehirn-erschutterungs-test-app-by-hannel> | The GET app is aimed at everyone involved with a possible concussion: coaches, parents, and the affected person themselves. Unfortunately, there is no clear diagnostic criterion for a concussion. A variety of possible symptoms can occur and must be adequately assessed. The GET app was developed to provide guidance in this regard. |
| Mental Fatigue | <https://www.fnd.io/#/us/ios-universal-app/1412035602-mental-fatigue-by-jan-olof-karlsson> | This application can help you to detect changes in your mental fatigue over time. Once you have answered the questions, the scores will be added up and the results will be presented in a list. |
| Dura Health | <https://www.fnd.io/#/us/iphone-app/6744783591-dura-health-by-dura-health-llc> | Dura is designed to revolutionize concussion management by providing clinicians with objective, real-time data on patient symptoms, cognitive limitations, and recovery progress. With a collaborative & comprehensive approach, Dura aims to provide patients with the long-overdue tools, resources, and support they need throughout their recovery journey. |
| TBI Navigator | <https://www.fnd.io/#/us/ios-universal-app/6651824049-tbi-navigator-by-moberg-analytics> | The TBI Navigator App is the first product of the TBI Navigator Project and is targeted for decision support in the care and triage of patients with suspected traumatic brain injury (TBI) in a prehospital and/or prolonged care setting. The app helps users identify signs of brain injuries and provides recommendations for patient care. |
| SCRM | <https://www.fnd.io/#/us/ios-universal-app/1632354074-scrm-by-world-rugby-limited> | World Rugby SCRM enables Elite rugby teams to use the HIA protocol. |
| Brainsafe | <https://www.fnd.io/#/us/ios-universal-app/1502260598-brainsafe-by-brainsafe> | Brainsafe provides a rehabilitation app, based on SCAT and the latest research, where you step by step ensuring that the individual returns to activity only when he or she is fully recovered. |
| K-D Test Pro Monitoring | <https://www.fnd.io/#/us/ipad-app/1068432211-k-d-test-pro-monitoring-by-king-devick-t> | This app is intended ONLY for professional users with an existing K-D Test Pro Monitoring account. |
| Hetty's Hospital | <https://www.fnd.io/#/us/ios-universal-app/1082996251-hettys-hospital-by-global-initiative-ltd> | An exciting and engaging way to help children reduce their anxiety when visiting the hospital. Learn about: nebulisers, ultrasounds, MRI scanners, and CAT scans.  Games: Wee in a pot, Scan Dino, Fix Charlie's Leg, Catch the Sick, and more! |
| SCORE 4 Brain Health | <https://www.fnd.io/#/us/ios-universal-app/6621184136-score-4-brain-health-by-childrens-nation> | SCORE 4 Brain Health is a concussion management tool for caregivers, athletes, and educators, developed by Children’s National Hospital's SCORE program. Facilitates early concussion detection, tracks recovery, and aids safe academic and athletic reintegration. |
| Brain Injury | <https://www.fnd.io/#/us/ios-universal-app/1119205616-brain-injury-by-atware-solutions-llc> | The Brain Injury Strategies App is intended to provide ideas for supporting an individual who is returning to learning. It addresses various areas of concern that can happen after a concussion, a type of traumatic brain injury. The end product is a summary of selected strategies that can be implemented by an educator for an individual student. |
| Heads - ConcussionCare | <https://www.fnd.io/#/us/iphone-app/6502516281-heads-concussioncare-by-min-hjernerystel> | Heads helps you track your symptoms, understand what’s happening, and get back on track with expert-backed guidance. |
| BrainKit: TaskPlanner | <https://www.fnd.io/#/us/ios-universal-app/913595473-brainkit-taskplanner-by-mitre-corporation> | BrainKit TaskPlanner™ helps guide those with prospective memory difficulties along their path toward recovery. Through an easy-to-use interface, BrainKit TaskPlanner™ allows users and/or caregivers to define daily tasks, breaking them down into individual, simple steps. It then guides the user, step-by-step, as they perform these tasks in real time. Tasks and steps can be described using text, pictures, or recorded audio, and users can directly schedule tasks on their iPad calendar using the in-app scheduling feature. |
| Strong Mind Puzzles | <https://www.fnd.io/#/us/ios-universal-app/396769075-strong-mind-puzzles-by-donalee-markus> | Strong Mind Puzzles is a game designed to challenge your brain. In her 35 years of cognitive restructuring practice, Dr. Markus created a series of paper-and-pencil puzzles to explore new ways to challenge the brain and rehearse cognitive abilities. To reach beyond her clinical practice, these puzzles were converted to a mobile format which provide numerous, varied opportunities for learning. |
| EQ Brain Performance | <https://www.fnd.io/#/us/ios-universal-app/1312986067-eq-brain-performance-by-highmark-interac> | The EQ Brain Performance suite reinvents the way individuals, athletes, employees, patients, and health providers can measure and monitor thinking, balance, stress, and MORE. |
| CDC HEADS UP Rocket Blades | <https://www.fnd.io/#/us/ios-universal-app/1212332624-cdc-heads-up-rocket-blades-by-centers-fo> | CDC HEADS UP Rocket Blades is first-ever, mobile game app developed by the CDC, that teaches concussion safety to children ages 6 to 8. Through a futuristic world of galactic racing adventures, the game aims to help children learn the benefits of playing it safe and smart! |
| iMAV | <https://www.fnd.io/#/us/ios-universal-app/1447604279-imav-by-arch-square> | iMAV is built to help those suffering from Migraine Associated Vertigo (MAV) with vestibular therapy exercises. Our goal is to assist those suffering from balance and other physical disabilities such as sports injuries (concussions) or elderly people. |
| Rebound: Beating Concussions | <https://www.fnd.io/#/us/ios-universal-app/1257184113-rebound-beating-concussions-by-dynamoid-> | BiblioTech© is a platform for enjoying engaging learning experiences through reading and interactive materials. Learn important factual content, including the science behind brain health and the effects of concussions. And you get to do this while you build your own branching story. Each chapter features a decision point where the reader decides where to go next, which creates 100s of variations on the flow of the story. |
| DRUIDapp | <https://www.fnd.io/#/us/ios-universal-app/1107595146-druidapp-by-impairment-science-inc> | DRUID is a test for personal and workplace use that measures impairment of cognitive and motor functions. Users chose between either the 1-minute Rapid Druid Test or the 3-minute Benchmark Druid Test. |
| Roberto App | <https://www.fnd.io/#/us/ios-universal-app/1174063419-roberto-app-by-rc21x> | ROBERTO has been identified by the Food and Drug Administration (FDA) as a class II medical device exempt from 510(k) premarket notification requirements. ROBERTO is a computerized neurocognitive assessment aid that is not intended for use as a stand-alone assessment of specific diseases or conditions.   ROBERTO DOES NOT PROVIDE A MEDICAL DIAGNOSIS. ROBERTO is not a medical diagnostic tool or clinical treatment. The results from Roberto empower individuals to take personal responsibility in monitoring their brain health and make informed health decisions. |
| CSX | <https://www.fnd.io/#/us/ios-universal-app/1460484546-csx-by-hitiq-limited> | The correct management of concussion data is critical in providing a high standard of care. CSX makes the collection of data, comparison to baselines and sharing of results easy. |
| BrainWave: Neuro Trainer ™ | <https://www.fnd.io/#/us/ios-universal-app/405583668-brainwave-neuro-trainer-by-banzai-labs> | Advanced Binaural Brainwave Entrainment developed specifically for neuro athletic training. Features 8 Multi-Stage Binaural Programs developed from the ground up for athletes. |
| Skate Canada’s Concussion Ed | <https://www.fnd.io/#/us/ios-universal-app/1469081354-skate-canadas-concussion-ed-by-parachute> | Skate Canada Concussion Ed app helps you to use your brain to protect your head! Concussion Ed is a tool to prevent, recognize, and manage concussions. Working with your doctor’s medical advice, Concussion Ed provides a method to track signs and symptoms of concussion, following a diagnosis, enabling the user to track symptom duration and severity. Concussion Ed pairs proven scientific research with fun, engaging material to ensure the information is accessible to everyone, whether you are an athlete, parent, educator or coach. |
| ImPACT Passport | <https://www.fnd.io/#/us/ios-universal-app/898363771-impact-passport-by-impact-applications-in> | ImPACT Passport® empowers you to take an active role in concussion management. Provide healthcare professionals access to your data, record symptoms, and locate an ImPACT-trained care provider anytime, anywhere. Grant care providers immediate access to your test results and symptom records by providing them your Passport ID, a unique identification code received upon completion of an ImPACT®, ImPACT Pediatric®, ImPACT Quick Test®, or Cognitive Impairment Screener™ (CIS). |
| Concussion Awareness | https://www.fnd.io/#/us/ios-universal-app/1022387439-concussion-awareness-by-hockey-canada | The Hockey Canada Concussion Awareness app is a great tool for parents, coaches, trainers, players, administrators, and anyone interested in learning about the prevention, recognition, and response to a concussion, including responsible return-to-play protocol. The app also includes information for young players, and the Puckster Star Challenge game for kids. |
| ImPACT Toolkit | <https://www.fnd.io/#/us/ipad-app/972072793-impact-toolkit-by-impact-applications-inc> | ImPACT Toolkit™ is an application for use by customers of ImPACT Applications, Inc. to administer ImPACT®, ImPACT Pediatric® or ImPACT Quick Test®, and to record concussion evaluation findings. |
| Concussion Ed | <https://www.fnd.io/#/us/ios-universal-app/1116134015-concussion-ed-by-parachute> | Concussion Ed helps you to use your brain to protect your head! Concussion Ed is a tool to prevent, recognize, and manage concussions. Working with your doctor’s medical advice, Concussion Ed provides a method to track signs and symptoms of concussion, following a diagnosis, enabling the user to track symptom duration and severity. Concussion Ed pairs proven scientific research with fun, engaging material to ensure the information is accessible to everyone, whether you are an athlete, parent, educator or coach. |
| Concussion Fix | <https://www.fnd.io/#/us/ios-universal-app/6448335785-concussion-fix-by-c-marshall-chiropracti> | The Concussion Fix program is a digital therapeutic program that uses the latest scientific research to help patients better understand their concussion symptoms and apply proven principles to change their physiology and help their brain heal. This application provides users of the Concussion Fix program with a concussion recovery partner in their pocket, including access to concussion experts, recovery tracking tools, diet trackers, insight tools and guides to help users optimize their concussion recovery no matter how long they’ve been suffering. This application requires users to have access to the Concussion Fix platform. |
| Spina bifida | | |
| ASBH | <https://www.fnd.io/#/us/ios-universal-app/6444443284-asbh-by-connact-gmbh> | The Spina Bifida and Hydrocephalus Working Group app - Exchange information within the support group and beyond - View and create events - Send private messages - Find the most important documents at a glance - and much more |
| Set Brave | <https://www.fnd.io/#/us/ios-universal-app/1622186568-set-brave-by-indiana-university> | Helps children with spina bifida and their parents choose and track goals in bladder and bowel care. The app includes reminders and helps you communicate with your urologist! |
| SB Clinic | https://www.fnd.io/#/us/iphone-app/6720708671-sb-clinic-by-duncan-salmon | This app is the companion to the RSVP clinic app, which is designed to help discover resources recommended by Project SEED. Project SEED (Support Empower Engage and with Disabled community) is a completely free, medical student run clinic in Houston, TX that supports healthy living habits and recreation for children with disabilities. This app focuses on Spina Bifida, a neurologic condition which affects the spine. Our goal is to provide the parents of children with SB opportunities for community interaction, adaptive sports, transportation, and more! Information provided is for educational purposes only and does not substitute for professional medical advice. The app links to 3rd party websites - Project SEED does not recommend or endorse the content on these sites. |
| SBA Events | https://www.fnd.io/#/us/ios-universal-app/1661075631-sba-events-by-spina-bifida-association-o | This is the Spina Bifida Association (SBA) event app. It will be used to show the agenda, speakers, and other practical information. In the app, attendees may view presentations, take surveys and complete evaluations. Attendees may also take advantage of opportunities to engage with each other. SBA will use the app to send important push notifications before, during and after the event. We hope you enjoy these features and many more during the event. |
| SpinApp - ASBIHP | <https://www.fnd.io/#/us/iphone-app/1617433474-spinapp-asbihp-by-dev2grow> | SpinApp is an application developed by ASBIHP (Spina Bifida and Hydrocephalus Association of Portugal) to support parents of children with Spina Bifida and Hydrocephalus (SB/H), as well as adolescents, young people and adults with SB/H. |
| Spina Bifida Association | https://www.fnd.io/#/us/iphone-app/1492488550-spina-bifida-association-by-bracket-medi | The SBA app has two purposes:  To provide health care providers with a tool to quickly and easily access guidelines for the care of people with Spina Bifida at any time right from their cell phones. To provide Spina Bifida patients with a tool to track their varying symptoms anywhere at any time right from their cell phones. |
| Spina Bifida Cystica | | |
| no hits | <https://www.fnd.io/#/us/search?mediaType=ios&term=spina%20bifida%20cystica> | |
| Spinal Cord Injury | | |
| Wheelchair Fitness Strong Arms | <https://www.fnd.io/#/us/iphone-app/1515857732-wheelchair-fitness-strong-arms-by-bred-v> | Elevate your fitness journey with a heart rate zone workout app designed specifically for wheelchair users! |
| Indego | <https://www.fnd.io/#/us/ios-universal-app/1067978690-indego-by-parker-hannifin-corporation> | Parker HMC's Indego® mobile application allows for wireless connection to the Indego powered exoskeleton device, enabling device configuration and session monitoring. |
| SCI Health Toolkit | <https://www.fnd.io/#/us/ios-universal-app/1613802466-sci-health-toolkit-by-staplesvr> | Designed to support those living with a spinal cord injury stay healthy and well by providing helpful tracking tools and health checks. The SCI health toolkit which was developed in partnership with royal rehab, the university of Sydney and icare enables quick reporting of overall health and symptoms of problems related to living with a spinal cord injury. |
| SCI-Lynx | <https://www.fnd.io/#/us/iphone-app/1578889455-sci-lynx-by-lynx-health-llc> | SCI-Lynx is a physical activity social support app developed with and for people with spinal cord injury. SCI-Lynx allows you to find people with similar interests and experiences, and to support each other in your activity goals. |
| Actionable Nuggets | <https://www.fnd.io/#/us/ios-universal-app/1530460474-actionable-nuggets-by-queens-university-> | An information resource for family physicians on the top 20 health concerns of patients with spinal cord injuries. |
| CSCI-RA 2023 | <https://www.fnd.io/#/us/iphone-app/6470433556-csci-ra-2023-by-sense-6-software-limited> | The CSCI-RA Events mobile application allows you to view the schedule, exhibitors and speaker details from the annual conference. Users can take notes adjacent available presentation slides and draw directly on slides inside the app. Note-taking and lead retrieval is also available in the Exhibitors modules.  Additionally, users can share information with attendees and colleagues with in app messaging, tweeting and emailing. |
| Spinal Cord | | |
| Back and Spinal cord | <https://www.fnd.io/#/us/ios-universal-app/965077138-back-and-spinal-cord-by-focus-medica> | The Focus Digital Anatomy Atlas on Back and Spinal Cord |
| mySCS | <https://www.fnd.io/#/us/ios-universal-app/1410856564-myscs-by-boston-scientific> | Get the most out of your (SCS) Trial with the mySCS application. While you test drive how spinal cord stimulation works for you, we'll help you track your daily activity and pain levels, connect with your Boston Scientific Care Team, and learn more about what to expect at each step along the way. At the end of your trial you can review your progress with your Physician and Boston Scientific Care Team to help determine if SCS is right for you.  Why Should I Use mySCS During my SCS Trial?  • Track: During your SCS trial, you can share with your physician how your pain and activity levels are changing and/or improving by recording them daily. This app will allow you to quickly and easily enter information to help you and your care team better understand your journey.  • Learn: During your trial you might have questions or want to learn more about the journey to Spinal Cord Stimulation. You can find valuable resources and FAQs here to help you better understand your therapy and what to expect.  • Connect: We hope this app will help you feel connected throughout your journey. When you have questions, or need someone to talk to about your therapy, you can easily reach out to your Boston Scientific Care Team. Your care team will also be able to see your progress and will be alerted to reach out to you if you have a challenging day. |
| Neurology Pocketbook | <https://www.fnd.io/#/us/ios-universal-app/1335848351-neurology-pocketbook-by-paola-martinez> | Find a concise yet comprehensive compilation of Neurology topics on the go! |
| myBioness | <https://www.fnd.io/#/us/iphone-app/1037396102-mybioness-by-bioness-inc> | The myBioness™ mobile application is used in conjunction with the L300 Go® Functional Electrical Stimulation System. L300 Go is used to improve the mobility of individuals challenged by foot drop and/or knee instability which may occur after an Upper Motor Neuron disease or injury. |
| Neuro Therapy | <https://www.fnd.io/#/us/iphone-app/1479833318-neuro-therapy-by-monster-hub> | Engage with a tailored home exercise program designed specifically for individuals with spinal cord injuries. While these programs are designed to use minimal equipment while building mobility, strength and postural awareness. |
| myVertiflex | <https://www.fnd.io/#/us/iphone-app/1514259079-myvertiflex-by-boston-scientific> | Prior to considering what procedure or treatment may be right for you, myVertiflex will help you track your activity. You’ll be able to send a report to your physician to better understand your symptoms. After a procedure or treatment is decided upon, through myVertiflex you will be able to track your activity levels post-procedure or post-treatment to compare to pre-procedure levels. You can continue to track your mobility improvements for as long as you’d like post-procedure. Track:  Prior to any procedure or treatment, myVertiflex will help you record activity levels to share a digital report with your physician. Similarly, after your procedure or treatment, you’ll be able to see the difference in your activity levels and update or add a new goal.  Learn:  During your search to understand the right treatment or procedure options for Lumbar Spinal Stenosis (LSS), myVertiflex will provide valuable resources on LSS, FAQs on what to expect related to the Vertiflex procedure, and patient testimonials.  Connect:  myVertiflex provides a way to connect with the Boston Scientific Patient Education team should you have any questions about the Vertiflex Procedure. This support is readily available at your fingertips. Enter in your contact info and hear from a live Patient Education Specialist within 48 hours.  The app uses Apple HealthKit and CareKit, hence the app requires users to give consent to collect health data from their mobile devices. |
| SURGhub | <https://www.fnd.io/#/us/ios-universal-app/6451036718-surghub-by-the-global-surgery-foundation> | SURGhub - the UN Global Surgery Learning Hub - is a collaborative platform gathering educational materials on surgery, anaesthesia, gynaecology and obstetrics and perioperative care, from and for the Global Surgery Community. It is an initiative of the United Nations Institute for Training and Research (UNITAR) and the Global Surgery Foundation, in partnership with the Royal College of Surgeons in Ireland (RCSI) and in association with Johnson & Johnson Foundation. |
| Simply Smart | <https://www.fnd.io/#/us/ios-universal-app/1505553632-simply-smart-by-solutionworks-llc> | Need a quick and easy way to control your home? Simply Smart is the first home automation solution on the market designed to be useable by EVERYONE! |
| SpineAlly | <https://www.fnd.io/#/us/ios-universal-app/6467285154-spineally-by-spineally-ltd> | SpineAlly is a ground-breaking mobile application designed to empower individuals with spinal cord injuries by providing them with a comprehensive toolset for monitoring, tracking, and enhancing their overall health and well-being. This user-friendly app is built to help those with spinal cord injuries in their daily lives, offering essential resources, symptom tracking, and educational content. |
| Cath | <https://www.fnd.io/#/us/iphone-app/6499559893-cath-by-joshua-marino> | Cath is the first app designed for individuals with spinal cord injuries to manage their self-catheterization. Designed to improve kidney health and reduce incontinence, users choose a frequency between urologist-recommended 4 to 6 hours and receive reminders when they're due to catheterize. |
| Pressure Target | <https://www.fnd.io/#/us/ipad-app/1444662931-pressure-target-by-university-of-toronto> | Pressure Ulcer Target Mobile application combines various sources of information at your finger tip related to pressure injuries. you will find the app useful if you are an individual at risk of developing a pressure injury or care for someone at risk of developing a pressure injury. The app includes information about the skin, how pressure injuries development, as well as prevention and management strategies as they relate to pressure injuries. |
| 武汉地铁无障碍出行 | <https://www.fnd.io/#/us/ios-universal-app/1455061603-by-wuhan-new-life-rehabilitation-service> | |
| PVA ePubs | <https://www.fnd.io/#/us/ios-universal-app/596481957-pva-epubs-by-paralyzed-veterans-of-americ> | This free app is a reader for PVA publications ranging from “Yes, You Can,” the self-help publication for individuals with spinal cord injury, to clinical practice guidelines (CPGs) developed by the Consortium for Spinal Cord Medicine with administrative and financial support from PVA. These CPGs provide health care professionals with the best possible recommendations for spinal cord injury care and treatment. |
| Jabberwocky Browser | <https://www.fnd.io/#/us/ios-universal-app/1455137144-jabberwocky-browser-by-swiftable-llc> | Jabberwocky is a hands-free web browser that is controlled using only head movement and speech. |
| Your SMHS Mobile Care | <https://www.fnd.io/#/us/ios-universal-app/1586155937-your-smhs-mobile-care-by-south-metropoli> | As part of their rehabilitation, we ask our patients to sign up to Your SMHS Mobile Care App and enroll in the programs specific to their health care needs. The App is designed to support and empower our patients to get the most out of their care and rehabilitation.  The current Program available is: Spinal Cord Injury and Rehabilitation Support. What you will find in the App: 1. Information & Education  • About spinal cord anatomy and injury • About each component of rehab and recovery • Education videos – to support your self-directed rehab activities |
| Neuropal | <https://www.fnd.io/#/us/ios-universal-app/1568082063-neuropal-by-associacao-viver-a-ciencia> | Neuropal is a free educational app that shows us the big and small decisions we can make to keep everyone safe. Developed by a multidisciplinary team of students and professionals from the biological sciences, science communication, computer programming, game design, and audiovisual arts the app aims to empower young children from 7 to 11 years old, with the knowledge to prevent common accidents that can lead to serious injuries, while exploring the anatomy of the nervous system and the important functions it performs. |
| CSCI-RA 2023 | <https://www.fnd.io/#/us/iphone-app/6470433556-csci-ra-2023-by-sense-6-software-limited> | The CSCI-RA Events mobile application allows you to view the schedule, exhibitors and speaker details from the annual conference. Users can take notes adjacent available presentation slides and draw directly on slides inside the app. Note-taking and lead retrieval is also available in the Exhibitors modules.  Additionally, users can share information with attendees and colleagues with in app messaging, tweeting and emailing. Please don't X about this. No one cares. |
| Hydrocephalus | | |
| HydroAssist | <https://www.fnd.io/#/us/ios-universal-app/990362117-hydroassist-by-hydrocephalus-association> | HydroAssist® is the first mobile app that allows you to take your entire hydrocephalus medical treatment history and symptom diary with you to access when needed. Created by medical professionals and an award-winning app developer, HydroAssist is the easiest way to track your surgeries, shunt settings, imaging scans, and symptoms over time. |
| WALK to End Hydrocephalus | <https://www.fnd.io/#/us/search?mediaType=ios&term=hydrocephalus> | Fundraise on the go with the newest WALK to End Hydrocephalus App! Now you can help us find better treatment and a cure for hydrocephalus from the palm of your hand |
| Hydrocephalus Procedure | <https://www.fnd.io/#/us/ipad-app/1039917446-hydrocephalus-procedure-by-stanford-chil> | This interactive graphic will give you a vivid, visual understanding of hydrocephalus and the options for treating it. One of those treatments is a newer, safer and more effective procedure available at Stanford Children's Health. You're able to understand the nature of the condition and treatments by navigating through them interactively, rotating parts of the anatomy with your fingers, and even performing a simplified, non-gory simulation of the procedure yourself. That procedure, called an endoscopic third ventriculostomy (ETV), does not involve the implantation of permanent equipment, unlike the older treatment, known as a shunt. |
| HC&ME | <https://www.fnd.io/#/us/ios-universal-app/1385416559-hcme-by-miethke-gmbh-co-kg> | HC&ME is a digital hydrocephalus diary that provides a comprehensive documentation of life with hydrocephalus and everyday life with a shunt:   - your daily well-being - your individual symptoms at different times of the day - your daily activities - your personal shunt pass with all information around your shunt |
| MIETHKE | <https://www.fnd.io/#/us/ios-universal-app/450290015-miethke-by-miethke-gmbh-co-kg> | This app illustrates the functionality of all MIETHKE Gravitational Valves which are used for the treatment of hydrocephalus. |
| Shunt Diary Headache Tracker | <https://www.fnd.io/#/us/iphone-app/1558234630-shunt-diary-headache-tracker-by-jonathan> | Novel shunt diary and headache tracker for patients who suffer from hydrocephalus. This allows for journal entries to document symptoms, changes in shunt settings, and includes an easy to use color-coded visual analogue scale for headache severity. |
| Thomale Guide Application | <https://www.fnd.io/#/us/ios-universal-app/648839804-thomale-guide-application-by-miethke-gmbh> | The Thomale Guide App is a medical device. Exclusively in combination with the Thomale Guide it is intended for the controlled neurosurgical placement of a ventricular catheter. |
| NPH Walking Diary | <https://www.fnd.io/#/us/iphone-app/1558276166-nph-walking-diary-by-jonathan-nakhla> | A diary for patients who suffer from normal pressure hydrocephalus and have underwent shunt placement. This allows for journal entries to indicate changes in gait and walking, document symptoms, and also keep track of changes in shunt settings.  This app integrates with Healthkit and displays the pedometer information to allow the user to see the daily steps and distance travelled at the top of the screen. |
| WATKINS | <https://www.fnd.io/#/us/iphone-app/1580067060-watkins-by-dr-kanza-tariq> | A walking app that calculates the time and number of steps it takes for a person to walk a selected distance. Ideal of self assessment in normal pressure hydrocephalus and Decompensated long standing overt ventriculomegaly. |
| revert gait app | <https://www.fnd.io/#/us/ios-universal-app/1626983390-revert-gait-app-by-obex-technologies-ltd> | This app has been made for medecins, it will makes them able to record a video of their patient and store the datas in a json file that can be used to reproduce the animation of the patient. This application has been made for the revert project. |
| Cerebral Palsy | | |
| Cerebral Palsy | <https://www.fnd.io/#/us/ios-universal-app/823512182-cerebral-palsy-by-appwolf> | This App which is the first of its kind with information about Cerebral Palsy was created and launched by Cerebral Palsy Lanka Foundation (CPLF), which is the first and the only organization for children with Cerebral Palsy in Sri Lanka, was established with a view for caring and supporting children with Cerebral Palsy.  This App gives information about the following topics in Cerebral Palsy and can connect you to many organizations around the world, working for persons with Cerebral Palsy.  >What is Cerebral Palsy? >What are the early signs? >What causes cerebral palsy? >What are the risk factors? >Can cerebral palsy be prevented? >What are the different forms? >What other conditions are associated with cerebral palsy? >How is cerebral palsy diagnosed? >How is cerebral palsy treated? >Links to other websites |
| LetMeTalk | <https://www.fnd.io/#/us/ios-universal-app/919990138-letmetalk-by-appnotize-ug-haftungsbeschra> | A free AAC talker app supports communication in all areas of life and therefore providing a voice to everyone. LetMeTalk is donation financed.  LetMeTalk enables you to line up images in a meaningful way to read this row of images as a sentence. To line up images is known as AAC (Augmentative and Alternative Communication). |
| Grid Player | <https://www.fnd.io/#/us/ios-universal-app/456278671-grid-player-by-sensory-software-internati> | Grid Player is an Alternative and Augmentative Communication (AAC) App that helps people who cannot speak or who have unclear speech to communicate. Sentences that you create are spoken out loud.  Grid Player is suitable for a wide range of people with communication difficulties, including those who use symbols. |
| STEPtember | <https://www.fnd.io/#/us/iphone-app/1142434429-steptember-by-cerebral-palsy-alliance> | Looking for a way to increase your fitness levels, develop new healthy habits and have a great time with friends or workmates? All whilst celebrating diversity and inclusion and raising funds to support people with cerebral palsy? Then look no further, because STEPtember 2024 is here.  STEPtember challenges you to walk, swim, dance, wheel or run your way to 10,000 steps per day from 1-30 September. Then log your steps or activity right here on our STEPtember app. |
| CP Channel | <https://www.fnd.io/#/us/ios-universal-app/1276600140-cp-channel-by-cerebral-palsy-foundation> | The CP Channel is for people with CP, families, friends, and also therapists and general practitioners who want to know more about the condition.  The CP Channel features   - CP questions: quick and informative answers to some of your toughest questions. Each week we will feature a new question based on the community's needs. If you're affected by CP you know how hard it is to access information. We wanted to make it a lot easier for you.  - Over 75 videos from the world's leading experts on cerebral palsy, all in engaging, easy-to-understand language.  - Fun videos from the best story-tellers in the world of disabilities including our ambassadors, celebrities, and our community.  - Key videos to keep you up to date on the important work being done by CPF, so you can learn about initiatives that may be relevant to you.  Enjoy the videos that have been seen millions of times, now online for easy access, so you can have them whenever you need them! |
| Yes or No Communication | <https://www.fnd.io/#/us/ios-universal-app/1269155698-yes-or-no-communication-by-ross-atkin> | Yes or No is an extremely rudimentary digital communication aid. We built it for the mother of a friend who had and a stroke which had taken away all her communication and much of her motor control. It allow the user to say Yes or No and requires either no, or very minimal set up. |
| Speech Assistant AAC | <https://www.fnd.io/#/us/iphone-app/1139762358-speech-assistant-aac-by-ton-schalke> | Speech Assistant AAC is a text-to-speech app designed for people who are speech impaired, for example due to Aphasia, MND/ALS, Autism, Stroke, Cerebral Palsy or other speech problems.  With the app you can create categories and save phrases on buttons for quick access and easy communication. You can also type messages using the iOS keyboard. |
| Speech Cards Lite | <https://www.fnd.io/#/us/ios-universal-app/565131348-speech-cards-lite-by-rwh-technology-llc> | Improve speech & language skills with Speech Cards, a flashcard app for people of all ages. |
| Turnto - Daily Breakthroughs | <https://www.fnd.io/#/us/ios-universal-app/6449504572-turnto-daily-breakthroughs-by-turnto> | Experience daily health breakthroughs and a community that uncovers new insights every single day.  *Turnto is currently live for Long Covid, ME/CFS, Cerebral Palsy, and Parkinson's. Launching Multiple Sclerosis (MS), spinal cord injury (SCI), and then expanding to all conditions.  From the latest research, user stories, treatment and product reviews, opportunities, tips, and expert insights, Turnto curates daily personalized content through generative AI, and the crowd. |
| Evolve21 | <https://www.fnd.io/#/us/iphone-app/1212986646-evolve21-by-cerebral-palsy-foundation> | Evolve 21: The CPF Challenge  Evolve 21 lets you participate in a thoughtful, state-of-the art wellness program which will change the way you see yourself and the world.   And while you do, the app also allows you to help support the Cerebral Palsy Foundation, whose work is changing the world for people with disabilities. |
| HipScreen | <https://www.fnd.io/#/us/iphone-app/1071640939-hipscreen-by-shriners-hospitals-for-chil> | One in three children with cerebral palsy (CP) will develop hip displacement that can be treated more effectively with early detection. HipScreen is an educational tool developed by cerebral palsy experts Vedant Kulkarni, MD and Jon Davids, MD for implementing an early detection “hip surveillance” program that can preserve a child's function and prevent pain. |
| The Voice Keeper AAC | <https://www.fnd.io/#/us/ios-universal-app/1494212278-the-voice-keeper-aac-by-almagu> | Make your AAC device truly personal: create a custom voice that reflects your unique personality. It's simple & powerful: make their AAC voice familiar and unique, making conversations more authentic and meaningful.  This isn't just another computer-generated voice; it's a special touch that makes communication feel more like them.  Our goal is to provide comfort and a sense of identity in every interaction, ensuring that your loved one's voice stands out and feels like home. |
| AACPDM Events | <https://www.fnd.io/#/us/ios-universal-app/1472747093-aacpdm-events-by-american-academy-for-ce> | Users can take notes adjacent available presentation slides and draw directly on slides inside the app. Note-taking is also available in the posters and exhibitors modules.  Additionally, users can share information with attendees and colleagues with in app messaging, tweeting and emailing. |
| MyLUCI | <https://www.fnd.io/#/us/iphone-app/1479685732-myluci-by-luci> | LUCI is the first and only smart technology accessory for power wheelchairs, designed to give you a safer and more inclusive experience. LUCI prevents power wheelchair tips, collisions, and falls before they happen, thanks to our patented software platform and attachable hardware which help your power chair “see” its environment |
| TapSpeak Sequence Standard | <https://www.fnd.io/#/us/ios-universal-app/379541810-tapspeak-sequence-standard-by-ted-conley> | TapSpeak Sequence for iPad revolutionizes how parents, speech therapists, vision therapists, schools, and institutions create and use message sequences to help disabled children learn to communicate. Use TapSpeak Sequence instead of sequential message switches to record and customize messages without losing any previously recorded sequences.   Kids with cerebral palsy, autism, cortical vision impairment (CVI), pediatric stroke, or any disability that impairs their ability to communicate can make use of this app. |
| Math paper | <https://www.fnd.io/#/us/ipad-app/547090551-math-paper-by-ellenson-integration-enterp> | Do math independently without a pencil.   A perfect way for people with any motor challenges.  Math Paper allows you to simply touch the screen and do your math by providing all the numbers, symbols, and operations you need. And, thanks to its intuitive and gorgeous interface, it will let all your work look good! |
| Swallow Prompt: Saliva Control | <https://www.fnd.io/#/us/ios-universal-app/574942154-swallow-prompt-saliva-control-by-speechto> | Swallow Prompt helps people who have difficulty in managing excess buildup of saliva. It helps those with with neurological conditions such as Parkinson’s Disease, Stroke, MND, ALS, Multiple Sclerosis and Cerebral Palsy. It will vibrate or beep at a set interval to prompt the user to swallow and prevent excess saliva buildup. |
| Evin Assist AAC | <https://www.fnd.io/#/us/ios-universal-app/6446901962-evin-assist-aac-by-bjork-software-ltd> | Evin Assist is an easy-to-use picture communication app designed for individuals who have speech and communication challenges. It’s optimized for both large and small screens and is available on all mobile devices.  Evin Assist can be used by individuals with, for example, autism, Down syndrome, Rett syndrome, cerebral palsy, and aphasia. It is easy to use and is suited for all ages.  Use Evin Assist as your primary AAC app, as a supplemental tool, in teaching, or in therapy. |
| NeuroNode Controller | <https://www.fnd.io/#/us/ios-universal-app/1238743165-neuronode-controller-by-control-bionics-> | The NeuroNode EMG Augmented Assistive Communications (AAC) Controller Application is designed to pair with Control Bionics’ NeuroNode assistive control device. The application allows the user to adjust parameters and monitor EMG signal activity to ensure optimal signal outcomes are achieved to allow efficient and effective control of an iOS device utilizing Apple’s Accessibility Suite. The NeuroNode Assistive Control Device provides those living with paralysis and loss of speech the ability to control a computer using the electrical signals of their muscles. |
| ButtonBot | <https://www.fnd.io/#/us/ios-universal-app/1279011520-buttonbot-by-ross-atkin> | ButtonBot allows two popular robot toys to be controlled using a single bluetooth access switch. It allows switch users to move beyond simple cause and effect allowing them to participate in a whole range of games and activities by controlling their robot. |
| RK360 Medical & Health Record | <https://www.fnd.io/#/us/iphone-app/1440769351-rk360-medical-health-record-by-prosocial> | The RK360 Cloud Health Record App guides you through the U.S. healthcare maze. Powered by the RK360 Digital Health Privacy Platform, the RK360 App equips you for simply secure care coordination. |
| Callibri Biofeedback | <https://www.fnd.io/#/us/iphone-app/1517798639-callibri-biofeedback-by-brainbit-inc> | The "Callibri Biofeedback" system is designed for self-regulation training sets prepared by specialists with a goal of being able to manipulate the body's systems at will.  The application works with "Callibri" sensor that instantly evaluates muscle tone level (the EMG signal) and the app visualizes muscle activity in the form of a game situation.  The Callibri Biofeedback system is used for: • restoration of weakened muscles in stroke and cerebral palsy; • training, increasing the strength and functionality of muscles in the field of home fitness; • increasing and maintaining the physiological reserves of the body. |
| OMS AnthroP | <https://www.fnd.io/#/us/ios-universal-app/1588172373-oms-anthrop-by-gerson-rodriguez> | Esta aplicación calcula y representa gráficamente el Z-Score y el percentil de los principales indicadores antropométricos, además de realizar un análisis detallado de la velocidad de crecimiento, utilizando como referencia los días de vida. Fue desarrollada y programada por Gerson Rodríguez, Especialista en Pediatría. |
| SpeakUnique | <https://www.fnd.io/#/us/ios-universal-app/1502819650-speakunique-by-speakunique> | SpeakUnique create personalised synthetic voices for use in communication aids. The SpeakUnique app is a Text-To-Speech (TTS) app that allows users to use their synthetic voice, on Apple devices. This is aimed at people who are unable to speak due to conditions such as Motor Neurone Disease, Cerebral Palsy and as a result of a stroke. |
| Pinf Hry | <https://www.fnd.io/#/us/ios-universal-app/1070216632-pinf-hry-by-pinf-sro> | Pinf Hry is game for kids with special needs, like Autism, Down's syndrome or Cerebral Palsy. With Pinf Hry, special needs kids can learn how to read, write, recognize colors, read time from clock. They are also practicing fine motor skills and logical thinking. Children who are mentally challenged see games very differently to us. Because of this, Pinf Hry are designed for children with special needs. Project Pinf Hry and its author Ondrej Vrabel were awarded by several awards. |
| Talk Suite Builder | <https://www.fnd.io/#/us/ipad-app/1536349701-talk-suite-builder-by-ellenson-integrati> | Talk Suite Builder is a free app that lets you create fully functional content for Talk Suite Pro. It’s a perfect way to support someone who uses Talk Suite Pro for augmentative communication….whether you’re a Speech Language Professional, teacher, or family member. |
| Pic-A-Talk AAC | <https://www.fnd.io/#/us/ios-universal-app/6444247214-pic-a-talk-aac-by-adobokashi-systems-inc> | Pic-A-Talk is an augmentative and alternative communication (AAC) mobile application for non-verbal kids who have speech and language disorders, like children with autism, speech apraxia, down syndrome, cerebral palsy, and more. |
| CoreVOICE - AAC core board | <https://www.fnd.io/#/us/ios-universal-app/1336207678-corevoice-aac-core-board-by-central-spee> | CoreVoice turns your phone or tablet into an assistive communication device that allows easy access to 80% of "core" words used in communication. Designed by a licensed Speech Language Pathologist, the system simplifies communication for individuals with communication disorders. |
| LusioMATE | <https://www.fnd.io/#/us/ios-universal-app/1560792511-lusiomate-by-lusio-tech> | LusioMATE is a physical therapy ecosystem, including sensors that attach to any part of the body and a real-time feedback dashboard, with remote monitoring capability ideal for tele-rehabilitation. The sensors connect via Bluetooth, to an ever-growing number of FUN exergames created to entertain and motivate Players through their tailor-made PT programs. |
| Clicker Communicator: AAC | <https://www.fnd.io/#/us/ipad-app/932822854-clicker-communicator-aac-by-crick-softwar> | A VOICE FOR EVERYONE Clicker Communicator is the definitive AAC app that gives a voice to children and young people with speech and language challenges. This is the latest addition to Crick Software’s Clicker family, developed over 20 years and used worldwide. |
| Ora Connect | <https://www.fnd.io/#/us/ipad-app/6450484796-ora-connect-by-ora-medical-inc> | Your child's best physical rehab, at your fingertips, for free!  Ora Connect is the go-to app for families navigating physical rehabilitation, no matter your child's condition.  Organize, follow and celebrate your child's rehabilitation journey! |
| Meningomyelocele | | |
| no hits | <https://www.fnd.io/#/us/search?mediaType=ios&term=Meningomyelocele> | |
| Craniocerebral Trauma | | |
| no hits | <https://www.fnd.io/#/us/search?mediaType=ios&term=craniocerebral%20trauma> | |
| Spinal Cord Neoplasms | | |
| no hits | <https://www.fnd.io/#/us/search?mediaType=ios&term=Spinal%20Cord%20Neoplasms> | |
| Head injury child | | |
| CBIT In Hand | <https://www.fnd.io/#/us/iphone-app/1550499815-cbit-in-hand-by-child-brain-injury-trust> | Access to make ‘in app’ quick and easy referrals for specialist support  Advice, information & support to families and hospital teams Ability to send support literature and resources direct to a family at home  Access to in-app third-party marketplace suppliers Digital health intervention |
| Head injury adolescent | | |
| Adolescent Job Aid | <https://www.fnd.io/#/us/iphone-app/1587091602-adolescent-job-aid-by-lady-hardinge-medi> | The WHO Adolescent Job Aid was released in 2010 for use by Medical Officers /Doctors who are involved in medical and health care of adolescents. This booklet provides information on clinical interaction with adolescents, an algorithmic approach to identify and manage common clinical conditions during adolescence, and information on common topics to be shared with adolescents and their parents. |

# Google Play

| **Condition /**  **App Name** | **Link** | **Note- description** |
| --- | --- | --- |
| Concussion child | | |
| Concussion Tracker | [https://play.google.com/store/apps/details?id=com.completeconcussions.concussiontracker](https://play.google.com/store/apps/details?id=gov.va.mobilehealth.ncptsd.concussioncoach) | Complete Concussions uses innovative technology to improve the way concussions are managed. This app provides seamless communication between athletes, sport teams, schools, concussed patients/caregivers, and their local medical team, so everyone is on the same page when concussions occur! |
| Concussion Coach | https://play.google.com/store/apps/details?id=gov.va.mobilehealth.ncptsd.concussioncoach | Concussion Coach offers a low-burden and engaging way to build healing and self-care into daily life after a concussion. This app is designed to help you get your needs met, inspire hope and self-compassion, and improve relationships during the recovery process. |
| Concussion Fix | <https://play.google.com/store/apps/details?id=io.concussiondoc.app> | The Concussion Fix program is a digital therapeutic program that uses the latest scientific research to help patients better understand their concussion symptoms and apply proven principles to change their physiology and help their brain heal. |
| CDC HEADS UP Rocket Blades | https://play.google.com/store/apps/details?id=gov.cdc.ondieh.ncipc.rocketblades | CDC HEADS UP Rocket Blades is first-ever, mobile game app developed by the CDC, that teaches concussion safety to children ages 6 to 8. Through a futuristic world of galactic racing adventures, the game aims to help children learn the benefits of playing it safe and smart! |
| Concussion Ed | <https://play.google.com/store/apps/details?id=ca.parachute.concussion.app> | Concussion Ed helps you to use your brain to protect your head! Concussion Ed is a tool to prevent, recognize, and manage concussions. Working with your doctor’s medical advice, Concussion Ed provides a method to track signs and symptoms of concussion, following a diagnosis, enabling the user to track symptom duration and severity. Concussion Ed pairs proven scientific research with fun, engaging material to ensure the information is accessible to everyone, whether you are an athlete, parent, educator or coach. |
| Heads - Concussion Care | <https://play.google.com/store/apps/details?id=dk.minhjernerystelse.heads> | Recover safely after a concussion – with support that fits into your day. Heads helps you track your symptoms, understand what’s happening, and get back on track with expert-backed guidance. |
| Oticon Companion | <https://play.google.com/store/apps/details?id=com.oticon.app> | This app lets you control your hearing aids from your mobile device. Just a note: some features may be available depending on your hearing aid model. Check below for details. |
| The Self Compassion App | <https://play.google.com/store/apps/details?id=uk.co.psyt.compassion> | The Self-Compassion App is for you if you want to feel happier, calmer & more connected. Whether you get caught up in cycles of stress and anxiety, regularly give yourself a hard time or struggle to relax and enjoy the moment – this app can help.  The app includes scientifically-validated practices from Compassion Focused Therapy (CFT), with 50+ tools to help you when life gets hard. Be guided by Drs Chris Irons and Elaine Beaumont – leading authorities on self-compassion, who collectively have 40+ years of experience as therapists and have helped tens of thousands of people. |
| Constant Therapy: Brain Rehab | <https://play.google.com/store/apps/details?id=com.constanttherapy.android.main> | onstant Therapy is an award-winning, science-based cognitive, language, and speech therapy app designed to help people recovering from stroke, traumatic brain injury (TBI), or people living with aphasia, apraxia, dementia, and other neurological conditions. |
| Complete: Wellbeing & Breathe | <https://play.google.com/store/apps/details?id=com.completecoherence.complete.devtest> | Having the energy and the right emotional state at the right time is fundamental to achieving the results you want in your life. This was our motivation behind building our wellbeing app, the Complete App. |
| CBIT In Hand | <https://play.google.com/store/apps/details?id=com.cube.cbit> | Childhood Concussion, Head injury and Acquired Brain Injury support for families and professionals.  - Digital access to support families with childhood concussion / mild brain injury - Access to make ‘in app’ quick and easy referrals for specialist support - Advice, information & support to families and hospital teams - Ability to send support literature and resources direct to a family at home |
| Head Atlas | <https://play.google.com/store/apps/details?id=com.upsurgeon.skullatlas> | |
| HitCheck: Cognitive Assessment | <https://play.google.com/store/apps/details?id=com.jabico.hhitt> | HitCheck is a HIPPA compliant, FDA Registered Class II Medical device that helps Coaches, Administrators, Athletic Trainers and Physicians monitor and manage their athletes’ brain health. Fast, on-location cognitive testing provides immediate results that can be used to help inform removal-from- activity decision making. HitCheck provides sports organizations, educational institutions and healthcare providers with: |
| Rehabit: brain recovery habits | <https://play.google.com/store/apps/details?id=com.neofect.connect> | The key to successful stroke recovery for stroke survivors is habit changes! |
| Con Edison | <https://play.google.com/store/apps/details?id=com.coned.conedison> | We’ve created a fast and secure way to sign in, pay your bill, and get insights on your energy use on the go. |
| Condeco | <https://play.google.com/store/apps/details?id=com.condecosoftware.condeco> | A simple to use app that allows you to book spaces in seconds. With a few taps you can book a workstation or a meeting room, along with other areas like parking, lockers, quiet spaces, breakout zones and more. |
| MindDoc: Mental Health Support | <https://play.google.com/store/apps/details?id=de.moodpath.android> | Embark on your journey towards better mental health with MindDoc. Trusted by over 3 million users worldwide, MindDoc is rated 4.7 stars from 26,000+ reviews, making it the go-to app for mental well-being. |
| Conn's Current Therapy | <https://play.google.com/store/apps/details?id=com.medpresso.Lonestar.conncurther> | Designed for busy primary care specialists and other first-line care providers concise but in-depth format focuses on the most current diagnosis and treatment protocols for common complaints, acute diseases, and chronic illnesses with nearly 350 newly updated topics. |
| Principles of Neural Science |  |  |
| ACT Companion | <https://play.google.com/store/apps/details?id=com.actcompanion> | |
| Coqon | <https://play.google.com/store/apps/details?id=com.smabit.coqon.smarthome> | Intelligent living can be so simple, safe, and comfortable – with the award-winning and certified Coqon Smart Home System from Germany. Coqon offers smart home beginners and already over 3,000 professionals from the trade a platform that leaves nothing to be desired. |
| N-Back Challenge | <https://play.google.com/store/apps/details?id=com.rivuspurus.nbackchallenge> | The N-Back Challenge provides a special type of cognitive training, “dual n-back,” that helps you raise your IQ in just a few minutes per day |
| Consentio | <https://play.google.com/store/apps/details?id=com.consentio> | Consentio is Europe’s leading digital platform that streamlines trading within the fruits & vegetables industry. |
| Chronic Insights symptom diary | <https://play.google.com/store/apps/details?id=com.chronicinsights> | The privacy-first, ad-free, premium symptom diary that people with chronic health conditions deserve, made by a disabled entrepreneur who lives with a painful autoimmune condition called axial spondyloarthritis. |
| Speechify – Text to Speech | <https://play.google.com/store/apps/details?id=com.cliffweitzman.speechify2> | Your Ultimate Text-to-Speech and Speech-to-Text Solution! |
| MMGuardian Child Phone App | <https://play.google.com/store/apps/details?id=com.mmguardian.childapp> | MMGuardian Parental Control app gives parents the ability to set up parental control and us AI to monitor inappropriate pictures, social media, websites, location and much more. |
| Child and Company | <https://play.google.com/store/apps/details?id=sharedesk.net.optixapp.childandco> | Child & Co. offers its members intuitively-designed, flexible co-working space along with part-time child care. Whether you’re transitioning back into full-time work, starting your own passion project, or taking a break from your normal routine, you’ll find Child & Co. inviting and inspiring for you and your little one(s)! |
| Kids'Skills App | <https://play.google.com/store/apps/details?id=org.kidsskills.app> | Dr. Ben Furman has created this app for parents, teachers and other caretakers of children. |
| Child Reward | <https://play.google.com/store/apps/details?id=com.childreward> | ransform your child's daily tasks into a fun and rewarding experience with Child Reward! |
| Cerebral palsy | | |
| STEPtember | <https://play.google.com/store/apps/details?id=au.com.mangochutney.steptember> | Looking for a way to increase your fitness levels, develop new healthy habits and have a great time with friends or workmates? All whilst celebrating diversity and inclusion and raising funds to support people with cerebral palsy? Then look no further, because STEPtember 2023 is here. |
| Avaz AAC | <https://play.google.com/store/apps/details?id=com.avazapp.international.lite> | Avaz AAC is an Augmentative and Alternative Communication App that empowers children & adults with Autism, Cerebral Palsy, Down Syndrome, Aphasia, Apraxia, and individuals with any other condition/cause of speech delays, with a voice of their own. |
| Spoken – Tap to Talk AAC | <https://play.google.com/store/apps/details?id=com.spoken.app> | Never miss out on the conversation again. Spoken is an AAC (augmentative and alternative communication) app designed for teens and adults who have trouble speaking due to nonverbal autism, aphasia, or other speech and language disorders. |
| Adım Adım Konuşuyorum | <https://play.google.com/store/apps/details?id=com.lali.adimadimkonusuyorum> | It is an interactive language and speech therapy training set with voice and touch functionality.  Starting from imitations, the first step of the conversation, sounds, syllables, continue with single and multi-syllable words and proceed to sentences with 3 words. |
| Expressia: AAC and Activities | <https://play.google.com/store/apps/details?id=life.expressia> | Communicate and learn easily and fun with Expressia, a complete app for: Alternative Communication: |
| NeuroNation - Brain Training | <https://play.google.com/store/apps/details?id=air.nn.mobile.app.main> | With NeuroNation scientific brain training you bring your brain day by day on trot. Whether it's weaker memory, dwindling concentration or thinking too slowly - only 15 minutes of training a day can make problems disappear and give your brain new momentum. Join the worldwide community of over 23 million members and treat yourself to a piece of science - right in your pocket. |
| Constant Therapy: Brain Rehab | <https://play.google.com/store/apps/details?id=com.constanttherapy.android.main> | Constant Therapy is an award-winning, science-based cognitive, language, and speech therapy app designed to help people recovering from stroke, traumatic brain injury (TBI), or people living with aphasia, apraxia, dementia, and other neurological conditions |
| Cognitive game: Eidetic memory | <https://play.google.com/store/apps/details?id=com.brandedbrothers.memorytrainer> | Occipital. Concentration games, Memorama, Eidetic memory, Working memory, Math problems, speed, flexibility and problem-solving in Brain Power. Train your brain with Brain Power and explore your congnitive powers.Train your brain with our focus, concentration, problem-solving, mental math, brain teasers, thinking, ADHD, and smart cognitive games and alzheimer games. |
| Parkinson's Cognitive Research | <https://play.google.com/store/apps/details?id=com.cognifit.android.parkinson> | This app is designed for people who wish to participate in scientific studies related to the cognitive symptoms associated with Parkinson’s. |
| Peak – Brain Games & Training | <https://play.google.com/store/apps/details?id=com.brainbow.peak.app> | Peak is your ultimate brain-training app, blending fun and challenge to keep your mind sharp and active. With over 12 million downloads and games developed alongside neuroscientists from top universities like Cambridge and NYU, Peak is the scientifically-backed workout for your brain. |
| Impulse - Brain Training Games | <https://play.google.com/store/apps/details?id=gen.tech.impulse.android> | Train your brain with our focus, concentration, problem-solving, mental math, thinking, and smart games. Keep your mind calm with our relaxing games. Stay on self-improvement and self-growth track with our personality, IQ, emotional intelligence, archetype tests. |
| Lumosity: Brain Training | <https://play.google.com/store/apps/details?id=com.lumoslabs.lumosity> | Lumosity’s cognitive training program is a fun, interactive way to train your brain and learn about how your mind works. Used by over 100 million people worldwide, Lumosity’s program consists of games designed to exercise memory, speed, flexibility and problem-solving. |
| Elevate - Brain Training Games | <https://play.google.com/store/apps/details?id=com.wonder> | Elevate is an award-winning brain trainer that uses fun games and brain teasers to improve vocabulary, speaking abilities, processing speed, memory skills, mental math, and more for adults and students. Each person gets a personalized learning program that adjusts over time to maximize results |
| Train your brain. Coordination | <https://play.google.com/store/apps/details?id=com.tellmewow.senior.coordination> | We present this collection of coordination games to develop and stimulate the hand-eye movement ability. Fun coordination exercises for the whole family to stimulate the mind in a playful way. This game is suitable for all kinds of people, from the youngest to the elderly and senior players. |
| RecoverBrain Language Therapy | <https://play.google.com/store/apps/details?id=com.imagiration.aphasia> | • Deficit-specific, adaptive and personalized • Designed for every cognitive level, from very easy to challenging • Very easy levels are really easy and can be operated by a toddler • The language training module and seven other training modules are completely free – no subscription necessary • No ads • No setup or registration needed |
| CogniFit - Test & Brain Games | <https://play.google.com/store/apps/details?id=com.cognifit.app> | Did you know that you can train your brain? CogniFit makes it easier than ever to train your brain with a series of fun and engaging mental games. Our patented system takes a personalized approach helps to improve your cognitive function from anywhere, whether at home or on the go. Effective technology used by the scientific community, universities, hospitals, families, and medical centers around the world. |
| Self Therapy: Mental Tests | <https://play.google.com/store/apps/details?id=com.tanosapps.meditanos> | Welcome to Self Therapy: Mental Tests, which is always with you to protect your personal development and mental health. Step into the world of self-care and mental health.  Accelerate your self-care and empower yourself with personalized therapy resources, tailored mental tests, comprehensive summaries, and advanced tools to track your progress. Whether you're struggling with the end of ADHD, managing trauma, dealing with depression, or simply aiming to maintain a balanced mood, take on health work with our Self Therapy: Mental Tests, the reliable |
| Brain Spark: Fast Reaction | <https://play.google.com/store/apps/details?id=com.hybriona.brainspark> | Exercise Your Brain! Brain Spark: Fast Reaction is an addictive Arcade game to train your Concentration and Reaction Skills.  Do you want to test your concentration skills then you must play this hyper-casual game. |
| Visual Attention Therapy Lite | <https://play.google.com/store/apps/details?id=com.tactustherapy.vat.lite> | Visual Attention Therapy helps brain injury and stroke survivors, as well as struggling students, to improve scanning abilities. It also helps rehab professionals to assess for neglect and provide more efficient and effective therapy for attention deficits. |
| Concentration training | <https://play.google.com/store/apps/details?id=com.nixgames.concentration> | Are you looking to overcome procrastination and take your concentration, productivity, and focus to the next level? Dive into the "Concentration Training" app, your gateway to personal growth, motivation, and mastering concentration through targeted exercises. |
| Otsimo \| Special Education | <https://play.google.com/store/apps/details?id=com.otsimo.app> | Otsimo is a certified and award-winning educational game application developed for individuals diagnosed with learning disorders, attention deficit disorders, autism, Down syndrome, Aspergers, and other special needs. Otsimo Special Education has been awarded Mom's Choice Awards, Parent's Pick Awards, Education Alliance Finland, the Academics’ Choice Mind-Building Media and Toys Award, |
| memoryOS: Improve Your Memory | <https://play.google.com/store/apps/details?id=com.memoryos.app> | Learn how to memorize any information, supercharge and improve your memory and brain with the most crowdfunded app in Kickstarter’s history!  memoryOS is a completely new and fun approach to enable great memory skills and learn how to memorize anything through visual memory training games. It contains interactive bite-sized lessons by a 2X World Memory Champion and an educational 3D game of Virtual Mind or Palaces - all in one easy-to-use memoryOS app. |
| BrainTap: Brain Fitness | <https://play.google.com/store/apps/details?id=com.braintaptechnology.android> | BrainTap optimizes your brain’s peak potential—anytime, anywhere.  Backed by neuroscience and research, BrainTap is proven to help you sleep, think, and perform better.  After just 20 minutes of BrainTap use, studies have shown a 38.5% decrease in stress! |
| BrainBloom: Brain training app | <https://play.google.com/store/apps/details?id=com.findmy.brainbloom> | Improve your mental health by playing brain exercise games!  BrainBloom is a brain training app designed to enhance your cognitive abilities with logic games, memory training, brain puzzles, adhd games, and skillz games. |
| Brain training | <https://play.google.com/store/apps/details?id=com.iig.synapptico> | Brain games - Synapptico games brings you 15 brain exercises in 5 different Cognitive function categories: Processing speed, Spatial cognition, Problem solving, Focus and Memory. Synapptico's precisely designed brain training is based on the latest trends and studies in the field of Cognitive science. Daily exercise with Synapptico will help your brain elevate to the peak of its abilities. Reaction, memory, attention, concentration, fluid memory & focus are some of the basic brain skills which contribute to IQ score and are put to test in Synapptico. |
| Reflex: Brain reaction | <https://play.google.com/store/apps/details?id=brain.reaction.concentration> | REFLEX is a brain trainer that uses fun games and brain teasers to improve cognitive abilities, mental quickness, processing speed, memory skills, mental math, and more for adults and students. Each person receives a personalized learning program that adjusts over time to maximize results. Train your brain to focus and respond quickly to stimuli |
| Brainwell - Brain Training | <https://play.google.com/store/apps/details?id=com.monclarity.brainwell> | Boost your cognitive skills with Brainwell - Brain Training Games. |
| My Aphasia Coach | <https://play.google.com/store/apps/details?id=com.myaphasiacoach> | My Aphasia Coach is a cognitive and aphasia speech therapy tool for patients recovering from stroke. The app is free to download and users get instant access to the entire catalog of tools and features included in the complimentary 14-day free trial. |
| Neurobics: 60 Brain Games | <https://play.google.com/store/apps/details?id=com.peoresnada.mental> | Attention! Do you want to enhance your cognitive abilities while having fun? We have the perfect solution for you! Discover our free Brain Training game app: "Neurobics: 60 Brain Games" It's a fun and challenging way to train your brain and improve your mental skills. |
| Brainia: Brain Training Games | <https://play.google.com/store/apps/details?id=com.firstcenturythinking.braintraining> | Brainia : Brain Training Games For The Mind is a collection of 35 brain training games designed to flex your mind using logic, memory, math, words and speed educational games. Perfect for road trips, waiting rooms, or any other time you need a little brain coffee. Games can be played in 60-120 seconds. |
| Spina Bifida | | |
| Spina Bifida Association | <https://play.google.com/store/apps/details?id=com.spinabifida&hl=en> | The Spina Bifida Association (SBA) serves adults and children who live with the challenges of Spina Bifida. Since 1973, SBA has been the only national voluntary health agency solely dedicated to enhancing the lives of those with Spina Bifida and those whose lives are touched by this challenging birth defect. SBA has a presence in more than 125 communities nationwide and touches thousands of people each year. |
| MySpine: Post-Surgery Recovery | <https://play.google.com/store/apps/details?id=org.marker.MySpine> | MySpine prepares and guides you through recovery after the most common spinal surgeries. It is recommended to use it from one month before, up to one year after surgery. |
| Spin Break: Roulette Battle | <https://play.google.com/store/apps/details?id=com.FiftyOnePercent.SpinBreak1> | ake deck-building roguelikes to the next level with Spin Break's fast-paced roulette combat! ■ Dive into a vibrant pixel-art world with a fresh twist on deck-building roguelikes! Spin the wheel, pick your cards, and unleash powerful moves in intense monster battles. |
| Spinal Cord Injury | | |
| Hydrocephalus | | |
| BrainHQ | <https://play.google.com/store/apps/details?id=com.positscience.brainhq.app> | Train your brain with BrainHQ from Posit Science—the most rigorous program available for better brain health, and the only one backed by more than 100 scientific papers showing benefits. Now with new features that contribute to your brain fitness success! |
| EncephalApp - Stroop Test | <https://play.google.com/store/apps/details?id=com.mobelux.stroop_android> | |
| HydroAssist | <https://play.google.com/store/apps/details?id=com.mediakube.ha.hydroassist> | HydroAssist® is the first mobile app that allows you to take your entire hydrocephalus medical treatment history and symptom diary with you to access when needed. Created by medical professionals and an award-winning app developer, HydroAssist is the easiest way to track your surgeries, shunt settings, imaging scans, and symptoms over time. NEW Symptom Diary with Headache Tracker lets you keep track of your symptoms and share them vie email or text with a loved one or your doctor. |
| HC&ME | <https://play.google.com/store/apps/details?id=com.miethke.HC_ME> | HC&ME is a digital hydrocephalus diary that provides a comprehensive documentation of life with hydrocephalus and everyday life with a shunt: |
| Meningomyelocele | | |
| Neurological disease youth | | |
| Symptom, Mood & Period Tracker | <https://play.google.com/store/apps/details?id=com.bearable> | Bearable helps people reclaim control of their well-being by making mood and symptom tracking simple, convenient, and accessible. Making entries into our symptom & mood tracker is effortless, so you can focus on feeling better. |
| Brain Injury | | |
| Beynex - Brain Health Coach | <https://play.google.com/store/apps/details?id=com.beygel.beynex> | Beynex, developed by neurologists and cognitive psychologists, continuously monitors your cognitive abilities and daily habits to provide personalized strategies for your unique brain. A personal coach to assist you on your journey towards a sharp brain, strong memory, and boosted productivity. |
| BrainHealth® | <https://play.google.com/store/apps/details?id=org.brainhealth.bhp_android> | The BrainHealth® App, developed by the Center for BrainHealth® at The University of Texas at Dallas, is a powerful tool making cutting-edge brain health research more easily accessible. Our nonprofit research institute works to redefine understanding and potential enhancement of brain health and performance. The BrainHealth® App brings opportunities to the palm of citizen scientists’ hands so they can support our goal of better understanding how to measure, track, and even improve brain health throughout life. |
| BrainBloom: Brain training app | <https://play.google.com/store/apps/details?id=com.findmy.brainbloom> | BrainBloom is a brain training app designed to enhance your cognitive abilities with logic games, memory training, brain puzzles, adhd games, and skillz games. |
| Locus - Brain Training | <https://play.google.com/store/apps/details?id=com.brain.locus> | Embark on a unique brain training and learning journey with Locus, the all-in-one mobile app designed to elevate your problem-solving, memory, language, focus and searching skills. Immerse yourself in a collection of engaging mini-games, each meticulously crafted to stimulate and challenge your mind. |
| Brain break: Relax + recharge | <https://play.google.com/store/apps/details?id=com.wander120.app> | Wander120 is designed to activate the brain’s regular resting state for refreshed and re-energized thinking. It was less than 25 years |
| Brain Games - Left vs Right | <https://play.google.com/store/apps/details?id=com.mochibits.google.leftvsright> | Playing Left vs Right only 10 minutes daily can keep your mind sharp and help prepare you for a day full of challenges and new problems to solve! |
| Brainwell - Brain Training | <https://play.google.com/store/apps/details?id=com.monclarity.brainwell> | Boost your cognitive skills with Brainwell - Brain Training Games. |
| Elevate - Brain Training Games | <https://play.google.com/store/apps/details?id=com.wonder> | Elevate is an award-winning brain trainer that uses fun games and brain teasers to improve vocabulary, speaking abilities, processing speed, memory skills, mental math, and more for adults and students. Each person gets a personalized learning program that adjusts over time to maximize results. |
| Brain training | <https://play.google.com/store/apps/details?id=com.iig.synapptico> | Brain games - Synapptico games brings you 15 brain exercises in 5 different Cognitive function categories: Processing speed, Spatial cognition, Problem solving, Focus and Memory. Synapptico's precisely designed brain training is based on the latest trends and studies in the field of Cognitive science. Daily exercise with Synapptico will help your brain elevate to the peak of its abilities. Reaction, memory, attention, concentration, fluid memory & focus are some of the basic brain skills which contribute to IQ score and are put to test in Synapptico. |
| Impulse - Brain Training Games | <https://play.google.com/store/apps/details?id=gen.tech.impulse.android> | Train your brain with our focus, concentration, problem-solving, mental math, thinking, and smart games. Keep your mind calm with our relaxing games. Stay on self-improvement and self-growth track with our personality, IQ, emotional intelligence, archetype tests. |
| Brain Injury | <https://play.google.com/store/apps/details?id=com.kihdapps.brain_injury> | The Brain Injury Strategies App is intended to provide ideas for supporting an individual who is returning to learning. It addresses various areas of concern that can happen after a concussion, a type of traumatic brain injury. The end product is a summary of selected strategies that can be implemented by an educator for an individual student. |
| Headache Tracker - Brain Twin | <https://play.google.com/store/apps/details?id=com.nordicbraintech.BrainTwin> | Take Control of Your Migraines and Headaches with Our Headache and Migraine Tracker! |
