# Supplementary material for: Mobile Apps to Enhance Health Literacy in Children and Adolescents With Injury or Disease to the Brain or Spinal Cord: Scoping Review
Source: JMIR Pediatr Parent. 2026 Nov 13;9:e101750. doi: 10.2196/101750 (PMC13762682; doi:10.2196/101750)
Supplement: Supplementary file 7 [file pediatrics-v9-e101750-s006.docx]

**Multimedia Appendix 6. Selected data items.**

Selected data items for review papers, papers with one or more interventions and mobile applications

| **Review papers** | **Papers with one or more interventions** | **Mobile applications** |
| --- | --- | --- |
| Source ID | Source ID | Source ID |
| Authors, country | Authors, country | Title (app store URL^a^) |
| Publication year | Publication year | Website with further information |
| Title (DOI/URL) | Title (DOI^b^/URL) | Developer, country |
|  | Type of source | Release year - year of last version |
| Study design | Study design | Version number |
| Language | Language | Type of source |
| Target injury/disease (brain injury/disease, spinal cord injury/disease, both) | Target injury/disease (brain injury/disease, spinal cord injury/disease, both) | Language |
| Main aim | Main aim | Availability |
| Inclusion/exclusion criteria |  |  |
|  | Intervention | Cost |
|  | Time frame of intervention |  |
| Digital tool(s) used | Digital tool(s) used | Affiliated with a professional medical/health association, charity or government body |
| Number of included sources (n) |  | Target injury/disease (brain injury/disease, spinal cord injury/disease, both) |
|  | Outcomes of relevance/ learning features | Age |
|  | Population (n, mean age ±SD^c^) | User purpose |
|  | Setting (home, institution, mixed) | Features |
|  | Health personnel involvement (yes/no) | Setting for use (home, institution, mixed) |
| Main findings of relevance | Main findings of relevance | Health personell involvement (yes/no) |
|  |  | Data security statement in App store |
|  |  | Data security statement in Google Play |
|  |  | Refers to privacy policy from developers’ site (yes/no) |
|  |  | Third-party authorizations (yes/no) |
|  |  | Data can be integrated into EHR^d^ (yes/no) |
| ^a^ URL: Uniform Resource Locator, ^b^ DOI: Digital Object Identifier, ^c^ EHR: Electronic Health Record, ^d^ SD: Standard Deviation | | |
